# Covalent Protein Inhibitors via Tyrosine and Tryptophan Conjugation with Cyclic Imine Mannich Electrophiles

**DOI:** 10.1002/anie.202516630

**Published:** 2026-01-28

**Authors:** Sijie Wang, Lei Wang, Marco Hadisurya, Siavash Shahbazi Nia, W. Andy Tao, Emily C. Dykhuizen, Casey J. Krusemark

**Affiliations:** ^1^ Department of Medicinal Chemistry and Molecular Pharmacology Purdue University 575 Stadium Mall Drive West Lafayette IN 47907 USA; ^2^ Department of Biochemistry Purdue University 175 South University Street West Lafayette IN 47907 USA; ^3^ Purdue Center for Cancer Research Purdue University 201 South University Street West Lafayette IN 47907 USA; ^4^ Present address: WuXi AppTec 3 Dean Road, 2nd Floor Natick MA 01760 USA

**Keywords:** Mannich reaction, Targeted covalent inhibitors, Tryptophan labeling, Tyrosine labeling

## Abstract

Targeted covalent inhibitors (TCIs) are increasingly popular as drug candidates and chemical probes. Among current TCIs, the chemistry is largely limited to cysteine and lysine side chain reactivity. Here, we investigated the utility of cyclic imine Mannich electrophiles as covalent warheads to target protein tyrosine and tryptophan side chains. We characterized the intrinsic reaction rates of several cyclic imines to tyrosine and other amino acid side chains and validated reactivity using protein affinity labeling of a cyclic imine‐modified trimethoprim with tyrosine and tryptophan mutants of *E. coli* dihydrofolate reductase. To validate the utility of the approach, we appended cyclic imine warheads to a CBX8 chromodomain inhibitor to label a non‐conserved tyrosine, which improved both the potency and selectivity of the inhibitor for CBX8 in vitro and in cells. These findings indicate that Mannich electrophiles are promising and robust chemical warheads for tyrosine and tryptophan bioconjugation and development of covalent inhibitors.

## Graphical abstract

Targeted covalent inhibitors (TCI) containing cyclic imine warheads react with tyrosine and tryptophan residues via the Mannich reaction. These cyclic imine warheads show comparable reaction kinetics to cysteine‐reactive electrophiles commonly used in TCIs. Tyrosine‐reactive TCIs were developed from an inhibitor of the CBX8 chromodomain and demonstrated increased potency, selective proteome‐wide labeling, and improved cellular activity. This strategy will expand the capabilities of TCIs for targeting both tyrosine and tryptophan.

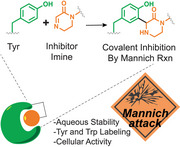

The number and diversity of covalent inhibitors have increased dramatically in recent years.^[^ ^1^ ^]^ The majority of these are targeted covalent inhibitors (TCIs), which position a reactive moiety (typically an electrophile of low reactivity) adjacent to an amino acid side chain, inducing a high effective concentration. This leads to a selective conjugation to a proximal amino acid side chain to yield an inhibitor with covalent occupancy.^[^ ^2^ ^]^ Compared with noncovalent inhibitors, covalent inhibitors can exhibit enhanced selectivity, increased potency, and prolonged modulation of target function. Notably, covalent conjugation has enabled inhibition of protein–protein interactions (PPIs) previously deemed undruggable.^[^ ^3^, ^4^, ^5^ ^]^

Chemistry for covalent inhibitor conjugation is currently dominated by thiol‐reactive electrophiles that react with cysteine side chains.^[^ ^6^ ^]^ Cysteines, however, are rare amino acids (1.4% of amino acids^[^ ^7^ ^]^) that are poorly represented at PPIs.^[^ ^8^, ^9^ ^]^ Development of chemical warheads for targeting other residues will further expand the utility of TCIs. Tyrosine and tryptophan are attractive targets due to participation in noncovalent interactions, including hydrogen bonding, cation‐π or π–π interactions, and hydrophobic interactions.^[^ ^10^, ^11^ ^]^ While tyrosine and tryptophan are also of low abundance (3.2% and 1.1%, respectively^[^ ^12^ ^]^), they are enriched at PPIs, at predicted hot spots on proteins, and at protein functional sites.^[^ ^8^, ^9^, ^13^, ^14^ ^]^

There are many examples of TCIs that react with tyrosine, yet there are no approved drugs. The chemistry has largely been limited to sulfonyl fluorides, fluorosulfonates, and sulfonyl azoles, which react with the phenolic oxygen of tyrosine.^[^ ^15^, ^16^, ^17^, ^18^ ^]^ These warheads exhibit no or modest selectivity for tyrosine, which can be critical for many applications. They also modify lysine, cysteine, serine, threonine, and histidine.^[^ ^17^, ^19^, ^20^ ^]^ Stability issues due to hydrolysis and thiolysis of these warheads can limit their utility, as can off‐target activity with serine/threonine hydrolases.^[^ ^21^, ^22^, ^23^ ^]^ Suitable chemistry for targeting tryptophan with covalent inhibitors is lacking, and there are no TCIs targeting tryptophan to date. Most bioconjugation chemistry for tryptophan requires the addition of exogenous reagents or harsh conditions or is sensitive to the presence of thiols.^[^ ^13^, ^24^, ^25^ ^]^ Thiazolinedione (TAD) reagents show rapid reactivity and Tyr/Trp selectivity under mild conditions but suffer from poor aqueous stability and decompose to isocyanates, which react with additional residues (e.g., Lys/Cys).^[^ ^26^, ^27^ ^]^ Similarly, aryl diazonium salts have shown utility in tyrosine conjugation but also have high reactivity and poor stability.^[^ ^28^, ^29^, ^30^ ^]^

In 2004, the Francis group reported a three‐component reaction for the selective modification of protein tyrosines and tryptophans via the Betti variant of the Mannich reaction.^[^ ^31^, ^32^, ^33^ ^]^ Subsequently, the development of a two‐component reaction by the Tanaka Lab using a pre‐formed, cyclic imine (an iminolactam) increased reactivity to phenols over a broad pH range.^[^ ^34^ ^]^ In the present work, we utilize two‐component Mannich chemistry for modification of a tyrosine side chain using a TCI with demonstrated in vitro and cellular efficacy (Figure 1). We first explore the intrinsic reactivity of various cyclic imine derivatives with amino acid side chains and then append Mannich electrophiles to inhibitors of the CBX8 chromodomain to create TCIs that react with a nearby tyrosine. The resulting compounds exhibit cellular efficacy and proteome‐wide selectivity, indicating cyclic imines as promising chemo‐selective warheads to label tyrosine or tryptophan.

**Figure 1:**
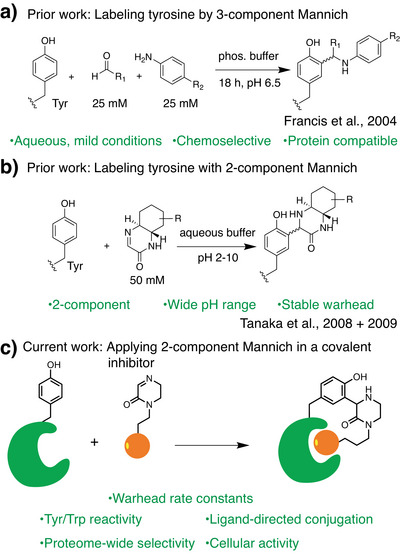
Prior and current work using the Mannich reaction for protein labeling.

To identify an appropriate warhead, we synthesized or purchased a collection of cyclic imines and incubated them with N‐acetyl‐l‐tyrosine methylamide (Ac‐Tyr‐NHMe) in PBS pH 7 at 37 °C (Table 1). The reactivity was quantified by UV integration on HPLC with LC/MS confirmation of product identity (Figure S1). Under pseudo‐first‐order reaction conditions, we determined that the reaction half‐life (*t*_1/2_) of imine **1** was 8 ± 0.3 h with a second‐order rate constant of 2.4 × 10^−4^ M^−1^ s^−1^ at 37 °C, similar to what was previously reported by Tanaka et al.^[^ ^34^ ^]^ (Table 1; Figures 2 and S2). This reaction rate compares favorably to the range of intrinsic rates of acrylamide electrophiles to cysteine (1 × 10^−2^ to 1 × 10^−4^ M^−1^ s^−1^), which are the most used reactive groups in covalent drugs.^[^ ^35^ ^]^ Iminolactones **2** and **3** showed increased reactivity, yet suffered from competing hydrolysis of the ester. The hydrolysis kinetics of imine **2** were characterized at pD 6 and 7.5 by NMR (Figure S3). Further increasing electron‐withdrawing properties of the amide in aromatic lactam **4** increased the rate slightly. The Mannich reaction mechanism involves a charged iminium intermediate, and the reaction is frequently catalyzed by Bronsted acids.^[^ ^36^, ^37^ ^]^ NMR analysis of compound **1** in phosphate‐buffered D_2_O at pD 7 is indicative of the unprotonated imine as the major species (see Appendix 1)^[^ ^38^ ^]^ leading us to hypothesize that iminium **5** would have improved reactivity at neutral pH. In fact, the opposite was true. Also, commercially available dihydro‐beta‐carbolines showed either very weak (compound **6**) or no reactivity (compound **7**). Sulfamide imine **11** showed a marked increase in rate with a t_1/2_ of 2.4 ± 0.1 h (k_2_ = 8.1. × 10^−4^ M^−1^ s^−1^), but sulfonate ester imine **12** was not stable in aqueous solution. Other imines showed limited (compound **8**) or no (compounds **9**, **10**) reactivity to tyrosine, as summarized in Table 1. To facilitate comparison, we additionally tested sulfonyl fluoride and fluorosulfonate warheads in compounds **13** and **14**. Compound **13** (commonly used serine hydrolase inhibitor 4‐(2‐aminoethyl)‐benzenesulfonyl fluoride (AEBSF)) showed the highest reactivity tested (*t*_1/2_ of 0.28 ± 0.02 h; k_2_ = 6.9 × 10^−3^ M^−1^ s^−1^); however, at pH 7 and 37 °C, AEBSF has a hydrolysis half‐life of 6 h.^[^ ^39^ ^]^ While fluorosulfonates like **14** are more stable to hydrolysis,^[^ ^40^ ^]^ they show low reactivity (Table 1).

**Table 1:** Reaction of cyclic imine Mannich or sulfur fluoride electrophiles (5 mM) with *N*‐acetyl‐l‐tyrosine methyl amide (100 mM) in PBS pH 7 at 37 °C. Reaction progress was monitored by LC/MS.

compound	reactivity (t_1/2_)	compound	reactivity (t_1/2_)
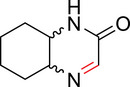 **1** ^a)^	8 ± 0.3 h	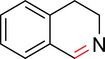 **8**	5% conv. in 24 h
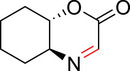 **2**	∼40 mins^b)^	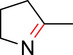 **9**	no reactivity
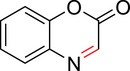 **3**	30% conv. in 30 mins^b)^	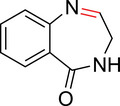 **10**	no reactivity
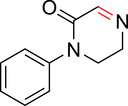 **4**	6.1 ± 0.2 h	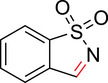 **11**	2.4 ± 0.1 h
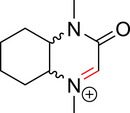 **5** ^a)^	33 ± 3 h	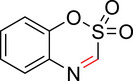 **12**	not stable
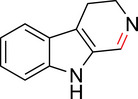 **6**	30% conv. in 72 h	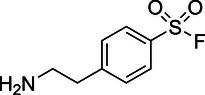 **13**	0.28 ± 0.02 h
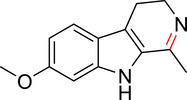 **7**	no reactivity	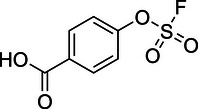 **14**	∼48 h

^a)^ mixture of trans isomers.

^b)^ Competing hydrolysis.

**Figure 2:**
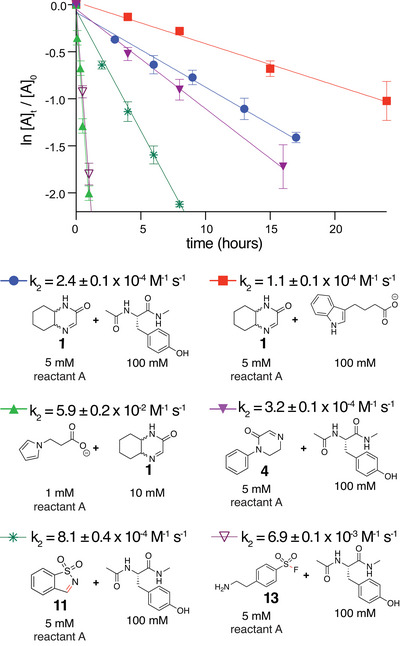
Second‐order rate constants for select cyclic imines and a sulfonyl fluoride with aromatic nucleophiles. Reactions were conducted under pseudo‐first‐order conditions with an excess of one reactant, as indicated. The reduction in the concentration of the limiting reactant was measured at various time points by HPLC analysis. Rates of imine **1** with N‐Ac‐Tyr‐NMe, indole‐3‐butyric acid, and 1*H*‐pyrrole‐1‐propionic acid and compounds **4**, **11**, and **13** with N‐Ac‐Tyr‐NMe were measured.

To assess the selectivity for tyrosine side chains, we tested the reactivity of **1** with additional compounds, including β‐mercaptoethanol, imidazole, isopropanol, n‐butylamine, propionic acid, 3‐methyl indole, and N‐ethyl pyrrole. We observed stable conjugation of **1** with thiol, indole, and pyrrole groups using LC/MS analysis (Figure S4). While partially stable under LC/MS conditions, the *N,S*‐thioaminal product is readily reversible.^[^ ^41^ ^]^ The tryptophan mimic, 3‐methyl indole, reacted with **1** to form the C2‐modified Pictet‐Spengler‐type addition product, which was confirmed by NMR (Appendix 1).^[^ ^42^ ^]^ The poor solubility of 3‐methyl indole complicated rate constant determination and may account for discrepancies with a prior report that did not observe reactivity with imine **1**.^[^ ^34^ ^]^ Using indole‐3‐butyrate as a mimic with enhanced solubility, we determined the rate constant (*k*_2_ = 1.1 × 10^−4^ M^−1^ s^−1^ at 37 °C) (Figures 2 and S2), which was only 2‐fold slower than tyrosine. We also tested **1** with N‐ethyl pyrrole as a mimic of pyrrolated lysine, a lesser‐known posttranslational modification.^[^ ^43^, ^44^ ^]^ We determined a second‐order rate constant of 5.9 × 10^−2^ M^−1^ s^−1^ for the reaction of **1** with 1*H*‐pyrrole‐1‐propionate (Figures 2 and S2). This ∼200‐fold increase relative to Tyr suggests cyclic imines may be useful for proteomic profiling of lysine pyrrolation.

While thiol addition to the imine is reversible,^[^ ^34^, ^41^ ^]^ this reaction may limit the desired reactivity of imines to tyrosines in cellular contexts with 1–10 mM glutathione.^[^ ^45^, ^46^ ^]^ To evaluate this reversibility, the pre‐formed thioaminal product of **1** with β‐mercaptoethanol was treated with either excess glutathione (1 M) or N‐Ac‐Tyr‐NHMe (100 mM). Both glutathione and N‐Ac‐Tyr‐NHMe outcompeted the thioaminal product after 24 h (Figure S5). To evaluate potential effects of cellular thiols on tyrosine labeling rate, the cyclic imine‐Tyr reaction (at 5 and 100 mM reactants, respectively) was evaluated over time in the presence of glutathione (5 mM) (Figure S6), which only slightly slowed the reaction rate compared to the control. To validate the Mannich product stability, we tested the product of imine **1** with N‐Ac‐Tyr‐NHMe in PBS, 1% NaOH, and 1% TFA and found excellent stability over 96 h (Figure S7).

To test the suitability of cyclic imines for targeted covalent inhibitors, we synthesized a cyclic imine‐containing derivative of trimethoprim (TMP‐Im) (Figure 3a; Scheme S8), a high‐affinity (K_i_ ∼ 1 nM) inhibitor of *E. coli* dihydrofolate reductase (eDHFR). This ligand‐receptor pair has previously been used as a tag to selectively label fusion proteins in mammalian cells covalently, using TMP‐acrylamide with the L28C mutant of eDHFR. Similarly, we prepared eDHFR tyrosine and tryptophan mutants at position 28 to assess labeling with TMP‐Im (Figure 3b). After overnight incubation, we observed covalent labeling of both the L28Y and L28W proteins by LC/MS analysis with no labeling of the wild type (Figure 3c). Longer‐term labeling of the L28Y mutant for 48 and 72 h showed only marginal increases in labeled protein (Figure S8). We conducted trypsin digestion of the labeled L28Y protein and localized the modification to the intended tyrosine by MS/MS fragmentation (Figure S9A). To assess selectivity over thiols, we tested labeling of the cysteine mutant. Labeling in the presence of 5 mM glutathione (GSH) gave no detectable labeled protein (Figure 3c). A small amount of labeling was detected with incubation in the absence of additional thiols using TCEP as a reductant (Figure S10). This labeling was reversed with GSH treatment, which is consistent with the reversibility of thioaminal formation (Figure S10). Labeling the L28Y mutant in the presence of 5 mM GSH gave only a slight reduction in reaction conversion (Figure S10), which suggests labeling within the cell cytosol is feasible.

**Figure 3:**
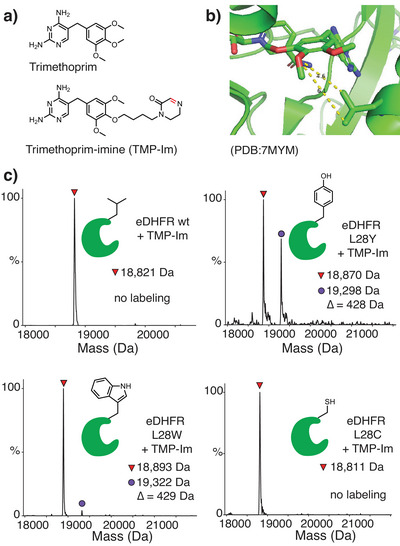
Covalent inhibitors to *E. coli* dihydrofolate reductase (eDHFR) mutants using a cyclic imine‐containing trimethoprim. a) Trimethoprim (TMP) was modified by the para‐methoxy group with a four‐carbon linker to an iminolactam. b) Crystal structure of the eDHFR‐TMP complex (PDB code 7MYM) showing the proximity of the methoxy group with leucine 28. c) Deconvoluted mass spectra of the total protein peak for TMP‐Im reactions with 10 µM eDHFR wt, L28Y, L28W, and L28C and 10 µM compound overnight at 37 °C. Reactions with thiol mutant L28C were conducted in the presence of 5 mM glutathione and 5 mM TCEP. The observed mass differences of labeled and unlabeled proteins agree with the expected mass change of 428 Da.

Our interest in this chemistry was to improve inhibitors of the chromodomains (ChD) of the CBX family of epigenetic proteins, which have proven challenging to inhibit selectively or with drug‐like small molecules.^[^ ^47^ ^]^ The CBX ChDs bind a trimethylated lysine (Kme3) side chain on histone tails via cation‐pi interactions with the aromatic cage in the ChD. Our initial hypothesis was that the aromatic cage tryptophan residues would show increased reactivity to Mannich electrophiles, as they function naturally to donate electron density to the electron‐poor methyl groups of Kme3. To test this, **1** (50 µM) was incubated for 10 h with the CBX8 chromodomain (ChD) (50 µM), and labeling was assessed by mass spectrometry (Figure S11). Single‐site labeling suggestive of high reactivity to aromatic cage residues W32 or W35 was not observed. Instead, there was a distribution of unlabeled, singly, doubly, and triply labeled protein indicative of random labeling (Figure S11).

Nonetheless, we observed that the CBX8 ChD contained a tyrosine residue (Y39) (Figure 4a) adjacent to the aromatic cage (Figure 4b) that could be targeted for covalent inhibition. In prior work, we developed **SW2_110A**, a CBX8 ChD inhibitor with an 800 nM *K*_d_ (Figure 4c).^[^ ^47^, ^48^ ^]^ Although **SW2_110A** demonstrates high selectivity for CBX8 over CBX4, CBX6, and CBX7, it is only 6‐fold selective over CBX2. Since CBX2 has a nonreactive histidine at position 39, a covalent inhibitor targeting Y39 in CBX8 would improve selectivity (Figure 4a). The crystal structure of a similar ligand (**UNC3866**) bound to CBX8 (Figure 4b)^[^ ^49^ ^]^ indicated that Y39 is ∼8–9 Å from the lysine ε nitrogen and that one of the ethyl groups off the lysine nitrogen is solvent exposed (Figure 4b). Thus, we synthesized several potential covalent ligands by linking warheads off an ethyl group of **SW2_110A**. (Figure 4c).

**Figure 4:**
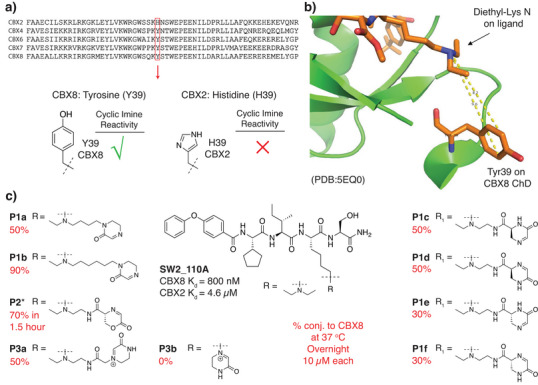
Design of covalent inhibitors to the CBX8 chromodomain (ChD). a) Sequence alignment of the five CBX ChDs in the Polycomb repressive complex 1 shows a histidine in CBX2 at position 39 that allows the reactivity of the tyrosine in CBX8 to be exploited for improved inhibitor specificity. b) Crystal structure of peptide ligand UNC3866 bound to CBX8 ChD (PDB code 5EQ0) with Tyr39 adjacent to the diethyllysine group. c) Synthesized derivatives of **SW2_110A** containing cyclic imine or iminium warheads. Percent covalent conjugation was determined by UV integration on LC/MS after overnight incubation at 10 µM compound and protein at 37 °C.

The analogs were synthesized using standard, solid‐phase peptide synthesis of **SW2_110A** with a nosyl‐protected lysine (Schemes S11–S19), which was further elaborated on‐resin by alkylation with an alkyl halide or Fukuyama–Mitsunobu chemistry.^[^ ^50^ ^]^ We prepared warheads as a primary amine suitably placed adjacent to a serine amide or ester as a latent electrophile. After purification, the compounds were treated in situ with sodium periodate to oxidize the 1,2‐amino alcohol of serine to produce an alpha‐aldehyde amide/ester, which cyclizes to the desired imines.

Cyclic imine ligands were tested for ligand‐induced labeling with CBX8 ChD. The labeling of CBX8 ChD was quantified by a peak shift on LC/MS (Figures S12–S14). Among the lactam series (**P1's**), compound **P1b**, with a 6‐carbon linker, gave the best yield for labeling the CBX8 ChD (∼90%). Deconvoluted mass spectra showed expected mass shifts (example spectrum shown in Figure 5a). **P1b** was also tested in the presence of 10 mM GSH, with no effect on yield (Figure S10). Due to the enhanced reactivity of iminolactone (warhead **2**), labeling with ester imine inhibitor **P2** was faster, with 70% conversion in 1.5 h (Figure S14). We additionally prepared two iminium warhead compounds, **P3a** and **P3b**. Despite the low intrinsic reactivity observed with model iminium **3** and Tyr (Table 1), inhibitor **P3a** gave a labeling yield (50%) that was comparable to the more reactive iminolactams, perhaps through assistance from the local environment on the protein. **P3b** placed the iminium warhead directly on the ε nitrogen of lysine (rather than toward Y39) as an attempt to label aromatic cage residues W32 and W35, which was unsuccessful.

**Figure 5:**
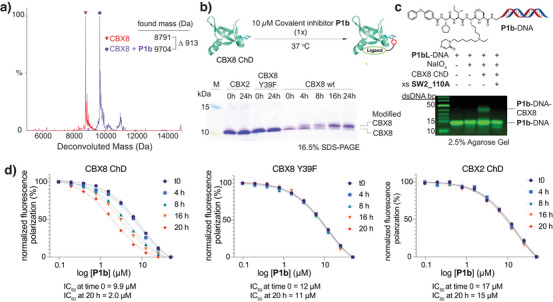
Characterization of ligand‐directed protein labeling with covalent inhibitor **P1b**. a) Deconvoluted mass spectra of CBX8 ChD treated overnight with **P1b** (purple) and control CBX8 ChD (red). Observed masses are shown (expected average mass for CBX8 ChD = 8792 Da). The observed mass difference (913 Da) agrees with the expected mass change of 913 Da. b) Labeled protein was detected by gel shift on 16.5% Tris‐Tricine SDS‐PAGE. CBX8 wild type (wt), Y39F mutant, or CBX2 ChDs were incubated at 10 µM with equimolar amounts of **P1b** for indicated times at 37 °C prior to gel analysis. c) A **P1b‐**DNA conjugate was generated in situ by treatment of **P1bL**‐DNA at 10 with 100 µM NaIO_4_ and incubation with CBX8 ChD at one equivalent overnight at 37 °C. An excess (10‐fold, 100 µM) of noncovalent ligand **SW2_110A** was used to compete with protein labeling. Gel analysis was conducted on a 2.5% agarose gel in 10 mM sodium borate buffer. DNA was stained with GelStar (Lonza), and a fluorescence gel image is shown. d) Time‐dependent fluorescence polarization displacement assays of indicated chromodomains with **P1b**. Assays were conducted using **SW2_113** as an FP probe (Figure S20) at 100 nM. All proteins were used at 2 µM in PBS (pH 7), 0.02% Tween 20. Error bars represent SD.

To be suitable for chemical probe and therapeutic applications, the warhead should show good stability. In general, we found the iminolactam warheads to be quite stable. Compound **1** was stable as a solid at room temperature for over 3 months. We tested the stability of **TMP‐Im** and **P1b** to long‐term incubation in PBS. After 24‐h in aqueous buffer, compounds were treated with sodium cyanoborohydride. Mass spectrometry indicated full reduction of the imine in both compounds, indicating stability over the 24 h (Figure S15). The synthetic strategy of in situ formation of imines by periodate treatment was pursued to avoid concerns of warhead reactivity with the ligand itself to either the peptide backbone or the phenoxyphenyl aryl group. This appears to have been unnecessary, at least for the iminolactam warhead. Despite the hydrolytic instability of the iminolactone **2**, we found peptide inhibitor **P2** to be resistant to hydrolysis (Figure S16). LC/MS analysis showed a mass of the imine hydrate; however, this compound was resistant to cyanoborohydride reduction. The more reactive ester imine may be forming semi‐stable cyclized products with the amide backbone, which have been observed previously with peptide aldehydes.^[^ ^51^, ^52^ ^]^ Due to these issues, we selected compound **P1b** for further characterization. Prior to pursuing biological experiments, we additionally tested the **TMP‐Im** and **P1b** stability in the presence of bovine serum‐containing cell media (15% serum). We observed minimal loss of compounds to the precipitated protein fraction or degradation after 24‐h incubation as assessed by LC/MS (Figure S17).

We confirmed protein labeling through a gel shift assay on Tris‐Tricine SDS‐PAGE (Figure 5b). CBX8 showed time‐dependent labeling with **P1b** with a *t*_1/2_ of approximately 6 h. To validate labeling of the targeted tyrosine residue (Y39), this amino acid was mutated to phenylalanine. Both the gel shift assay and LC/MS protein labeling experiments (Figure S18) indicate that the covalent inhibitor fails to label the mutant CBX8 Y39F. Similarly, for CBX2 ChD with histidine at position 39, no labeled protein was detected by gel shift or MS (data not shown), supporting the ligand‐directed labeling of Y39 on CBX8. We additionally confirmed the labeling site as Y39 by trypsin digest and LC‐MS/MS analysis (Figure S9B).

Given the observed labeling rate and the reported affinity of **SW2_110A**, we determined the *k*_inact_/*K*_i_ for the **P1b** covalent inhibitor at 40 M^−1^ s^−1^. This value represents a very modest rate increase over the non‐directed reaction rate in comparison to typical TCIs,^[^ ^53^ ^]^ which suggests that much improved rates could be achieved by further optimizing the placement of the imine relative to the reactive tyrosine. The rigid and planar nature of both the tyrosine nucleophile and the cyclic imine electrophile may present challenges generally for optimizing orientation in a TCI. In this case, it was no surprise that spanning the distance from the ligand binding site to Y39 with a flexible, six‐carbon linker results in a less‐than‐ideal increase in effective concentration.

DNA‐encoded chemical libraries (DELs) are a useful tool for medicinal chemistry optimization of compounds.^[^ ^47^ ^]^ We sought to explore the potential utility of the iminolactam warhead within DNA‐linked compounds. We synthesized the warhead on an amino‐modified DNA (Scheme S20). Treatment of this product with Ac‐Tyr‐NHMe at 100 mM gave the Mannich product. The rate was slowed relative to imine **1**, and complete overnight conversion required increasing the temperature to 50 °C (Figure S20). This rate reduction is likely due to reversible adducts with DNA bases, which are known to form with cyclic imine‐containing, pyrrolobenzodiazepine natural products such as anthramycin.^[^ ^54^ ^]^ Similarly, a DNA conjugate of inhibitor **P1b** was prepared (Scheme S21). Agarose gel shift analysis showed successful labeling of the CBX8 ChD protein with the DNA‐inhibitor conjugate (Figure 5c), indicating the potential utility of this warhead with DELs.

To evaluate the covalent binding of the cyclic imine warhead‐containing inhibitor **P1b**, we used a fluorescence polarization (FP) displacement assay. Without additional incubation time, the noncovalent inhibitor **SW2_110A** and **P1b** have comparable IC_50_ values (Figure S20), which suggests that the structural modification of the warhead does not significantly change the affinity. With further incubation, **P1b** displayed a time‐dependent increase of potency (decrease of IC_50_), while noncovalent inhibitor **SW2_110A** did not (Figures 5d and S20). To validate protein stability over the incubation time of the assay, the *K*_d_ of the FP probe (**SW2_123**) was measured at time 0 and 20 h. The *K*_d_ was unchanged, indicating good stability (Figure S21). The fluorescence displacement assay indicated **P1b** binds to wild‐type or mutant CBX8 (Y39F) with comparable IC_50_ values (9.9 versus 12 µM, respectively) (Figure 5d); however, no change in IC_50_ was observed upon extended incubation with the CBX8 Y39F mutant. Similarly, the time‐dependent FP assay showed no change in potency for binding to CBX2.

To assess covalent labeling in proteomic context, we prepared a C‐terminal, propargyl amide derivative of **P1b** (**P1bP**) (Figure 6a). After incubation in HEK293T cell lysates, click chemistry was conducted with TAMRA‐azide, and labeled proteins were observed by in‐gel fluorescence (Figure 6a,b). Several proteins were labeled with two prominent fluorescent bands. We observed a dose dependency of labeling of CBX8 with the covalent inhibitor from 10 to 1000 nM inhibitor concentration (Figure 6a). With co‐incubation of excess noncovalent inhibitor **SW2_110A** (∼20 µM, 20 eq), protein labeling was reduced throughout, suggesting the bulk of the protein labeling observed is ligand dependent (Figure 6b). We also synthesized a control cyclic imine alkyne (**15**) to assess proteomic labeling. Concentrations of 100 µM to 1 mM were required to achieve comparable levels of labeled proteins, which suggests the observed labeling with **P1bP** is ligand‐directed.

**Figure 6:**
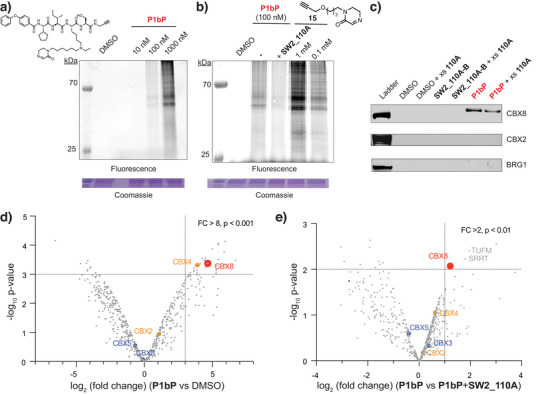
Proteomic activity of covalent inhibitors for CBX8. a) SDS‐PAGE, in‐gel fluorescence analysis of HEK293T lysates treated with **P1bP** at 10, 100, or 1000 nM and Cu‐catalyzed azide‐alkyne cycloaddition (CuCAA) to azide‐TAMRA. b) Additional SDS‐PAGE, fluorescence analysis of HEK293T lysates as in (a) with **P1b** at 100 nM with or without competing **SW2_110A** at 10 µM. Proteomic labeling was also conducted with a control, alkyne‐containing iminolactam (**15**) at 0.1 and 1 mM concentrations. c) Chemoprecipitations from HEK293T cell lysates using biotin‐labeled **SW2_110A** (**SW2_110A‐B**) and biotin‐labeled **P1b** (**P1b‐B**) at 1 µM were performed with stringent washing conditions (modified RIPA buffer: 50 mM Tris‐HCl, 150 mM NaCl, 1 mM EDTA, 1% NP‐40, 0.1% DOC) and analyzed for protein enrichment by immunoblot. “+ xs **110A”** indicates the addition of **SW2_110A** at 20 µM. BRG1 is non‐associated nuclear protein included as control for non‐specific binding d,e) Volcano plots of streptavidin enrichment for proteins identified in **P1b‐B‐**treated 293T lysates versus a DMSO control (d) or in the presence of excess **SW2_110A** (10 µM) (e). N = 2, biological replicates; P values were generated using the limma moderated *t*‐test.^[^ ^55^ ^]^

To confirm that imine inhibitors can covalently capture the endogenous, full‐length CBX8, western blots of pulldown assays were also performed making use of propargyl inhibitor **P1bP** (Figure 6c). After incubation, labeled lysates were clicked to a biotin‐azide for affinity purification. Stringent washing conditions were applied to purify only covalently labeled protein. CBX8 was captured by the covalent inhibitor, while CBX2 and the non‐specific nuclear protein BRG1 were not. An excess of the noncovalent inhibitor was doped in for competition, which reduced covalent labeling of CBX8 by **P1bP**. Denaturing washing conditions of 0.2% SDS (Figure 6c,d) or 6M urea specifically captured CBX8 and no other CBX proteins (Figure S22).

To further evaluate labeling in whole cell proteomes, mass spectrometry analysis was performed after compound pulldown (chemoIP‐MS) (Figure 6d,e). CBX8 was robustly enriched (∼64‐fold) using the biotinylated, covalent inhibitor. Of the seven proteins significantly enriched with **P1bP** (log_2_(**P1bP**/DMSO) > 3, P < 0.001), only CBX8 enrichment was significantly reduced with competing nonreactive ligand **SW2_110A**, indicating ligand‐dependent, covalent labeling of endogenous CBX8 (Figure 6d). Relative enrichment of CBX8 was less under the competition conditions (∼2‐fold) but was consistent with the modest reduction in labeling with competitive free ligand observed by western blot (Figure 6c). Pearson correlation analysis of biological replicates of these experiments was also conducted to confirm reproducibility (Figure S23).

We next assessed the cellular activity of cyclic imine inhibitors using a disease‐relevant cell model. We previously reported that the noncovalent inhibitor **SW2_110A** inhibits CBX8 binding to chromatin and reduces CBX8‐mediated gene expression in MLL‐AF9‐transformed leukemia.^[^ ^47^ ^]^ Using this same model, we compared the covalent inhibitors with noncovalent inhibitor **SW2_110A** in THP1 cell lines. Initially, **P1** series inhibitors and **P3a** were tested at 10 µM for reducing the gene expression of *HOXA9*, a known target of CBX8. The covalent inhibitors (**P1a–f)** and **P3a** reduced expression to a greater extent than the noncovalent inhibitor (Figure S24). Inhibitor **P1b** was the most effective at reducing *HOXA9* gene expression, where 10 µM **P1b** treatment reduced gene expression to a similar extent as 100 µM **SW2_110A** (Figure 7a). We subsequently tested **P1bP** for dose‐dependency in this cellular assay. Dose curves indicate an improvement of cellular activity of the covalent inhibitor compared to the noncovalent inhibitor (Figure 7b). These results are consistent with the covalent linkage contributing to the observed increase in potency.

**Figure 7:**
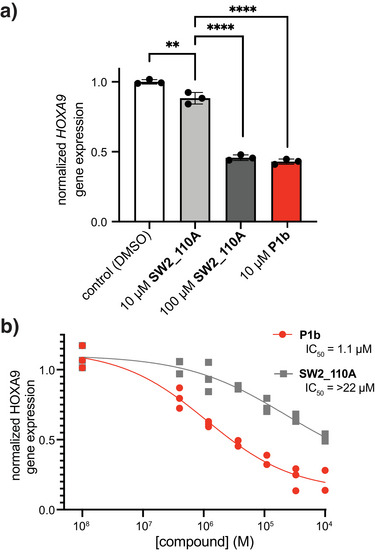
Cellular activity of covalent, cyclic imine inhibitors to CBX8. a) qRT‐PCR analysis of *HOXA9* gene expression in THP1 cells after 48 h of compound treatment at the indicated dose. N = 3. Error bars represent SD. Statistical significance was calculated using Student's *t*‐test. ** = *p* < 0.01, **** = *p* < 0.0001, b) qRT‐PCR analysis of *HOXA9* gene expression in THP1 cells treated with increasing doses of compound for 48 h. N = 3. Error bars represent SD.

In conclusion, we report the development of novel cyclic imine warheads and the first demonstration of ligand‐directed Mannich chemistry for covalent inhibition. We performed quantitative evaluation of reaction rates of cyclic imines to tyrosine and evaluated reactivity to other amino acid functional groups; however, further improvement of warhead properties, such as electronic tuning of imine reactivity, will increase their utility. In addition, this electrophile class will enable chemoproteomic approaches for drug discovery and biological research, such as activity‐based protein profiling and discovery of ligandable tyrosines and tryptophans. Given the limitations of current chemistry for tyrosine and tryptophan modification, this chemistry should find several uses in medicinal chemistry and bioconjugation.

## Conflict of Interests

The authors declare no conflict of interest.

## Supporting information



Supporting Information

## Data Availability

The data that support the findings of this study are available in the supplementary material of this article.
