# Supplementary material for: Covalent Protein Inhibitors via Tyrosine and Tryptophan Conjugation with Cyclic Imine Mannich Electrophiles
Source: Angew Chem Int Ed Engl. 2026 Jan 28;65(15):e16630. doi: 10.1002/anie.202516630 (PMC13053910; doi:10.1002/anie.202516630)
Supplement: Supplementary file 1 — Supporting Information [file ANIE-65-e16630-s001.docx]

Supporting Information

**Covalent Protein Inhibitors via Tyrosine and Tryptophan Conjugation with Cyclic Imine Mannich Electrophiles**

Sijie Wang^1, †^, Lei Wang^1,†^, Marco Hadisurya^2^, Siavash Shahbazi Nia^1^, W. Andy Tao^1,2,3^, Emily C. Dykhuizen^1^, Casey J. Krusemark^1*^

^1^Department of Medicinal Chemistry and Molecular Pharmacology, Purdue Center for Cancer Research, Purdue University, West Lafayette, Indiana 47907, United States

^2^Department of Biochemistry, Purdue University, West Lafayette, Indiana 47907, United States

^3^Department of Chemistry, Purdue University, West Lafayette, Indiana 47907, United States

**Table of Contents**

1. **Supplementary Figures, Tables ………………………………………………...……….3-26**
   1. Supplementary Figures
2. **General materials and methods for the biochemical/biological experiments……..….27-32**
   1. Recombinant CBX ChD Protein Expression and Purification
   2. Fluorescence Displacement Assay to Evaluate Time-dependent potency of Imine Covalent Inhibitors to CBX ChD
   3. *In Vitro* CBX ChD Labeling with Covalent Inhibitors
   4. Cell line, Media, and Culture conditions
   5. Protein Labeling, Chemoprecipitation, and In-gel Fluorescence Labeling
   6. Proteomic Sample Preparation, Analysis, Data Processing, and Bioinformatic Analysis
   7. Cellular activity of covalent inhibitors in MLL-AF9 transformed leukemia cells
3. **General materials and methods for organic synthesis……………………...………….33-34**
   1. Materials and Methods
   2. Solid phase peptide synthesis (SPPS)
   3. Synthesis of C-terminal alkyne peptide
4. **Chemical Synthesis……………………………………………………………….……..34-49**
   1. Synthesis of model cyclic imine compounds
   2. Synthesis of cyclic imine containing CBX inhibitors
   3. Synthesis of DNA conjugates
5. **Reactions of cyclic imines ………………….………………………………………..……...50**
   1. Second order rate constant determination
   2. Selectivity of cyclic imines to other amino acids
6. **Stability and reversibility of cyclic imines with tyrosine and cysteine…………...…….....50**
   1. Stability of cyclic imines in different conditions
   2. Stability of Mannich product in different conditions
   3. Competition reaction of glutathione and acetyl tyrosine methyl amide with compound **1**
   4. Time-dependent evaluation of thiol effect on tyrosine labeling with compound **1**
   5. Competition assay evaluating glutathione on ligand-directed tyrosine labeling on protein
7. **Covalent labeling of cyclic imine ………………………………………………………50-51**
   1. Covalent labeling of cyclic imine with angiotensin II
   2. Covalent labeling of cyclic imine with CBX8 ChD
   3. Covalent labeling of cyclic imine containing ligands to CBX8 ChD
   4. Time-dependent fluorescence displacement assay
8. **Appendix 1: NMR spectra…………………………………………………………...….52-66**
9. **Appendix 2: LC-MS……………………………………………………………………..67-91**
10. **Supplementary References…………………………………………………………………92**

**Figure S1:** Representative HPLC analysis for second order rate constants. Reaction of imine **1** (5 mM) with (A) N-Ac-Tyr-NHMe (100 mM) or (B) indole-3-butyrate (100 mM) at indicated time points**.**

**Figure S2:** Second order rate constant determination. Reaction of (A) imine **1** (5 mM) with N-Ac-Tyr-NHMe (100 mM) at 37 °C or (B) at R.T. (C) imine **2** (5 mM) with N-Ac-Tyr-NHMe (100 mM) at 37 °C (D) imine **1** (5 mM) with indole-3-butyrate (100 mM) at 37 °C (E) imine **1** (10 mM) with 1 mM 1*H*-pyrrole-1-propionate (F) iminium **5** (5 mM) with N-Ac-Tyr-NHMe (100 mM) at 37 °C (G) imine **4** (5 mM) with N-Ac-Tyr-NHMe (100 mM) at 37 °C. **(H)** imine **11** (5 mM) with N-Ac-Tyr-NHMe (100 mM) at 37 °C. (I) imine **13** (5 mM) with N-Ac-Tyr-NHMe (100 mM) at 37 °C.

**Figure S3:** Time dependent hydrolysis of imine **2** in phosphate buffered D_2_O monitored by NMR.  **(A)** at pD 6.0 or **(B)** at pD 7.2. Diagnostic proton resonances are indicated for the imine proton with the red asterisk and for the glyoxylic acid aldehyde proton with the green asterisk.

**Figure S4.** Testing of additional amino acid functional groups for reactivity to imine **1**. Cyclic imine **1** was incubated with 100 mM of each compound in PBS (pH 7) at 37 °C for 24 hours and then analyzed by LC/MS. (A) Compounds include β-mercaptoethanol, imidazole, isopropanol, n-butylamine, propionic acid, 3-methyl indole, and N-ethyl pyrrole (pyrrole-lysine). UV LC/MS traces for (B) β-mercaptoethanol, (C) 3-methyl indole, and (D) N-ethyl pyrrole. LR-ESI-MS: imine-thiol product m/z calc. for [M+H]^+^ 231.1; found: 231.2. Imine-indole product m/z calc. for [M+H]^+^: 284.2; found: 284.2. Imine-pyrrole product m/z calc. for [M+H]^+^: 248.2; found: 248.2.

**Figure S5.** Reversibility of Thiol-Imine Addition. Cyclic imine **1** was treated with 100 mM β-mercaptoethanol to preform the thioaminal product, followed up with 1 M (10 eq) glutathione (GSH), another cysteine containing molecule, or 100 mM (1 eq) acetyl tyrosine methyl amide. Initial thioaminal product (m/z calc. for [M+H]^+^ 231.1; found: 231.2) was reversed by the glutathione or acetyl tyrosine methyl amide to form the new products (imine-glutathione product m/z calc. for [M+H]^+^ 460.2; found: 460.3; imine-tyrosine product m/z calc. for [M+H]^+^ 389.2; found: 389.3). LC/MS analysis showed no remaining cysteine conjugate.

**Figure S6.** Time-dependent evaluation of thiols on tyrosine labeling with imine **1** in the presence of thiols. Cyclic imine **1** (5 mM) was incubated with 100 mM acetyl tyrosine methyl amide and 5 mM GSH (in the presence of 5 mM TCEP). Reaction yield was quantified based on UV integrations of Mannich product at 4h, 9h, 17h, 24h, 32h. Imine consumption rate was quantified based on the UV integration of Mannich product at 4h, 9h, 17h, 24h, 32h.

DMSO

DMSO

DMSO

DMSO

Mannich Product

Mannich Product

Mannich Product

Tyr

Tyr

Tyr

| Solution | PBS | 1%TFA | 1%NaOH |
| --- | --- | --- | --- |
| Product Peak Integration Relative to PBS at 24 hours | 100% | 92% | 90% |

**Figure S7.** Stability of Tyr Mannich product. After 24 hours of test reactions in PBS (pH 7) at 37 °C, reaction mixture was further incubated with 1% TFA or 1% NaOH or PBS buffer for 96 hours. Mannich product was quantitated by HPLC UV peak integration to evaluate the stability of the product in acidic/basic/neutral conditions.

**Figure S8.** Extended time course labeling of eDHFR L28Y. Panels show deconvoluted mass spectra from labeling at the indicated times for 10 μM protein and 10 μM **TMP-Im**.

**Figure S9.** MS/MS spectra of labeled peptides. Proteins labeled overnight were trypsin digested and analyzed by LC-MS/MS at the Purdue Proteomics Facility with an Orbitrap Fusion Lumos Tribrid mass spectrometer. Targeted tyrosine residue is indicated in red. A) Fragmentation spectrum of **TMP-Im**-labeled peptide from eDHFR L28Y. B) Fragmentation spectrum of **P1b**-labeled peptide from CBX8. Spectrum was split to show m/z 0-1500 and m/z 1500-3000 in separate panels.

**C**

**Figure S10.** Ligand directed labeling in the presence of thiols. A) **TMP-Im** (10 μM) was incubated with eDHFR L28Y (10 μM) overnight at RT in the absence (left) or presence of 5 mM GSH with 5 mM TCEP. B) **TMP-Im** (10 μM) was incubated with eDHFR L28C (10 μM) overnight in the absence (left and center) or presence of 5 mM GSH with 5 mM TCEP. For the center panel, after the labeling reaction, GSH (5 mM) was added and allowed to incubated overnight. C) CBX8 ChD (10 μM) was reacted with **P1b** (10 μM) in the presence or absence of 10 mM GSH with 10 mM TCEP.

**Figure S11.** Covalent labeling of CBX8 ChD with cyclic imine **1**. Cyclic imine 1 (50 mM) was incubated with 50 μM CBX8 ChD in PBS (pH=7, 0.02% Tween20) buffer at R.T. for 16 hours overnight. Labeled protein was precipitated using cold MeOH:CHCl_3_ at the ratio of MeOH:CHCl_3_:Protein = 3:0.5:1, followed with two methanol washes. Protein pellet was air dried after the last wash resuspensed in water containing 0.1% formic acid and 100 mM ammonium acetate. Protein labeling was characterized by LC/MS (Unmodified CBX8 control is indicated in red, and CBX8 labeled with cyclic imine is indicated in green).

**Figure S12.** Evaluation of covalent conjugation yield by LC/MS UV integration of inhibitors labeling CBX8 ChD at R.T. or 37 °C with overnight (24h) incubation. A) **P1a** at R.T. B) **P1a** at 37 °C**.** C) **P1b** at R.T. D) **P1b** at 37 °C.

**Figure S13.** Mass spectrometry (ESI-LC/MS) of intact CBX8 chromodomain after labeling with inhibitors **P1a** and **P1b**. Masses of coresponding to labeled proteins are labeled in rectangles.

**Figure S14.** Evaluation of covalent conjugation yield by LC/MS UV integration of inhibitors labeling CBX8 ChD at 37 °C with overnight (24h) incubation or *1.5 hours for inhibitor **P2**. A) **P1c** B) **P1d C) P1e** D) **P1f** E) **P2** F) **P3a** G) **P3b**

**A**

**B**

**Figure S15.** Validation of cyclic imine inhibitor stability. A) Cyclic imine inhibitor TMP-Im was incubated 24 hours in PBS. After which, the sample was split, and one portion was reduced by cyanoborohydride. UV traces for LC/MS run as shown. low resolution mass spectrum. LR-ESI-MS: m/z calc. for **TMP-Im** [M+H]^+^: 429.2, found: 429.3; m/z calc. for **TMP-Im_CNBH_3_** [M+H]^+^: 431.2, found: 431.5. B) Imine inhibitor **P1b** was formed by treatment of **P1bL** with sodium periodate in PBS, and the sample allowed to incubate for 24 hours. After incubation, the sample was split, and one portion was further reduced with sodium cyanoborohydride. UV traces at 215 nm are shown for HPLC chromatograms. Separate LC/MS analysis confirmed peak identity. low resolution mass spectrum. LR-ESI-MS: m/z calc. for **P1bL** [M+H]^+^: 924.6, found: 924.9; m/z calc. for **P1b** [M+H]^+^: 875.5, found: 875.8; m/z calc. for **P1b_NaCNBH_3_** [M+H]^+^: 877.6, found: 878.2.

**Figure S16.** LC/MS analysis of inhibitor **P2**. Cyclic imine inhibitor **P2** was formed by treatment of **P2L** with sodium periodate and the crude sample was allowed to incubate overnight in PBS for 16 hours. After incubation, the sample was split, and one portion was treated with sodium cyanoborohydride. (**A**) Overlaid UV traces LC/MS chromatograms at 215 nm shown. (**B**) Spectrum of peak 30 minutes post oxidation. (**C**) Spectrum of peak after overnight incubation in PBS. (**D**) Spectrum of peak after NaCNBH_3_ treatment. LR-ESI-MS: m/z calc. for **P2** [M+H+H_2_O]^+^.: 881.5, found: 881.4; m/z calc. for **hydrolyzed P2** [M+H]^+^: 825.5, not observed; m/z calc. for **P2_NaCNBH_3_** [M+H]^+^: 877.6, not observed.

**Figure S17.** Stability of cyclic imine inhibitors to serum-containing cell culture media. Cyclic imine inhibitors A) **TMP-Im** and B) **P1b** were incubated at 20 μM concentration in 100 μl of Dulbecco’s Modified Eagle Medium (DMEM) (no phenol red) containing 15 % fetal bovine serum (FBS) for 24 hours at 37 ^o^C. The protein fraction was then precipitated with addition of 2 volumes of 1:1 EtOH:acetonitrile and overnight incubation at -20 ^o^C. Samples were centrifuged 16,000 g for 30 minutes. Supernatants were filtered through a 0.5 micro spin filter and evaporated with a speed vacuum. The compounds were resuspended in PBS and analzyed by LC/MS. Comparison to standards (red) showed no degradation or loss to the protein fraction of treated samples (blue). Overlaid spectra of UV at 215 nm are shown and are representative of 3 replicates.

**Figure S18.** HPLC trances of CBX8 Y39F incubated with covalent inhibitors **P1b/P1c/P3a/P2** at 37 ^o^C for 24 hours.

**A**

**Figure S19.** Characterization of Mannich reactions with an on-DNA cyclic imine. (**A**) Reaction scheme for DNA-imine modification. (**B-D**) Deconvoluted mass spectra of reaction starting material (imine) (**B**), hydroxyindole product (**C**), and tyrosine product (**D**). Cyclic imine low resolution mass spectrum. Expected mass: 6542; Mass found: 6541. Hydroxyindole product expected mass: 6732; Mass found: 6733. Tyrosine product expected mass: 6777; Mass found: 6778.

**E**

| **IC_50_ (**μ**M)** | **0h** | **1h** | **2h** | **4h** | **8h** | **16h** | **20h** |
| --- | --- | --- | --- | --- | --- | --- | --- |
| **P1a** | 12.1 | 9.3 | 10.5 | 10.5 | 5.3 | 3.5 | 2.1 |
| **P1b** | 7.4 | 6.2 | 6.3 | 6.4 | 3.6 | 2.3 | 1.5 |
| **SW2_110A** | 7.9 | 7.0 | 6.7 | 7.9 | 5.3 | 6.0 | 6.4 |

**Figure S20.** Time dependent FP assays for CBX8 ChD with covalent inhibitors **P1a** and **P1b**. FP displacement assays of CBX8 ChD with (A) **P1a**, (B) **P1b**, and (C) control non-covalent compound **SW2_110A** were conducted at indicated time points using fluorescein-label ligand (D) **SW2_113** as the probe (see ref ^1^). (E) Observed IC_50­_ values as a function of time. Assays were conducted using **SW2_113** (100 nM, K_d_ to CBX8 ChD = 0.8 μM), CBX8 ChD (2 μM) in PBS (pH 7), 0.02% Tween 20.

**A**

**B**

FP probe: **SW2_123**

**Figure S21.** Evaluation of CBX8 ChD long term stability using fluorescence polarization. To evaluate the stability of CBX8 ChD over 20 hours of time-dependent fluorescence polarization assays, the binding affinity (K_d_) of the FP probe (B) (**SW2_123**, see ref ^1^) was quantified at 0 h and 20 h, using a direct FP binding assay (A). The result showed comparable K_d_ values of the probe **SW2_123** at 0 h (~0.7 ± 0.1 μM) and 20 h (~1 ± 0.1 μM), indicating CBX8 ChD protein stability within 20 hours during the time course FP assay.

**Figure S22:** Denaturing washes for chemoprecipitations from HEK293T cell lysates using **SW2-110A-B** and **P1b-B**. Immunoblot analysis of enriched proteins as conducted in Figure 4C. Wash buffer used was either **(A)** 0.2% SDS in PBS or **(B)** 0.5% SDS, 1M urea in PBS. Lane 1: 3% input. Lane 2: Streptavidin beads only with no ligand. Lane 3: **SW2-110A-B** (non-covalent) pulldown. Lane 4: **P1b-B** (covalent) pulldown.

**
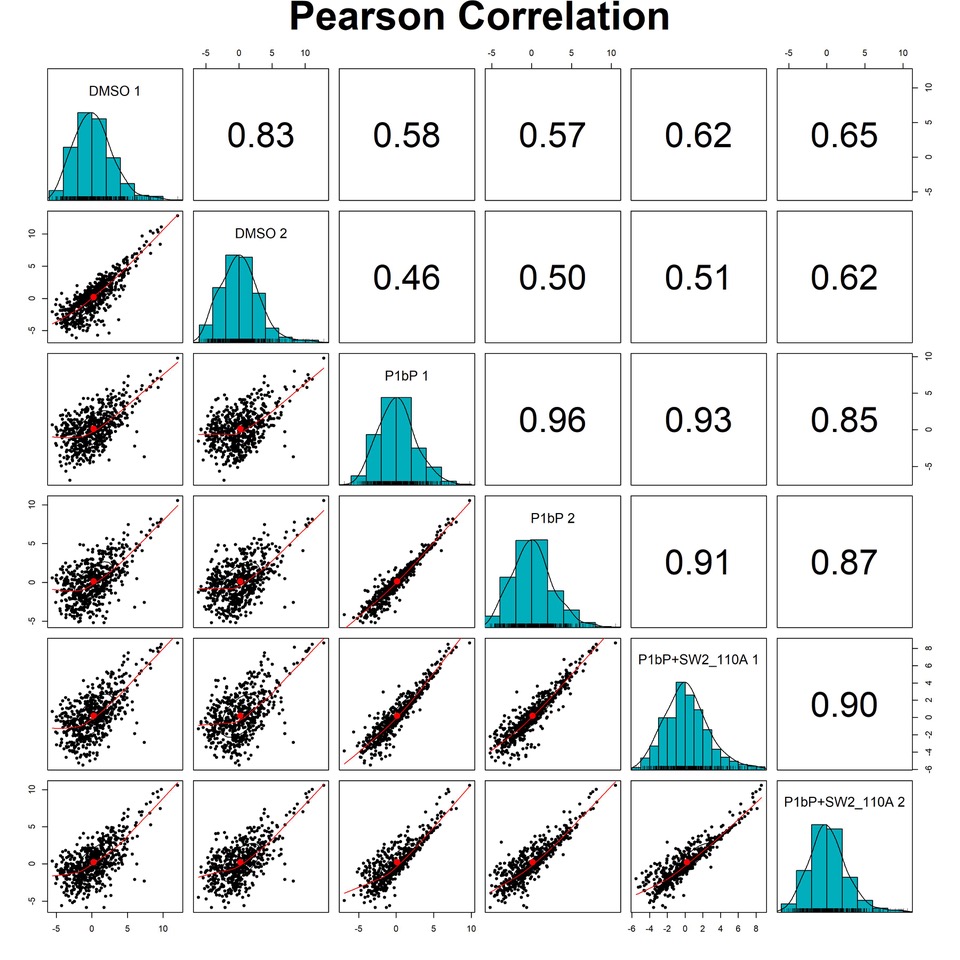
**

**Figure S23:** Pearson Correlation analysis of biological replicates for proteomic labeling, purification, and mass spectrometry analysis.

**

**Figure S24**. Cellular activity of covalent, cyclic imine inhibitors to CBX8. qRT-PCR analysis of *HOXA9* gene expression in THP1 cells after 48 h of compound treatment at the indicated dose. N = 3. Error bars represent s.d. Statistical significance determined by Student’s T test. * = p <0.05, ** = p < 0.01, *** = p < 0.001, **** = p < 0.0001.

**2. General materials and methods for the biochemical/biological experiments**

**2.1 Recombinant Protein Expression and Purification.**  *E. coli* DHFR genes were synthesized by GenScript with optimized codons and subcloned into pET-28b(+) expression constructs. CBX chromodomain constructs (addgene plasmid #25158 (CBX2), and #62514 (CBX8, provided by Cheryl Arrowsmith)^2^. Plasmids transformed into chemically competent BL21 CodonPlus RIL *E. coli* cells (Stratagene, La Jolla, CA) or Rosetta 2 Competent Cells (EMD Millipore). All proteins were expressed and purified by 6His–tags. Bacterial growth was completed at 37 ℃ in LB media to OD600 = 1.5, followed by reducing the temperature to 16 ℃ over 30–60 min and induced with 1 mM IPTG for 16 h. Cells were collected by centrifugation at 6000 rpm for 20 min and resuspended in binding buffer (20 mM Tris, pH 8, 150 mM NaCl, 0.01% Tween 20, 20 mM imidazole) with 1.0 mM PMSF. Bacteria pellets were stored at –80 ℃ until needed. Pellets were thawed on ice for 10 min in binding buffer supplemented with 100 μg/mL lysozyme, 1 mg/mL CHAPS and 1 mM PMSF. The solution was incubated at 4 ℃ for 30 min with shaking. Cells were subsequently lysed by sonication (2x: 15W for 30s on, 30 s off, followed by 1x: 20W for 1 min). The solubilized fraction was collected by centrifugation at 15000 rpm for 40 min at 4 ℃. Meanwhile, Ni-NTA Agarose resin (QIAGEN, Venlo, Netherlands) was washed with H_2_O and equilibrated with binding buffer. The soluble fraction was incubated with the prewashed Ni-NTA agarose resin at 4 ℃ for 2 h. The resin was then washed 3X with purification buffer (20 mM Tris, pH 8, 150 mM NaCl, 0.01% Tween 20, 1 mM PMSF). Proteins were eluted with 0.5 M imidazole in purification buffer. The elution was diluted with 30% glycerol, flash frozen, and stored at –80 ℃ until needed. Protein purity was assessed by SDS-PAGE and concentration was determined by the Pierce 660 kit (Thermo Scientific). Protein was dialyzed into phosphate buffer (100 mM Na_2_HPO_4_, 150 mM NaCl, 0.02% Tween 20, pH 7) for biochemical assays.

**Preparation of HisCBX8 Y39F mutant**

The Y39F CBX8 mutant was prepared by Biomatik in the pET-28a(+) vector (Bacterial, #VGG043) using cloning site 5': NcoI, cloning site 3': HindIII, 5' Restriction site: NcoI, 3' Restriction site: HindIII. The final insert sequence is below.

Insert sequence (235bp): CCATGGGACATCATCATCATCATCACAGCAGCGGCAGAGAAAACTTGTATTTCCAGGGCGAGCGGGTGTTCGCGGCCGAAGCCCTCCTGAAGCGGCGCATACGGAAAGGACGCATGGAATACCTCGTGAAATGGAAGGGATGGTCGCAGAAGTTTAGCACATGGGAACCGGAGGAAAACATCCTGGATGCTCGCTTGCTCGCAGCCTTTGAGGAAAGGGAATGATGACGAAGCTT

**Sequence of eDHFR genes and proteins**

The recombinant gene and protein sequence of the parent eDHFR proteinare shown below with the L28 highlighted in red, which was changed for L28Y, L28W, and L28C mutants.

**eDHFR_6his_WT: gene**

ATGATTTCACTAATAGCGGCATTAGCTGTAGACCGTGTGATCGGCATGGAAAACGCTATGCCGTGGAACCTGCCGGCAGACCTCGCGTGGTTCAAACGTAATACTCTGAATAAACCGGTGATCATGGGTCGTCATACCTGGGAGAGCATTGGCAGACCTCTGCCGGGTCGCAAGAACATTATCTTGTCTTCGCAACCGGGTACGGATGACCGCGTCACCTGGGTTAAAAGCGTTGATGAGGCGATTGCGGCATGTGGTGACGTGCCGGAAATCATGGTTATCGGCGGCGGCCGCGTGTACGAGCAGTTCCTGCCAAAGGCGCAGAAGCTGTATCTGACCCATATTGATGCGGAAGTTGAAGGTGATACCCACTTTCCGGATTATGAGCCGGACGACTGGGAGTCCGTCTTTTCCGAATTTCATGATGCTGACGCCCAAAACAGCCATAGCTACTGCTTCGAGATCTTGGAACGTCGTCATCACCACCACCACCAC

**eDHFR_6his_WT: protein**

**average MW: 18822.22 Da**

MISLIAALAVDRVIGMENAMPWNLPADLAWFKRNTLNKPVIMGRHTWESIGRPLPGRKNIILSSQPGTDDRVTWVKSVDEAIAACGDVPEIMVIGGGRVYEQFLPKAQKLYLTHIDAEVEGDTHFPDYEPDDWESVFSEFHDADAQNSHSYCFEILERRHHHHHH

**2.2 Fluorescence Displacement Assay to Evaluate Time-dependent potency of Imine Covalent Inhibitors to CBX ChD**. Fluorescence polarization (FP) was measured by titration of CBX ChDs (CBX8 ChD/CBX8 ChD Y39F/CBX2 ChD) to a fluoresceine-labelled probe as previously reported ^1^. Fluorescence displacement assays were performed in black 384-well plates with optical bottoms. Buffer used in FP assays consists of 100 mM Na_2_HPO_4_, 150 mM NaCl, 0.02% Tween 20. The FAM-labeled probe was kept constant at 100 nM with 2 µM CBX8 ChD, 2 µM CBX8 ChD Y39F or 2 µM CBX2 ChD, concentrations selected based on the reported relative affinity of the CBX ChD protein for the FAM probes. Two-fold dilutions of covalent inhibitors were used, starting with 100 µM as highest peptide concentration to 0.049 µM as the lowest. Four replicates were tested at each concentration. Fluorescence anisotropy was read at different time points. Raw data were analyzed using GraphPad Prism 7 following a “one site-Fit logIC_50_” competition model with any outliers (95 % confidence interval) being excluded.

**2.3 *In Vitro* CBX ChD Labeling with Covalent Inhibitors.** 10 μM purified CBX ChD was incubated with imine covalent inhibitors (10 μM) in PBS buffer (pH 7) at 37 °C or R.T. Protein labeling samples were precipitated using MeOH and CHCl_3_ at the volume ratio of MeOH:CHCl_3_:sample=3:0.5:1, washed twice using cold MeOH, and air dried. Dried protein pellets were resuspended in aqueous buffer (0.1% formic acid, 300 mM ammonium acetate).

**UV-Based Quantification of Protein Labeling Yield by LC–UV-MS**

Precipitated samples were analyzed on a C4 reversed-phase column (e.g., 2.1 × 150 mm, 3–5 µm particle size) using a linear gradient of solvent A (0.1% formic acid in water) and solvent B (0.1% formic acid in acetonitrile). Elution was monitored by UV absorbance at 215 nm, and mass spectra were acquired in positive ion mode to confirm the identities of unlabeled and labeled protein species.

**-Peak Assignment**

Unlabeled and labeled protein species were assigned based on their experimentally observed intact masses determined by LC–MS. UV chromatographic peaks corresponding to each species were identified by extracting MS spectra across the elution window and confirming the presence of the expected deconvoluted masses for unlabeled and labeled proteins, respectively.

**-UV-Based Quantification**

Protein labeling yield was quantified using UV absorbance signals under the assumption that the unlabeled and labeled proteins possess comparable molar extinction coefficients at the selected detection wavelength. This assumption is valid because the chemical modification introduces a small mass change (<1kDa) relative to the protein backbone and does not significantly alter the chromophore content responsible for UV absorbance at 215 nm.

For partially overlapping chromatographic peaks, quantification was performed using peak deconvolution and integration of extracted UV traces. Specifically, the total UV signal across the elution window containing both species was fit using a sum of two Gaussian (or exponentially modified Gaussian) functions, with peak centers constrained by retention times determined from MS-confirmed species assignments. Integrated peak areas corresponding to the unlabeled and labeled protein were obtained from the fitted curves.

**Calculation of Labeling Yield**

Labeling yield was calculated as:

$$\text{Labeling yield (\%)}=\frac{A_{\text{labeled}}}{A_{\text{labeled}}+A_{\text{unlabeled}}}\times100$$

where $A_{\text{labeled}}$and $A_{\text{unlabeled}}$represent the integrated UV peak areas of the labeled and unlabeled protein species, respectively.

**2.4 Cell Culture** **Cell line, Media and Culture Conditions** HEK293T cells were cultured in Dubecco’s Modified Essential Media (DMEM), 10% fetal bovine serum (FBS, JR Scientific), 1% glutagro (Corning), 1% penicillin/streptomycin (Corning), 1% sodium pyruvate (Corning). Human THP1 cells were cultured in RPMI (Gibco), 10% FBS (J R Scientific), 1% Sodium pyruvate (Invitrogen), 1% Pen/Strep (Invitrogen), 1% Glutamax (Thermo Scientific), 0.1% 2-mercaptoethanol.

**2.5 Protein Labeling, Chemoprecipitation and In-gel Fluorescence Labeling**

**Preparation of Lysates for Chemoprecipitation and MS-based Proteomic Analysis** HEK293T cells were grown to confluency in 15 cm cell culture dishes and washed with PBS. ~100 million cells were suspended with 4 mL Buffer A (25mM HEPES, 5mM KCl, 25mM MgCl_2_, 0.05mM EDTA, 10% glycerol, 0.1% NP-40, plus protease inhibitors) using a cell lifter. The outer membranes were lysed on ice for 15 minutes. The nuclei were pelleted and re-suspended in 4 mL lysis buffer (20 mM HEPES pH 8, 150 mM NaCl, 0.3% NP-40, protease inhibitor). Benzonase (300U) was added and the lysate was incubated at 37 °C for 10 minutes with agitation at 500 rpm and then incubated at room temperature for 10 minutes with agitation at 500 rpm. The samples were centrifuged at 20,000 rpm at 4 °C for 30 min and lysates were transferred to separate tubes. Lysates were diluted with PBS at the ratio of lysate:PBS (1:2). Streptavidin agarose beads (500 μL) (ThermoFisher, #20353) were incubated with lysate at 4 ^o^C for 2 hours to remove endogenously biotinylated proteins from diluted lysates. Samples were centrifuged at 2,500 g for 10 mins. Lysates were then transferred to new separate tubes. The protein concentration was determined using the BCA assay (Bio-Rad) following the manufacturer’s protocol.

**Labeling Endogenous Proteins from Lysates using P1b and Competition** To prepare covalent inhibitors for protein labeling, **P1bPL** was treated with sodium periodate (NaIO_4_) in PBS (pH 7), at R.T. for 30 mins. Cyclized imine inhibitor **P1bP**, along with non-covalent inhibitor **SW2_110A**, were then immediately used for pulldown. For each pulldown, ~500 μg nuclear lysate was added to inhibitors (1 μM final compound concentration) or control DMSO. For competitions, 20 eq non-covalent inhibitor with amide termini (**SW2_110A_amide**) were added. The protein labeling mixture using **P1bP** was shaken at 37 °C for 12 h overnight (same for competition group).

**Click Chemistry Ligation** Labeled lysates was incubated with pre-mixed click reagents, containing 100 μM biotin-PEG4-N_3_ (for pulldown and proteomics) or 100 μM TAMRA-N_3_ (for in-gel fluorescence), 0.1 mM CuSO_4_, 0.5 mM BTTAA, 5 mM sodium ascorbate, 5 mM aminoguanidine. The reaction was quenched with addition of EDTA (5 mM final concentration). Protein was precipitated by adding MeOH/CHCl_3_ at a ratio of 3:0.5, relative to the sample volume. The precipitate was washed twice with cold MeOH, and air-dried for 5 min.

**SDS-PAGE and In-gel Fluorescence Labeling** 4× Bolt LDS Sample Buffer (Invitrogen) with 40% β-mercaptoethanol (AMRESCO LLC, Solon, OH) was added to samples (10 μg) which were boiled at 95 °C for 5 min. The protein samples were loaded onto 12% (w/v) acrylamide Tris-HCl gels using Tris/glycine/SDS running buffer (0.25 M Tris, 0.2 M glycine, 0.1% (w/v) SDS). Gels were imaged on a Typhoon imager (GE Healthcare; λEx 532 nm, λEm 575 nm for TAMRA fluorophore; λ Ex 635 nm, λ Em 575 nm for protein MW markers (PageRuler™ Plus Prestained Protein Ladder, 10 to 250 kDa, # 26619, ThermoFisher). For protein loading quantification, gels were stained with Coomassie Brilliant Blue R-250 Stain solution, washed, and imaged on an CanoScan LiDE 220 (Canon).

**Biotin Affinity Enrichment for Immunoblot Analysis** After protein samples were labeled, ligated to biotin-PEG4-N_3_, and precipitated, protein pellets after precipitation were resuspended with 600 μL PBS for each sample. Meanwhile, streptavidin M-270 Dynabeads (Solulink, San Diego, CA) was washed three times with binding buffer (lysis buffer:PBS=1:2). 30 μL beads were then added to each resuspended sample and mixtures were rotated at R.T. for 1 hour. After 1 hour incubation, lysate was then depleted, and buffers with different stringencies (non-denaturing/stringent/denaturing) were used for washing (nondenaturing: 50mM Tris, 150mM NaCl, 0.3% NP-40; stringent buffer: 50mM Tris, 150mM NaCl, 1% NP-40, 0.1% DOC; denaturing buffers: 0.2-1% SDS in PBS or 6M urea in PBS). The beads were washed with different buffers for 3 times, with 5 minutes tumbling each time at R.T. For every wash, the supernatant was removed from the magnetic beads by a quick centrifugation (600 g, 30s) followed up with a magnetic rack separator. For Western Blot, the bound proteins were eluted from the beads with 1× Bolt LDS Sample Buffer (Invitrogen) with 10% β-mercaptoethanol (AMRESCO LLC, Solon, OH). The samples, along with 10% input samples were heated at 95 °C for 5 min and loaded onto a 4–12% gradient gel (Invitrogen) for immunoblotting analysis of the proteins of interest. ImageJ was employed to quantitate the protein bands. For mass spectrometry, the beads were resuspended in 10 μL PBS and the bound proteins were stored at – 80 °C.

**2.6 Proteomic Sample Preparation, Analysis, Data Processing and Bioinformatic Analysis**

**Biotin Affinity Enrichment using Denaturing Wash** After protein samples were labeled, ligated to biotin-PEG4-N_3_, and precipitated, protein pellets after precipitation were resuspended with 600 μL PBS for each sample. Meanwhile, streptavidin M-270 Dynabeads (Solulink, San Diego, CA) was washed three times with binding buffer (lysis buffer:PBS=1:2). 30 μL Beads was then added to each resuspended sample and mixtures were rotated at R.T. for 1 hour. After 1 hour incubation, lysate was then depleted, and denaturing buffer was used (0.2-1% SDS in PBS) for washing. The beads were washed with 0.2% SDS in PBS for 3 times (5 mins for each time) at R.T., followed up by a quick gentle wash with 1% SDS in PBS. Then beads were washed three times with PBS, and three times with water to remove potential leftover SDS. Beads were resuspended in 10 μL PBS after the final wash, and immediately stored in -20 °C for next step LC-MS.

**LC-MS Sample Preparation** The phase-transfer surfactant (PTS) buffer containing 12 mM sodium deoxycholate, 12 mM sodium lauroyl sarcosinate, 10 mM TCEP, and 40 mM chloroacetamide (CAA) in 50 mM Tris·HCl, pH 8.5 was added to the magnetic beads. The proteins were incubated for 10 min at 95°C and digested on beads with Lys-C (Wako) at 1:100 (wt/wt) enzyme-to-protein ratio for 5 hr at 37°C. Furthermore, trypsin was added to a final 1:50 (wt/wt) enzyme-to-protein ratio for overnight digestion at 37 °C. The supernatant containing the digested proteins was separated from the magnetic beads with the help of a magnetic rack separator and processed further. Then, a final concentration of 1% trifluoroacetic acid (TFA) was added to acidify the samples. An ethyl acetate solution was added at a 1:1 ratio to the samples. The solution was vortexed for 2 min and then centrifuged at 20,000 x g for 3 min to separate the aqueous and organic phases. The top layer (the organic phases) was removed, and the aqueous phase was collected, dried down in a vacuum centrifuge, and desalted using TopTip C18 tips (Glygen) according to the manufacturer’s instructions. The desalted samples were dried completely in a vacuum centrifuge.

**LC-MS Analysis** The proteomics samples were spiked with an 11-peptide Retention Time internal standard (Biognosys) to normalize the LC-MS signal between sample runs. All samples were loaded into an Easy-nLC 1000 (Thermo Fisher Scientific) and separated with a 45-cm packed column (360-µm o.d. × 75-µm i.d.) containing C18 resin (2.2 µm, 100Å; Michrom Bioresources) with a heated 30-cm column heater (Analytical Sales and Services) set to 50°C. The mobile phase buffer contained 0.1% formic acid in HPLC grade water (buffer A) with an eluting buffer containing 0.1% formic acid in 80% (vol/vol) acetonitrile (buffer B) run with a linear 60-min gradient of 10-35% buffer B at a flow rate of 300 nL/min. The HPLC was coupled with an LTQ-Orbitrap Velos Pro mass spectrometry (Thermo Fisher Scientific). The mass spectrometry was run in data-dependent mode with a full-scan MS (from m/z 350 to 1,500 with a resolution of 30,000), followed by MS/MS of the 10 most intense ions subjected to collision-induced dissociation (CID) fragmentation. CID fragmentation was performed and acquired in the linear ion trap (minimal signal threshold 1,000 counts, normalized collision energy (NCE) 30%, activation Q 0.25, activation time 10 ms, default charge state 3, isolation window 3 m/z, and dynamic exclusion 60 s).

**LC-MS Data Processing** The raw files were searched against the human Swiss-Prot database with no redundant entries, using Sequest and Byonic (Protein Metrics) search engines loaded into Proteome Discoverer 2.3 software (Thermo Fisher Scientific). MS1 precursor mass tolerance was set at 10 ppm, and MS2 fragment tolerance was set at 0.6 Da. In the processing workflow, search parameters for both search engines were performed with full trypsin/P digestion, a maximum of two missed cleavages allowed, a static modification of carbamidomethylation on cysteines (+57.0214 Da), and variable modifications of oxidation (+15.9949 Da) on methionine residues and acetylation (+42.011 Da) at N terminus of proteins. The false-discovery rates (FDR) of peptide spectrum matches (PSMs), peptides, and proteins were set at 0.01 (strict) and 0.05 (relaxed). All protein and peptide identifications were grouped, and any redundant entries were removed. Unique peptides and unique master proteins were reported. Finally, the proteomic abundance results were further normalized using the spiked 11-peptide Retention Time internal standard.

**Bioinformatics Analysis** All sample data were analyzed using the Perseus software (version 1.6.15.0)^3^. Proteome Discoverer search results extracted the normalized intensities of proteins, and log-based 2 was transformed. The abundances were categorized into their respective categories. The proteins with detected abundances of at least 12 out of 16 samples were kept. The imputation for the missing abundances was performed by assigning small random values from the normal distribution with a downshift of 1.8 SDs and a width of 0.3 SDs. All abundances for each protein were further normalized by subtracting the median of each sample run abundances from each protein abundance. Then, the limma moderated t-test was performed, and the difference in averages was calculated for each comparison. Volcano plots were created for each comparison.

**Cellular activity of covalent inhibitors in MLL-AF9 transformed leukemia THP1 cells** 1× 10^6^ THP1 cells were treated with covalent inhibitors or non-covalent inhibitor **SW2_110A** at 3-fold serial doses or control DMSO (0.1%) for 48 hours. Cells were harvested after 48 h for RNA extraction. After homogenization of THP1 cells using TRIzol reagent (Thermo Scientific), RNA was extracted from the aqueous phase in the phase separation step. RNA pellet was washed with 75% ethanol and concentrated for subsequent reverse transcription. 1 μg RNA was then converted into cDNA using Verso cDNA synthesis kit (Thermo Scientific). SYBR Green Mastermix (Thermo Scientific) was used for quantitative PCR. Primers below are used in the qPCR.

| Gene name | Forward primer (5’-3’) | Reverse primer (5’-3’) |
| --- | --- | --- |
| *HOXA9* | GGCCCAGGACCGAGATACTT | CGCTCACGGACAATCTAGTTGT |
| *B2M* | TGCTGTCTCCATGTTTGATGTATCT | TCTCTGCTCCCCACCTCTAAGT |

**3. General Materials and Methods for Organic Synthesis**

**3.1 Materials and Methods** Organic compounds as starting materials were commercially purchased and used as provided. Commercial imine compounds tested in table 1 (compound 4 (2-hydroxyquinoxaline, Thermo Scientific, CAS: 1196-57-2), compound 8 (harmaline, Chem-Impex, CAS: 304-21-2), compound 9 (3,4-dihydroisoquinoline, Enamine, CAS: 3230-65-7), compound 10 (2-methyl-1-pyrroline, Chem-Impex, CAS: 872-32-2) were used as received from commercial resources. Amine modified oligonucleotide was received from Integrated DNA Technologies (IDT). Analytical high-performance liquid chromatography (HPLC) separations were completed using an Agilent 1100 system with detection at 215 nm using a water/MeCN gradient containing 0.1% TFA. Preparative HPLC separations were completed using a Varian ProStar system with detection at 215 and 254 nm using a water/MeOH gradient containing 0.1% TFA. All low-resolution ESI-LCMS was completed using a Waters Acquity UPLC with SQD2 mass spectrometer, equipped with a C18 reverse phase column, or an Agilent LC/MSD iQ (Agilent #G6160A, single quadrupole). Eluents of MeCN and water (each with 0.1% (v/v) formic acid) were used as linear gradients (% (v/v) MeCN in water) of: 5% to 95% (0.5 to 6 min), 95% to 5% (6 to 6.5 min), and 5% (6.5 to 7.0 min). For oligonucleotide conjugates, eluents of mobile phase A (50 mM hexafluoroisopropanol (HFIP) with 15 mM diisopropylethylamine (DIEA) in 10% methanol) and mobile phase B (50 mM HFIP with 15 mM DIEA in 90% methanol) were used with a 5% A to 65% B linear gradient over 20 minutes.

Reactions were monitored by TLC on MilliporeSigma silica gel 60 Å glass plates (20 × 20 cm, 250 µm thickness). Flash column chromatography was performed using Sorbent Technologies® 70 Å silica gel (40-75 μm particle size). NMR spectra were recorded in CDCl_3_ (Acros Organics) on a 500 MHz Bruker AV NMR spectrometer at 298 K (500 MHz for 1H and 126 MHz for 13C). Chemical shifts (δ) are reported in parts per million (ppm) and multiplicities reported as: s, singlet; d, doublet; dd, doublet of doublets; t, triplet; q, quartet; m, multiplet; br., broad. Coupling constants are reported in hertz (Hz). Spectra were analyzed using MestReNova 14 software. Residual solvent signals were used as an internal standard. Reagents and solvents were used as received from commercial sources.

**3.2 Solid phase peptide synthesis (SPPS).** Peptides were prepared using traditional SPPS methods. All couplings and deprotections were monitored by ninhydrin tests. Briefly, 50 mg of Rink Amide MBHA resin was allowed to swell for 20 minutes in 1,2-dichloroethane and 20 minutes in DMF before suspension in 20% piperidine in DMF for the initial Fmoc deprotection for 30 minutes at RT. Couplings were completed using 5.0 eq. (relative to the capacity of the resin) of Fmoc-AA (or carboxylic acid), 5.0 eq. HOAt, and 5.0 eq. DIC in DMF (approximately 0.1 M) and pre-activated for 20 minutes at RT before being added to the resin. Fmoc deprotections were achieved by incubating the resin for 30 minutes at RT in 20% piperidine in DMF. Peptides were cleaved and deprotected by incubating in 95% TFA, 2.5% triisopropylsilane, and 2.5% H_2_O for 3 hours at RT. The crude peptide was collected by precipitation out of ice-cold diethyl ether and then suspended in 50% MeOH/50% H_2_O and concentrated to dryness. The residue was dissolved in DMSO and purified on a semi-prep HPLC using a H_2_O/MeOH + 0.1% TFA gradient with detection at 215 nm and 254 nm. Yield was determined by mass of the dried, purified peptide as the TFA salt relative to the equivalents as determined by the mass of resin used. Purity was confirmed to be > 95% by LC/MS.

**3.3 Synthesis of C-terminal alkyne peptide** Modified methods from previously reported procedure ^4^ were used to synthesize C-terminal alkyne peptides. Polystyrene-linked aldehyde resin (FMPB AM resin, 100 mg, 1.08 mmol/g) was added to a round bottom flask and gently stirred for 30 minutes at RT in DCM. DCM was gently evaporated and 5 mL of DMF with 5 mL of MeOH was added to the resin. To this, 10 eq. glacial AcOH was added with 10 eq. propargyl amine and 10 eq. NaCNBH3 and gently stirred under light reflux for 3 hours at 80 °C. The mixture was cooled and washed with MeOH, DCM, and DMF and re-swelled for 30 mins in 1,2-dichloroethane prior to the first acylation. To the resin, 5.0 eq. Fmoc-Ser(OtBu)-OH with 5.0 eq. DIC, 8.0 eq. HOAt in DMF was added and incubated at 37 °C overnight. The remaining synthesis, purification, and reductive amination were completed as described above. Purity was confirmed to be > 95% by HPLC.

**4. Chemical Synthesis**

**4.1 Synthesis of model cyclic imine compounds**

**Scheme S1.** Synthesis of compound **1** (iminolactam)

As previously described ^5^, to 4 mmol trans-1,2-diaminocyclohexane (456.8 mg, 4 mmol, 2 eq) in 5mL iPrOH, 2 mmol of ethyl glyoxylate (204.2 mg, 2 mmol,1 eq) in 50% toluene in 5 mL iPrOH was added dropwise with stirring at R.T. The mixture was stirred for 3 hours and concentrated for flash column purifications to afford cyclic imine **1** (126.1 mg, 0.83 mmol, 42 %). Spectra were consistent with a previous report of the compound ^5^. **^1^H NMR** (500 MHz, CDCl_3_) δ 7.72 (t, *J* = 2.9 Hz, 1H), 6.94 (s, 1H), 3.16 (td, *J* = 11.6, 3.9 Hz, 1H), 3.09 (ddt, *J* = 11.8, 8.4, 3.6 Hz, 1H), 2.36 (dq, *J* = 8.9, 3.3 Hz, 1H), 1.98 – 1.91 (m, 1H), 1.91 – 1.86 (m, 1H), 1.84 – 1.77 (m, 1H), 1.43 (ddd, *J* = 12.7, 7.7, 3.4 Hz, 3H), 1.32 (ddd, *J* = 16.6, 8.4, 3.6 Hz, 1H). ﻿**^13^C NMR** (126 MHz, CDCl3) δ 157.91, 156.27, 62.87, 53.96, 31.36, 30.93, 25.15, 23.55. **LR-ESI-MS**: m/z calc. for C8H12N2O ([M+H]^+^): 153.1, found: 153.1.

**Scheme S2.** Synthesis of compound **2** (iminolactone)

As previously described ^6^, 2-amino hexanol (1.15 g, 10 mmol, 1 eq) and triethylamine (1.518 g, 15 mmol, 1.5 eq) were combined in 20 mL CH_2_Cl_2_. 1 eq of o-nitrobenzenesulfonyl chloride (10 mmol, 2.216 g) was added at 0 °C. After 5 min stirring, saturated NaHCO_3_ was poured into the reaction mixture, and extracted with CH_2_Cl_2_. The organic phase was washed with 1N HCl and brine, dried using MgSO_4_, filtered and concentrated under reduced pressure to give the nosyl sulfonamide alcohol, which is directly used for the next reaction. Nosyl sulfonamide alcohol (1.5 g, 5 mmol, 1 eq) was added to 1.2 eq pyridine (474.6 mg, 6 mmol), and 1.1eq bromoacetyl bromide (1.11g, 5.5 mmol) in 30 mL DCM for 15 mins at 0 °C. After 15 mins, saturated NaHCO_3_ solution was poured into the reaction, and extracted with DCM. Extraction was washed again with 1N HCl and brine, dried over MgSO_4_, filtered, and concentrated under pressure. Silica gel (10 g silica) column chromatography was used to purify the product. A gradient of 25-50% ethyl acetate in hexane was used to elute the bromoacetylated product (1.55 g, 3.7 mmol, 74% yield). Lastly, the bromoacetylated product (0.84g, 2 mmols, 1eq) was added to 2 eq of cesium carbamate (1.3 g, 4 mmol) and 0.2 eq of sodium iodide (60 mg, 0.4 mmol) in 20 mL MeCN. The reaction was refluxed at 80 °C for 1h. After 1h, the reaction mixture was diluted with EtOAc, and filtered using a pad of celite, and purified using silica column. 100 % ethyl acetate was used to elute and afford the final product compound **2** (ester imine) (0.167 g, 1.1 mmol, 55 % yield). R_f_ = 0.75 in ethyl acetate **^1^H NMR** (500 MHz, CDCl_3_) δ 7.84 (dd, *J* = 3.4, 1.4 Hz, 1H), 4.05 – 3.96 (m, 1H), 3.27 – 3.17 (m, 1H), 2.46 (dtd, *J* = 11.0, 3.9, 1.9 Hz, 1H), 2.16 (dqd, *J* = 11.1, 3.1, 1.6 Hz, 1H), 1.88 (ddq, *J* = 13.2, 4.2, 2.1 Hz, 2H), 1.65 – 1.53 (m, 1H), 1.49 – 1.26 (m, 3H). ﻿**^13^C NMR** (126 MHz, CDCl3) δ 155.48, 152.71, 80.56, 61.06, 31.13, 30.50, 24.61, 23.41. **LR-ESI-MS**: m/z calc. for C8H11NO2 ([M+H]^+^): 154.1, found: 154.2.

**Scheme S3.** Synthesis of compound **3** (aromatic iminolactone)

As with the synthesis of compound **1**, 4.6 mmol 2-amino phenol (505 mg, 1 eq) was mixed with 2 eq ethyl glyoxylate (941mg, 9.2 mmol) in 25 mL dry THF, with additions of 1.5 eq K_2_CO_3_ (954 mg, 6.9 mmol), and 1g 3A molecular sieve. The reaction was refluxed at 80 °C overnight. The reaction mixture was concentrated for column purification with a gradient of 0-10% ethyl acetate in hexanes to afford **3** (aromatic ester imine, 186.2 mg, 1.28 mmol, 28%). **^1^H NMR** (500 MHz, CDCl_3_) δ 8.10 (s, 1H), 7.79 (dd, *J* = 8.0, 1.6 Hz, 1H), 7.55 (ddd, *J* = 8.6, 7.5, 1.6 Hz, 1H), 7.38 (td, *J* = 7.7, 1.3 Hz, 1H), 7.31 (dd, *J* = 8.3, 1.3 Hz, 1H). ﻿**^13^C NMR** (126 MHz, CDCl3) δ 152.24, 146.24, 146.13, 132.00, 131.09, 129.53, 125.59, 116.72. **LR-ESI-MS**: m/z calc. for C8H5NO2 ([M+H]^+^): 148.03, found: 148.06.

**Scheme S4.** Synthesis of compound **4** (cyclic iminium lactam)

To a stirred solution of tert-butyl (2-(phenylamino)ethyl)carbamate (300 mg, 1.27 mmol) in isopropanol, N,N-diisopropylethylamine (DIEA) (575 mg, 4.44 mmol, 3.5 equiv) was added at room temperature. The reaction mixture was stirred for 15 minutes. Then, Boc-Ser(tBu)-OH (430 mg, 1.65 mmol, 1.3 equiv) was added, and the solution was cooled to 0 °C. After cooling, propylphosphonic anhydride (T3P) (50% in EtOAc) (605 mg, 1.90 mmol, 1.5 equiv) was added dropwise. The reaction mixture was allowed to warm to room temperature and stirred overnight. The reaction mixture was concentrated under reduced pressure, then washed with 1 M HCl and extracted with ethyl acetate. The combined organic layers were dried, concentrated, and purified by silica gel column chromatography to afford the intermediate. (600mg, 98% yield). The Boc and tBu protecting groups were removed by treatment with trifluoroacetic acid (TFA). The resulting crude product was subjected to an oxidation reaction using sodium periodate (NaIO₄) in PBS buffer (pH ~7). The reaction mixture was extracted with ethyl acetate, and the organic layer was dried, concentrated, and purified by silica gel column chromatography to afford compound 4. **1H NMR** (500 MHz, CDCl_3_) δ 8.00 (s, 1H), 7.43 (dd, J = 8.4, 7.3 Hz, 2H), 7.36 – 7.28 (m, 3H), 3.98 (dd, J = 7.0, 4.3 Hz, 2H), 3.70 (t, J = 5.8 Hz, 2H). **13C NMR** (126 MHz, CDCl_3_) δ 159.35, 157.18, 140.68, 129.38, 127.53, 124.70, 48.87, 39.39. **LR-ESI-MS**: m/z calc. for C10H17N2O ([M]^+^): 175.08, found: 175.1.

**Scheme S5.** Synthesis of compound **5** (cyclic iminium lactam)

To 4 mmol trans-1,2-diaminocyclohexane (569.2 mg, 4 mmol, 1 eq) in 5 mL iPrOH, 3 mmol of ethyl glyoxylate (306.8 mg, 0.75 eq) in 50 % toluene in 5 mL iPrOH was added dropwise with stirring at R.T. The mixture was stirred for overnight and concentrated for silica gel column purification to afford compound 4 (cyclic iminium). Product was eluted using 100% ethyl acetate, followed up by 10% MeOH in DCM, to afford compound **4** (489.6 mg, 2.78 mmol, 90 % yield). **^1^H NMR** (500 MHz, Acetic Acid) δ 7.67 (s, 1H), 3.63 (s, 5H), 3.12 (d, *J* = 13.0 Hz, 1H), 3.08 (s, 7H), 3.06 (s, 1H), 3.05 – 2.96 (m, 1H), 2.46 (d, *J* = 9.7 Hz, 3H), 2.36 (s, 4H), 2.10 (t, *J* = 2.3 Hz, 1H), 2.01 – 1.89 (m, 8H), 1.69 (s, 1H), 1.64 (s, 2H), 1.51 (s, 1H), 1.44 (dt, *J* = 24.7, 11.7 Hz, 10H), 0.96 (d, *J* = 6.8 Hz, 1H). ﻿**^13^C NMR** (126 MHz, Acetic Acid) δ 173.69, 161.37, 79.66, 78.97, 57.92, 55.19, 54.66, 53.85, 45.75, 29.77, 27.21, 27.00, 26.71, 25.88, 22.74, 20.49, 20.40, 19.98, 19.86. **LR-ESI-MS**: m/z calc. for C10H17N2O ([M]^+^): 181.1, found: 181.0.

**Scheme S6.** Synthesis of compound **10**

2H-Benzo[d][1,3]oxazine-2,4(1H)-dione (1.0 g, 6.13 mmol) and 2,2-diethoxyethan-1-amine (0.82 g, 6.13 mmol, 1.0 equiv) were dissolved in acetonitrile (10 mL) and the reaction mixture was heated to reflux for 2 hours. After completion, the solvent was removed under reduced pressure to afford the crude intermediate, which was used directly in the next step without purification. The crude intermediate (1.0 g) was then dissolved in ethanol (20 mL), and a few drops of concentrated sulfuric acid were added. The reaction mixture was stirred for 20 minutes at room temperature. Ethanol was then removed under reduced pressure, and the crude product was purified by silica gel column chromatography (eluent: 100% ethyl acetate) to afford compound 12 (130 mg, 20% yield). **1H NMR** (500 MHz, DMSO) δ 7.68 (dd, J = 7.8, 1.6 Hz, 1H), 7.29 (ddd, J = 8.5, 7.3, 1.6 Hz, 1H), 6.93 (s, 1H), 6.81 – 6.73 (m, 2H), 4.18 (dd, J = 14.0, 4.1 Hz, 1H), 3.77 (dd, J = 14.0, 4.0 Hz, 1H). **13C NMR** (126 MHz, DMSO) δ 171.57, 147.19, 129.61, 128.62, 121.24, 116.41, 116.25, 48.31, 44.73. **LR-ESI-MS**: m/z calc. for C10H17N2O ([M]^+^): 161.06, found: 161.2.

**Scheme S7**. Synthesis of compound **15**

2-(Prop-2-yn-1-yloxy)ethan-1-amine (1.0 g, 10.09 mmol) was dissolved in THF, followed by the sequential addition of triethylamine (TEA) (1.53 g, 15.13 mmol, 1.5 equiv) and tert-butyl (2-bromoethyl)carbamate (2.49 g, 11.1 mmol, 1.1 equiv). The reaction mixture was stirred overnight at room temperature. Upon completion, the reaction was quenched with 1 M HCl, and the aqueous phase was extracted with ethyl acetate. The combined organic layers were dried over Na₂SO₄, filtered, and concentrated under reduced pressure. The crude product was purified by silica gel column chromatography (eluent: 10% MeOH in DCM) to afford the desired intermediate. In the next step, 3-(tert-butoxycarbonyl)oxazolidine-2-carboxylic acid (300 mg, 1.37 mmol) was dissolved in DMF, followed by the addition of HATU (787 mg, 2.07 mmol, 1.5 equiv) and DIEA (892 mg, 6.91 mmol, 5.0 equiv). After stirring for 10 minutes, the previously obtained intermediate (334 mg, 1.37 mmol, 1.0 equiv) was added, and the reaction mixture was stirred overnight at room temperature. The reaction was quenched with 1 M HCl, extracted with ethyl acetate, and the organic phase was dried over Na₂SO₄, filtered, and concentrated. The crude product was purified by silica gel chromatography to afford the protected intermediate. Boc and tBu protecting groups were then removed using TFA, and the residue was dissolved in methanol. The pH was adjusted to 5–6 using cesium carbonate (Cs₂CO₃) and stirred for 30 minutes. After solvent removal, the crude product was purified by silica gel chromatography (eluent: 80% ethyl acetate in hexane) to afford compound **14**. **1H NMR** (500 MHz, CDCl3) δ 7.72 (t, J = 2.3 Hz, 1H), 4.15 (d, J = 2.4 Hz, 2H), 3.80 (ddd, J = 6.8, 6.1, 2.3 Hz, 2H), 3.73 (dd, J = 5.5, 4.5 Hz, 2H), 3.61 (dd, J = 5.5, 4.5 Hz, 2H), 3.53 (dd, J = 7.0, 6.1 Hz, 2H), 2.43 (t, J = 2.4 Hz, 1H). **13C NMR** (126 MHz, CDCl3) δ 157.07, 156.16, 74.68, 68.03, 58.33, 48.70, 46.88, 45.16. **LR-ESI-MS**: m/z calc. for C10H17N2O ([M]+): 181.21, found: 181.5.

**Scheme S8**. Synthesis of compound **TMP-Im**

2.5g (8.62 mmol) of trimethoprim was dissolved in 30 mL 48% hydrobromic acid, which had been pre-heated to 95 °C. The reaction was stirred for 20 min at room temperature and then quenched by 5 mL 50% aqueous sodium hydroxide. The reaction mixture was allowed to cool to room temperature for a minimum of 3 hours to get the white solid. The solid was redissolved in 10 mL of boiling water and subsequently chilled at -20 °C for 10 minutes before being neutralized by the gradual addition of 50% aqueous ammonium hydroxide. The solution was placed at 4 °C overnight to yield recrystallization. The phenol product (1.43g, 60% yield) was obtained by filtering the crystal and air-drying it for 30 minutes. After dissolving the phenol product (1g, 3.62 mmol, 1eq) in 10 mL of DMSO, potassium tert-butoxide (487 mg, 4.34 mmol, 1.2eq) was added, and the reaction mixture was stirred for 30 minutes at room temperature. Next, 1,4-dibromo-butane (515 μL, 4.34 mmol, 1.2eq) was added, and the mixture was stirred for two hours at room temperature. After the reaction was completed by checking LC/MASS. Saturated NaHCO_3_ was added and extracted by ethyl acetate, dried by NaSO_4_, concentrated and purified by column (5% methanol in dichloromethane) to get the 5-(4-(4-bromobutoxy)-3,5-dimethoxybenzyl)pyrimidine-2,4-diamine (0.6g, 40% yield). The product (0.6g, 1.46 mmol, 1eq) was dissolved in ethanol first, then Potassium iodide (0.48g, 2.92 mmol, 2eq) and N-Boc-1,2-diaminoethane (0.23 mg, 1.46mmol, 1eq) were added orderly. The reaction mixture was refluxed overnight. The final product (0.5g, 69% yield) was purified using a flash column in DCM with 10% methanol. To a solution of 3-(tert-butoxycarbonyl)oxazolidine-2-carboxylic acid (0.27g, 1.22 mmol, 1.2eq) in dimethylformamide was added 4-(4,6-Dimethoxy-1,3,5-triazin-2-yl)-4 -methylmorpholinium chloride (0.49g, 2.04 mmol, 2eq) and N,N-Diisopropylethylamine (355 μL, 2.04 mmol, 2eq). After pre-activating for 10 min, the tert-butyl (2-((4-(4-((2,4-diaminopyrimidin-5-yl)methyl)-2,6-dimethoxyphenoxy)butyl)amino)ethyl)carbamate (0.5g, 1.02 mmol, 1eq) was added and the whole mixture was reacted for overnight. After adding the brine to the reaction and extracted it three times using ethyl acetate, the crude product was purified using reversed-phase HPLC to produce a white solid (0.1g, 14% yield). In order to get final TMP-cyclic imine compound, tert-butyl 2-((2-((tert-butoxycarbonyl)amino)ethyl)(4-(4-((2,4-diaminopyrimidin-5-yl)methyl)-2,6-dimethoxyphenoxy)butyl)carbamoyl)oxazolidine-3-carboxylate was dissolved in a 1:1 mixture of dichloromethane and trifluoroacetic acid and stirred for 30 minutes. Subsequent solvent removal under vacuum followed by HPLC purification provided TMP-cyclic imine as a white powder (20 mg, 32% yield). 1H NMR (500 MHz, DMSO) δ 8.24 (s, 1H), 7.71 (s, 1H), 7.68 – 7.60 (m, 2H), 7.41 (t, J = 4.5 Hz, 1H), 6.57 (d, J = 3.9 Hz, 2H), 3.79 – 3.76 (m, 4H), 3.69 (s, 6H), 3.55 (s, 2H), 3.34 – 3.31 (m, 2H), 2.46 (p, J = 1.8 Hz, 2H), 1.68 – 1.58 (m, 2H), 1.53 (qd, J = 9.4, 5.1 Hz, 2H). 13C NMR (126 MHz, DMSO) δ 164.51, 159.22, 158.95, 157.04, 154.76, 153.50, 140.26, 135.62, 133.33, 115.89, 109.39, 106.67, 78.98, 72.29, 56.37, 48.06, 45.50, 43.02, 32.57, 27.33, 23.66). LR-ESI-MS: m/z calc. for C_21_H_28_N_6_O_4_ ([M]+): 429.22, found: 429.34.

**Scheme S9**. Synthesis of compound **16.**

Imine 1 (20 mg, 130 μmol) was added to 3-methyl indole (20 mg, 130 μmol) in PBS buffer with 30% acetonitrile to a final concentration of each reactant of 50 mM. The reaction was allowed to proceed 48h at RT and was then purify by RP-HPLC. (20 mg, 50% yield). 1H NMR (500 MHz, DMSO) δ 10.97 (s, 1H), 8.75 (s, 1H), 7.52 (d, J = 7.9 Hz, 1H), 7.37 (d, J = 8.0 Hz, 1H), 7.16 (t, J = 7.5 Hz, 1H), 7.03 (t, J = 7.4 Hz, 1H), 3.53 (d, J = 18.1 Hz, 1H), 3.42 (d, J = 5.3 Hz, 1H), 2.29 (s, 3H), 2.03 (dd, J = 25.0, 10.4 Hz, 2H), 1.77 (dd, J = 34.2, 6.8 Hz, 3H), 1.55 – 1.16 (m, 6H).. LR-ESI-MS: m/z calc. for C_17_H_21_N_3_O ([M]+):284.2, found: 284.2.

**Scheme S10**. Synthesis of compound **17.**

Sulfonamide imine **11** (10 mg, 60 μmol) was added to N-acetyl-l-tyrosine methyl amide (14 mg, 60 μmol) in PBS buffer with 10% acetonitrile to a final concentration of each reactant of 50 mM. The reaction was allowed to proceed overnight at RT and was then purify by RP-HPLC. (15 mg, 63% yield). 1H NMR (500 MHz, CDCl_3_) δ 7.82 /7.77 (m, 2H), 7.58 – 7.52 (m, 4H), 7.32 – 7.27 (m, 2H), 7.08/7.06 (diastereomers) (d, J =  8.2 Hz, 1H), 6.98 (d, J =2.2 Hz, 1H), 6.96 (s, 1H), 6.71 (d, 1H), 6.67/6.58 (diastereomers) (dt, J = 4.9 Hz, 1H), 6.08/6.01 (diastereomers) (s, 1H), 4.63/4.58 (q, J = 5.1Hz, 1H), 2.85 (m, 2H), 2.70/2.68 (diastereomers) (d, J = 4.8 Hz, 3H), 1.99/1.94 (diastereomers) (s, 3H). LR-ESI-MS: m/z calc. for C_21_H_28_N_6_O_4_ ([M]+): 404.1, found: 404.2. aluSee appendix 2 for LC/MS trace.

**4.1 Synthesis of cyclic imine containing CBX inhibitors**

**Synthesis of the backbone of inhibitor SW2_110A (SW2_110A_Ns)**

(SW2_110A_Ns)

As previously described ^7–9^, the inhibitor backbone was synthesized on MBHA resin, except for the utilization of Na-Fmoc-Ne-Nosyl-L-lysine building block instead of the Mtt-protected lysine.

**Scheme S11.** Synthesis of **1c** (6 carbon linker)

(N-Boc)-2-bromo-amine (3.6 mmol, 1 eq, 0.8 g) was added to 6-amino-hexanol (7.2 mmol, 2 eq, 0.84 g) with 100 mg KI in 15 mL EtOH. The reaction was heated to 70 °C and refluxed overnight. Product was purified on silca gel using a gradient of MeOH (0-10%) in DCM (1% TEA) to afford 1b (0.4415 g, 1.7 mmol, 47 %). 1.7 mmol of **1b** (1 eq) was reacted with 0.444g O-tBu-N-Boc-Serine (1 eq), 0.325 g EDC (1 eq), 0.231 g HOAt (1 eq), and 300 ul of DIEA in 2 mL DMF, overnight. Reactions were column purified to afford the final 6 carbon alcohol containing linker **1c** (0.9 mmol, 453.4 mg, 53%). **^1^H NMR** (500 MHz, CDCl3) δ 7.27 – 7.12 (m, 3H), 5.33 – 5.12 (m, 1H), 4.77 – 4.61 (m, 1H), 4.39 – 4.04 (m, 1H), 3.84 – 3.03 (m, 10H), 2.34 (d, J = 3.7 Hz, 2H), 1.43 (d, J = 15.2 Hz, 21H), 1.15 – 1.11 (m, 11H). **^13^C NMR** (126 MHz, CDCl3) δ 155.97, 155.51, 154.93, 137.71, 128.88, 128.07, 125.15, 79.62, 79.06, 73.41, 73.11, 64.97, 63.42, 62.45, 62.00, 54.12, 49.97, 48.28, 45.81, 38.58, 32.33, 32.20, 28.42, 28.27, 28.18, 27.18, 27.15, 26.27, 25.92, 25.53, 25.02, 21.32. **LR-ESI-MS**: m/z calc. for C25H49N3O7 ([M+H]^+^): 504.4, found: 504.6.

**Scheme S12.** Synthesis of **1f** (4 carbon linker)

As with the synthesis of compound **1c**, (N-Boc)-2-bromo-amine (16 mmol, 1 eq) was added to 4-amino-hexanol (32 mmol, 2 eq) with 100 mg KI in 15 mL EtOH. The reaction was heated to 70 °C, and refluxed overnight. Product was purified using gradient MeOH (0-10%) in DCM (1% TEA) to afford **1e** (0.4415 g, 1.7 mmol, 47 %). 1.7 mmol of **1e** (1 eq) was reacted with 0.444g O-tBu-N-Boc-Serine (1 eq), 0.325 g EDC (1 eq), 0.231 g HOAt (1 eq), and 300 ul of DIEA in 2 mL DMF, overnight. Reactions were column purified to afford the final 4 carbon alcohol containing linker **1f** (0.9 mmol, 453.4 mg, 53%). **1H NMR** (500 MHz, CDCl3) δ 7.24 (t, J = 7.4 Hz, 1H), 7.15 (dd, J = 12.5, 7.1 Hz, 1H), 5.33 – 5.15 (m, 1H), 4.82 – 4.63 (m, 1H), 3.85 – 3.56 (m, 4H), 3.55 – 3.09 (m, 6H), 1.72 – 1.33 (m, 22H), 1.13 (d, J = 3.6 Hz, 9H). **^13^C NMR** (126 MHz, CDCl3) δ 172.17, 156.04, 155.21, 128.92, 128.11, 125.18, 79.97, 79.17, 73.47, 63.44, 63.17, 62.08, 61.68, 49.83, 48.08, 46.04, 45.78, 38.69, 29.14, 28.98, 28.30, 28.21, 27.21, 27.16, 25.13, 23.76, 21.36. **LR-ESI-MS**: m/z calc. for C23H44N3O7 ([M+H]^+^): 476.3, found: 476.6.

**Scheme S13:** Synthesis of **P1a**

**Scheme S14:** Synthesis of **P1b**

Following the Fukuyama-Mitsunobu reaction conditions,^10–12^ 50 μmol (1 eq) of **SW2_110A_Ns** peptide on resin was mixed with 250 μmol (5 eq) of alcohol linker (6-carbon linker 1c or 4-carbon linker 1f), 0.0656 g (250 μmol, 5 eq) triphenyl phosphine (TPP), 5 eq (49 μL) diisopropyl azodicarboxylate (DIAD) in 650 μL toluene on ice. The alcohol linker and TPP was dissolved in toluene and chilled on ice for 20 mins. After the alcohol-TPP mixture solution was ice cold, ice-cold DIAD was then added, and the whole solution was added to the resin (containing **SW2_110A** with Ns-lysine) and the reaction was under 4 °C for 2 hours and followed up with slow warmup to room temperature overnight. Synthetic yield was monitored by LC/MS (40-50% synthetic yield). Reductive alkylation was followed up using 100 eq acetaldehyde, 10 eq NaCNBH_3_ in DCM:MeOH (v:v=2:1) with 1 drop of glacial acid overnight at room temperature. Peptides were cleaved off the resin using 95/2.5/2.5 TFA/H_2_O/TIPS cocktail solution for 2-3 hours at room temperature. Products **P1aL** and **P1bL** were HPLC purified and validated by LC/MS. **P1aL**, **LR-ESI-MS**: m/z calc. for C48H77N9O9 ([M+H]^+^): 924.6, ([M+2H]^2+^): 462.8 ; found: 924.9,: 463.2. **P1bL**, **LR-ESI-MS**: m/z calc. for C46H73N9O9 ([M+H]^+^): 896.5, ([M+2H]^2+^) :448.8; found: 896.8, 448.9.

Purified peptides **P1aL** and **P1bL** were oxidized with 2 eq NaIO_4_ in PBS (pH 7). Oxidized and cyclized imine formation were monitored by LC/MS. 20-30 minutes of NaIO_4_ treatment led to the complete oxidation of the starting material and cyclized imine inhibitors **P1a** and **P1b**, which were validated by LC/MS. **P1a**, **LR-ESI-MS**: m/z calc. for C47H70N8O8 ([M+H]^+^): 875.5, [M+2H]^2+^ 438.3; found: 875.7, 438.4. **P1b**, **LR-ESI-MS**: m/z calc. for C45H65N8O8 ([M+H]^+^): 847.5, [M+2H]^2+^ 424.3; found 847.6, 424.3.

**Scheme S15:** Synthesis of **P1c**/ **P1d**/ **P1e**/ **P1f**

On resin peptide **SW2_110A_Ns** (50 μM, 1 eq for each compound) was mixed with 10 eq bromoethane, 10 eq DBU in DMF overnight to obtain **SW3_72**. Then, Ns was deprotected using 10 eq DBU and 10 eq β-mercaptoethanol in DMF for 2 hours at R.T. Secondary amine upon Ns deprotection was detected by chloranil test and yield was monitored by LC/MS. Then, 5 eq of N-Fmoc-2-aminoacetaldehyde, 5 eq NaCNBH_3_, 1 drop of glacial acid were mixed with resin in DCM:MeOH (v:v=2:1) overnight for reductive alkylation and followed up by Fmoc deprotection using 20% piperidine in DMF for 1 hour. Then, N_α_-Fmoc-N_β_-Boc-L-Dap, N_α_-Boc-N_β_-Fmoc-L-Dap, N_α_-Fmoc-N_β_-Boc-D-Dap, or N_α_-Boc-N_β_-Fmoc-D-Dap were coupled to the primary amine on resin, with DIC and HOAt. Fmoc was then deprotected and coupled with Boc-Ser(tBu)-OH. Peptides were then cleaved off the resin using 95/2.5/2.5 TFA/H_2_O/TIPS cocktail solution for 2-3 hours at room temperature and purified by HPLC to afford compounds **P1c/d/e/f**. Purified peptides were oxidized with 2 eq NaIO_4_ in PBS. Oxidized and cyclized imine formation were monitored by LC/MS. 30 minutes of NaIO_4_ treatment led to the complete oxidation of the starting material and cyclized imine formation.

LR-ESI-MS: m/z calc. for **P1c** [M+H]^+^.: 862.5; found [M+H]^+^: 862.6, [M+2H]^2+^: 432.0

m/z calc. for **P1d** [M+H]^+^.: 862.5; found [M+H]^+^: 862.6

m/z calc. for **P1e** [M+H]^+^.: 862.5; found [M+H]^+^: 862.7, [M+2H]^2+^: 431.9

m/z calc. for **P1f** [M+H]^+^.: 862.5; found [M+H]^+^: 863.0, [M+2H]^2+^: 432.4

**Scheme S16:** Synthesis of **P3a**

On resin peptide **SW2_110A_Ns** (50 μM, 1 eq for each compound) was mixed with 10 eq bromoethane, 10 eq DBU in DMF overnight to obtain **SW3_72**. Then Ns was deprotected using 10 eq DBU and 10 eq β-mercaptoethanol in DMF for 2 hours at R.T. Secondary amine upon Ns deprotection was detected by chloranil test and yield was monitored by LCMS. Then, 5 eq of N-Fmoc-2-aminoacetaldehyde, 5 eq NaCNBH_3_, 1 drop of glacial acid were mixed with resin in DCM:MeOH (v:v=2:1) overnight for reductive alkylation, and followed up by Fmoc deprotection using 20% piperidine in DMF for 1 h. Boc-N-(2-Fmoc-aminoethyl) glycine was coupled to amine, with DIC/HOAt in DMF for 1 h, and Fmoc was deprotected for 30 min using 20% piperidine in DMF. Then, Boc-Ser(tBu) was coupled using DIC/HOAt in DMF for 1h. Upon synthesis completion, peptide **P3aL** was cleaved off the resin with H_2_O/TIPS/TFA (2.5:2.5:95) for 2h at R.T. Purified peptide **P3aL** was then treated with sodium periodate in PBS (pH 7). Cyclized iminium inhibitor **P3a** was formed in 30 mins and validated by LC/MS. LR-ESI-MS: m/z calc. for **P3a** [M]^+^.: 876.5; found [M+H_2_O]^+^: 894.8, [M]^+^: 876.7, [M+H_2_O+H]^2+^: 446.9, [M+H]^2+^: 439.2

**Scheme S17:** Synthesis of **P3b**

**

On resin peptide **SW2_110A_Ns** (50 μM, 1 eq for each compound) was mixed with 5 eq 2-(Fmoc-amino) ethanol (or Fmoc-glycinol), 5 eq TPP, 5 eq DIAD at 4 degree for 2 hours and slowly warmed up to R.T. overnight, following Fukuyama-Mitsunobu conditions. Fmoc was then deprotected using 20% piperidine in DMF for 30 mins. Boc-Ser(tBu)-OH was then coupled to the primary amine with DIC/HOAt in DMF for 1 hour at R.T. Next, Ns was deprotected using β-merceptoethanol/DBU in DMF for 2 hours at R.T. Secondary amine upon Ns deprotection was detected by chloranil test and yield was monitored by LCMS. Upon synthesis completion, peptide **P3bL** was cleaved off the resin with H_2_O/TIPS/TFA (2.5:2.5:95) for 2h at R.T. Purified peptide **P3bL** was then treated with sodium periodate in PBS (pH 7). Cyclized iminium inhibitor **P3b** was formed in 30 mins and validated by LC/MS.

**Scheme S18:** Synthesis of **P1bP**

Modified methods from previously reported procedure were used to synthesize C-terminal alkyne peptides. Polystyrene-linked aldehyde resin (FMPB AM resin, 100 mg, 1.08 mmol/g) was added to a round bottom flask and gently stirred for 30 minutes at RT in DCM. DCM was gently evaporated and 5 mL of DMF with 5 mL of MeOH was added to the resin. To this, 10 eq. glacial AcOH was added with 10 eq. propargyl amine and 10 eq. NaCNBH_3_ and gently stirred under light reflux for 3 hours at 80 °C. The mixture was cooled and washed with MeOH, DCM, and DMF and re-swelled for 30 mins in 1,2-dichloroethane prior to the first acylation. To the resin, 5.0 eq. Fmoc-Ser(OtBu)-OH with 5.0 eq. DIC, 8.0 eq. HOAt in DMF was added and incubated at 37 °C overnight. The remaining synthesis was completed as described above. On resin peptide **SW2_110A_Ns** (50 μmols, 1 eq) was mixed with 5 eq 6 carbon linker alcohol **1c**, 5 eq TPP, 5 eq DIAD at 4 degree for 2 hours and slowly warmed up to R.T. overnight, following Fukuyama-Mitsunobu conditions. Next, Ns was deprotected using β-merceptoethanol/DBU in DMF for 2 hours at R.T. Secondary amine upon Ns deprotection was detected by chloranil test and yield was monitored by LCMS. Following reductive alkylation was performed using 10 eq acetaldehyde and 10 eq NaCNBH_3_ in co-solvent DCM:MeOH (3:1) at r.t. for 2 hours. Upon synthesis completion, peptide **P1bPL** was cleaved off the resin with H_2_O/TIPS/TFA (2.5:2.5:95) for 2h at R.T.

A small portion (~1 mg) was purified by RP-HPLC, dried, and resuspended in 50:50 water:acetoniltrile with 0.1% formic acid for high resolution analysis. HR-ESI-MS: m/z calc. for **P1bPL** [M+H]^+^.: 962.60735; found [M+H]^+^: 962.60626 (-1.13 ppm error).

Purified peptide **P1bPL** was treated with sodium periodate in PBS (pH 7) at r.t. Cyclized iminium inhibitor **P1bP** was formed in 30 mins and validated by LC/MS. LR-ESI-MS: m/z calc. for **P1bP** [M+H]^+^.: 913.6; found [M+H]^+^: 913.6, [M+2H]^2+^: 457.3. A small portion (~1 mg) was purified by RP-HPLC, dried, and resuspended in 50:50 water:acetoniltrile with 0.1% formic acid for high resolution analysis. HR-ESI-MS: m/z calc. for **P1bP** [M+H]^+^.: 913.55459; found [M+H]^+^: 913.55389 (-0.77 ppm error).

**Scheme S19:** Synthesis of **P2P**

Compound **2a** was initially synthesized off-resin. 1 eq Boc-Ser(tBu) (130.7 mg, 0.5 mmol, 1 eq.) was pre-activated with DIC (62.5 mg, 0.5 mmol, 1 eq.), and added 1 eq Boc-Ser (102.6 mg), with DMAP (62 mg, 0.5 mmol, 1 equ.) in DCM. Reaction mixture was stirred at 0 for 1h, and slowly warm up to R.T. for 16 hours. The mixture extracted against brine and concentrated. The crude product **2a** was used for a subsequent on-resin coupling without further purification.

Peptide backbone (alkyne terminal) was synthesized similarly as **SW2_110A_Ns** above. FMPB AM resin (Novabiochem) was used for SPPS. Propargyl amine was coupled to the resin as previously described. Other building blocks remain the same as **SW2_110A_Ns**. Final peptide backbone on resin was named as **SW2_110A_Ns_alkyne** in the following session. On resin peptide **SW2_110A_Ns_alkyne** (50 μM, 1 eq for each compound) was mixed with 10 eq bromoethane, 10 eq DBU in DMF overnight. Then Ns was deprotected using 10 eq DBU and 10 eq β-mercaptoethanol in DMF for 2 hours at R.T. Secondary amine upon Ns deprotection was detected by chloranil test and yield was monitored by LCMS. Then, 5 eq of N-Fmoc-2-aminoacetaldehyde, 5 eq NaCNBH_3_, 1 drop of glacial acid were mixed with resin in DCM:MeOH (v:v=2:1) overnight for reductive alkylation and followed up by Fmoc deprotection using 20% piperidine in DMF for 1 hour. **2a** was coupled to primary amine with HOAt/DIC in DMF for 1h. After reaction was completed, peptide was cleaved off the resin, and HPLC purified. Purified peptide **P2PL** was treated with 5 eq. sodium periodate in PBS at pH 3. Note that acidic conditions are critical to avoid the O-N acyl shift competing reaction. The cyclized ester imine inhibitor was formed within 30 mins, validated by LC/MS. LR-ESI-MS: m/z calc. for **P2P** [M+H]^+^.: 901.5; found [M+H]^+^: 901.4.

**4.3 Synthesis of DNA conjugates**

**Scheme S20: Synthesis of DNA-Conjugate 1**

The DNA (amine DNA) used in for conjugation reaction is a single stranded 20-mer with a 5’ amine modification with a C12 alkyl linker purchased from IDT. (5’-/5AmMC12/TAC ATA GCT GCA GGC CAC TA -3’).

**General procedure for DNA precipitation**: The DNA reaction mixture was treated with 10% (v/v) of 5 M NaCl and 3 volumes of 100% ethanol. The mixture was then placed at -20°C for 1 hour. The DNA pellet was collected by centrifugation for 30 minutes, followed by washing with 80% ethanol. After removing the supernatant, the pellet was air-dried for 5 minutes

**The preparation of 1a:** Amine DNA (30 nmol) was dissolved in borate buffer (150 mM, pH=9.5, 80 μL), the 5-(1,3-dioxolan-2-yl)pentanoic acid (100mM, 100 μL), HATU (200 mM, 50 μL), DIEA (200 mM, 50 μL) were mixture and pre-activated for 5 min. Then 80 μL of mixture was added to the DNA solution. The reaction was reacted for overnight. Then the product was afforded by ethanol precipitation. The DNA was dissolved in 20 μL DI water for the use of next step. Reaction progress was confirmed by LC/MS. Expected mass: 6505; Mass found: 6503.

**The preparation of 1b:** A mixture of 39 µL of deionized water and 1 µL of 2% (v/v) acetic acid was added to the DNA conjugate solution 1a (30 nmol, 10 µL). The reaction was carried out at 50 °C for 1 hour, followed by DNA precipitation. Reaction progress was confirmed by LC/MS. Expected mass: 6461; Mass found: 6460.

**The preparation of 1c:** The DNA-conjugate 1b (25 nmol) was first dissolved in 70 µL of MES buffer (500 mM, pH 6). Then, tert-butyl (2-aminoethyl)carbamate (400 mM, 10 µL) was added to the DNA solution in MES buffer, followed by vortexing and a pre-reaction at room temperature for 30 minutes. After 30 minutes, NaCNBH_3_ (200 mM, 40 µL) in DMSO was added to the reaction mixture, which was then incubated at 40 °C for 16 hours. Upon completion, the product was recovered by ethanol precipitation. Reaction progress was confirmed by LC/MS. Expected mass: 6605; Mass found: 6604.

**The preparation of 1d:** To the DNA-conjugate 1c (25 nmol) in 40 µL of MOPS buffer (500 mM, pH 8.2), 3-(tert-butoxycarbonyl)oxazolidine-2-carboxylic acid in DMSO (500 mM, 15 µL) was added, followed by DMTMM in MOPS buffer (400 mM, 15 µL) and N-methylmorpholine in DMSO (400 mM, 30 µL). The resulting mixture was reacted at room temperature overnight. Upon completion, the DNA was isolated by ethanol precipitation and analyzed by LC-MS. Expected mass: 6804; Mass found: 6801.

**The preparation of DNA-conjugate 1:** 20 nmol of 1d was dissolved in 50 µL of sodium borate buffer (250 mM, pH 9), and the reaction was carried out at 80 °C overnight. The DNA product was obtained by ethanol precipitation, purified by HPLC, and characterized using LC-MS. Expected mass: 6542; Mass found: 6541.

**Scheme S21: Synthesis of on-DNA P1bPL**

The DNA (amine DNA) used in for conjugation reaction is a single stranded 20-mer with a 5’ amine modification with a C12 alkyl linker purchased from IDT. (5’-/5AmMC12/TAC ATA GCT GCA GGC CAC TA -3’).

**The preparation of azide-DNA:** To a solution of 80 µL borate buffer (150 mM, pH 9) containing 20 nmol amine DNA, 80 µL of a pre-mixture consisting of 6-azidohexanoic acid (100 mM, 100 µL), HATU (200 mM, 50 µL), and DIEA (200 mM, 50 µL) was added. The reaction was carried out at room temperature overnight. Upon completion, the DNA pellet was collected by ethanol precipitation and used directly in the subsequent step. Expected mass: 6488; Mass found: 6487.

**The preparation of DNA-P1bL and DNA-P1b conjugate:** To a solution of DNA with the 5’-azidohexanoic acid modification (20 nmol) in 29.5 µL of deionized water, 3 µL of DMSO and 7 µL of phosphate buffer (200 mM, pH 7) were added. Then, 2.5 µL of **P1bL** (5 mM) was introduced to the mixture. Subsequently, 3 µL of BTTAA/CuSO₄ (5:1, 50 mM) and 5 µL of sodium ascorbate solution (50 mM in phosphate buffer) were added sequentially. The reaction mixture was incubated at room temperature overnight. Following completion, the product was precipitated using ethanol and purified by HPLC to yield the DNA–P1bL conjugate. Next, 5 nmol of the purified DNA–P1bL was dissolved in 100 µL of PBS buffer (100 mM, pH 7) containing 1 mM NaIO₄ and incubated for 10 minutes. The resulting crude product was used directly for protein labeling. Expected mass: 7418; Mass found: 7414.

**5. Reactions of cyclic imines**

**5.1 General procedure for determination of second order rate constants** N-acetyl-l-tyrosine methyl amide (Ac-Tyr-NHMe) (100 mM) was incubated with 5 mM imine **1** in PBS (pH 7) at 37 °C or R.T.. Mannich reaction conversion was monitored using HPLC at different time points by integration of the cyclic imine starting material peak and Mannich product peak.

**5.2 Selectivity of cyclic imine to other amino acids** 5 mM imine **1** was incubated with 100 mM compounds mimicking amino acid side chain functional groups in PBS (pH 7) at 37 °C for 24 hours. Compounds include β-mercaptoethanol (cysteine), imidazole (histidine), isopropanol (serine/threonine), n-butylamine (lysine), propionic acid (aspartic acid/glutamic acid), 3-methyl indole (tryptophan), and N-ethyl pyrrole (pyrrole-lysine). After 24 hours, reaction mixtures were characterized by LC/MS.

**6. Stability and reversibility of cyclic imines with tyrosine and cysteine**

**6.1 Stability of Mannich product in different conditions** 5 mM compound 1 was incubated with 100 mM N-acetyl-l-tyrosine methyl amide in PBS (pH 7). After 24 hours of test reactions in PBS (pH 7) at 37 °C, reaction mixture was incubated with 1% TFA or 1% NaOH or PBS buffer for another 96 hours. Mannich product was quantitated by HPLC UV peak integration and normalized to non-treated control, to evaluate the stability of the product in acidic/basic/neutral conditions.

**Competition reaction of glutathione and N-acetyl-l-tyrosine methyl amide with compound 1**

5 mM compound 1 was incubated with 100 mM acetyl tyrosine methyl amide and 100 mM GSH (in presence of 100 mM TCEP) in a competition reaction assay. After 24 hour reaction at 37 °C in PBS (pH 7) buffer, crude reaction mixture was directly characterized by LC/MS.

**Quantitative time-dependent evaluation of thiol effect on tyrosine labeling with compound 1** Imine **1** (5 mM) was incubated with 100 mM acetyl tyrosine methyl amide and 5 mM GSH (in the presence of 5 mM TCEP). Reaction yield was quantified based on UV integrations of Mannich product at 4h, 9h, 17h, 24h, 32h. Imine consumption rate was quantified based on the UV integrations of compound 1 at 4h, 9h, 17h, 24h, 32h.

**7. Covalent protein labeling of cyclic imine-containing inhibitors**

**7.1 Covalent labeling of cyclic imine containing inhibitors to CBX8 ChD, CBX2 ChD or CBX8 Y39F ChD** For all inhibitors, 10 μM purified CBX ChD (or CBX2 ChD or CBX8 Y39F ChD) was incubated with imine covalent inhibitors (10 μM) in PBS buffer (pH 7) at 37 °C or R.T. for 12 hours overnight. For **P2**, 10 μM purified CBX ChD was incubated with **P2** (10 μM) in PBS buffer (pH 7) at 37 °C or R.T. for 1.5 hours, as well as 12 hours overnight. Samples were precipitated using MeOH and CHCl_3_ at the volume ratio of MeOH:CHCl_3_:sample=3:0.5:1, washed twice using cold MeOH, and air dried. Dried protein pellets were resuspended in aqueous buffer (0.1% formic acid, 300 mM ammonium acetate), and protein labeling were characterized and quantitated by ESI LC-MS.

**Time-dependent fluorescence displacement assay** Fluorescence polarization (FP) was measured by titration of CBX ChDs (CBX8 ChD/CBX8 ChD Y39F/CBX2 ChD) to a fluorescein-labelled probe as previously reported.^8^ Fluorescence displacement assays were performed in black 384-well plates with optical bottoms. Buffer used in FP assays consists of 100 mM Na_2_HPO_4_, 150 mM NaCl, 0.02% Tween 20. The FAM-labeled probe was kept constant at 100 nM with 2 µM CBX8 ChD, 2 µM CBX8 ChD Y39F or 2 µM CBX2 ChD, concentrations selected based on the reported relative affinity of the CBX ChD protein for the FAM probes. Two-fold dilutions of covalent inhibitors were used, starting with 100 µM as highest peptide concentration to 0.049 µM as the lowest. Four replicates were tested at each concentration. Fluorescence anisotropy was read at different time points. For FP assay with **P1a** and **P1b** against CBX8 ChD, probe **SW2_113** (K_d_ to CBX8 ChD = 0.8 μM) was used, and fluorescence signal was read at 0/1/2/4/8/16/20 hours. For FP assay with **P1a** and **P1b** against CBX2 ChD, probe **SW2_123** (K_d_ to CBX2 ChD = 1 μM) was used, and fluorescence signal was read at 0/4/16 hours. For FP assay with **P2** against CBX8 ChD, probe **SW2_113** (K_d_ to CBX8 ChD = 0.8 μM) was used, and fluorescence signal was read at 0/20/40/60/120/240 mins. Raw data were analyzed using GraphPad Prism 7 following a “one site-Fit logIC_50_” competition model with any outliers (95 % confidence interval) being excluded.

**Supplementary Spectra**

**8. Appendix 1: NMR Spectra**

^1^H-NMR of compound 1 in CDCl3 (500 MHz)


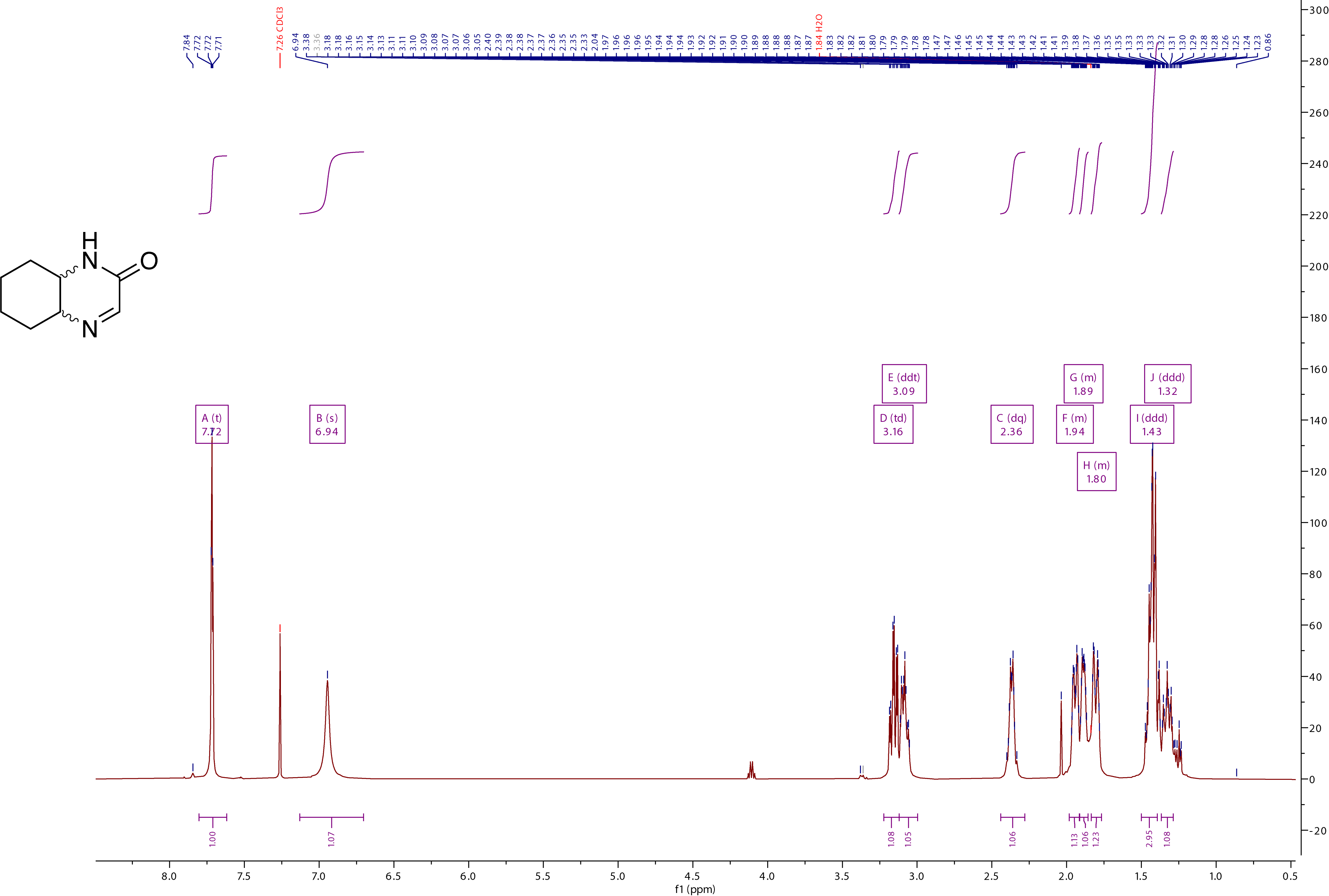


^1^H-NMR of compound 1 in PBS buffer pH=7 (500 MHz)


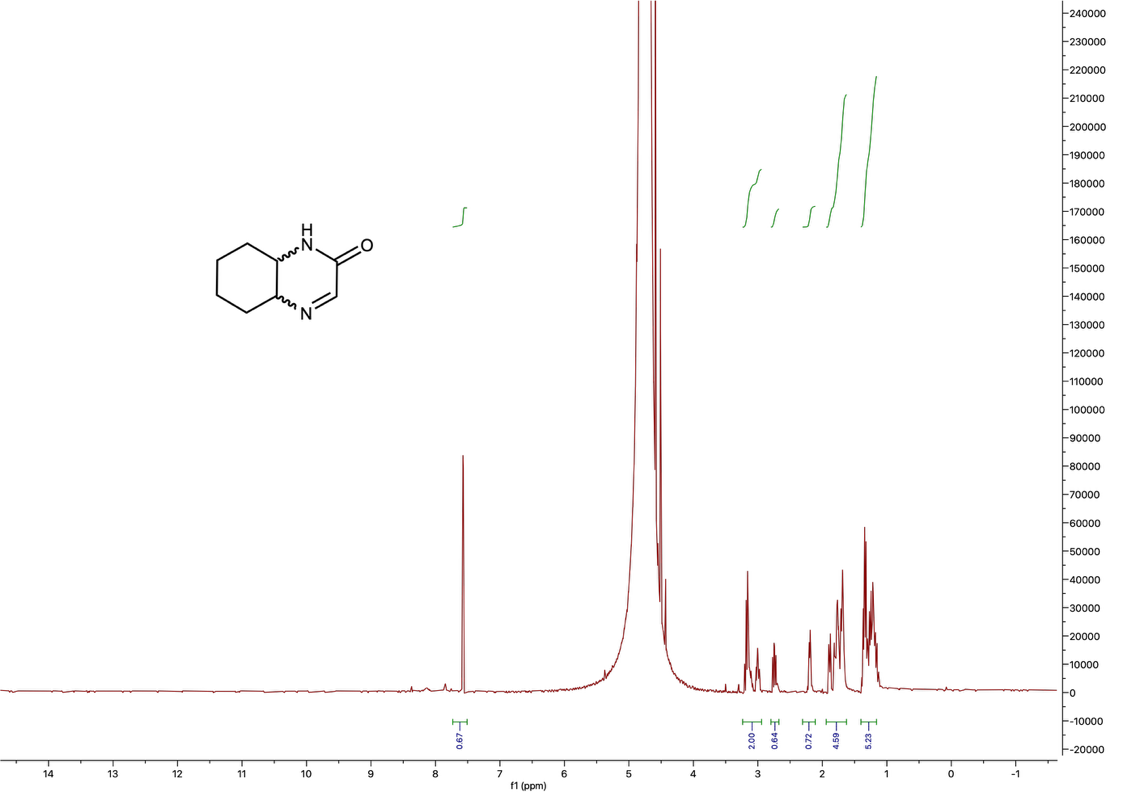


^13^C-NMR of compound 1 in CDCl3 (126 MHz)


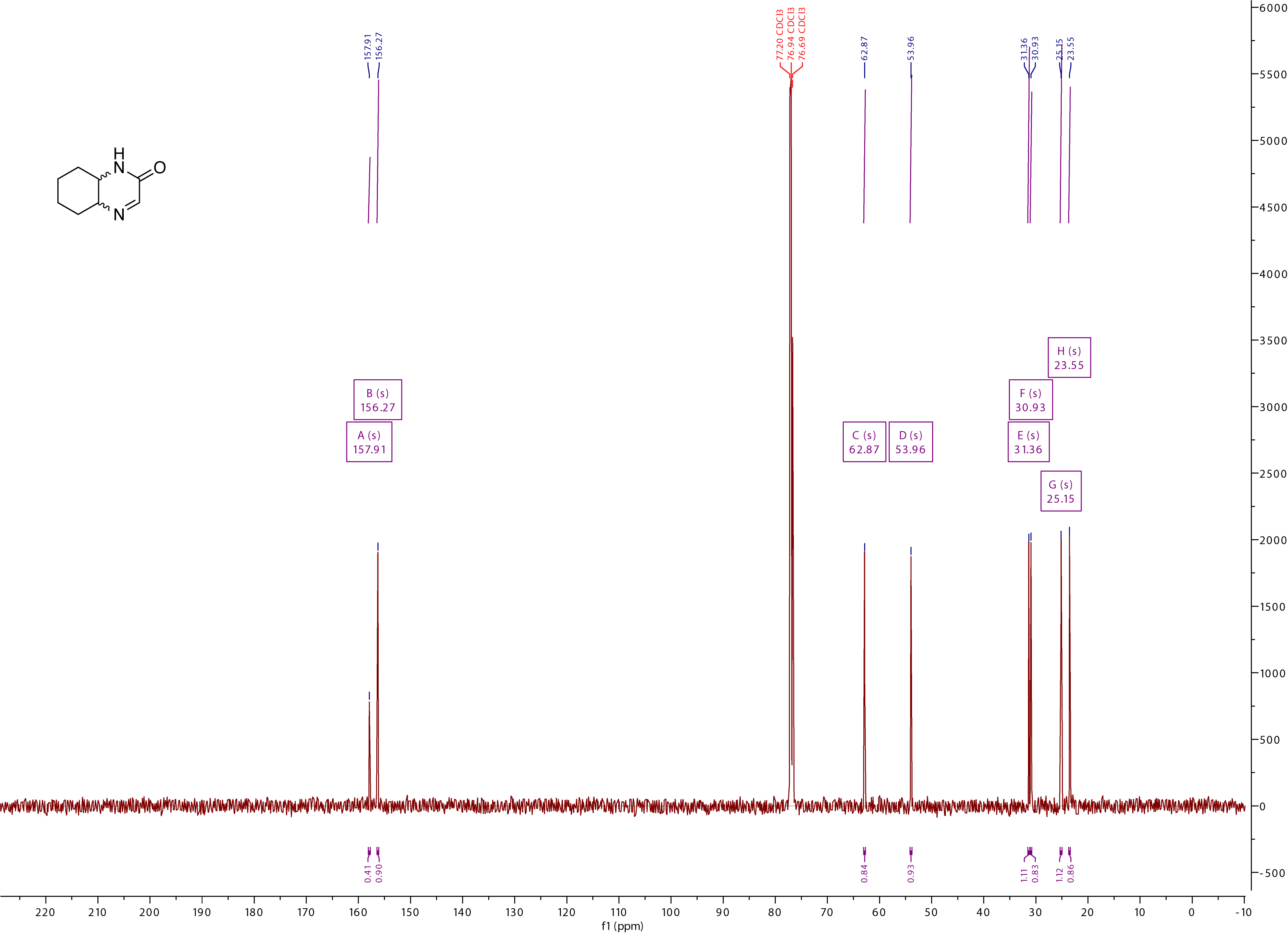


^1^H-NMR of compound 2 in CDCl3 (500 MHz)


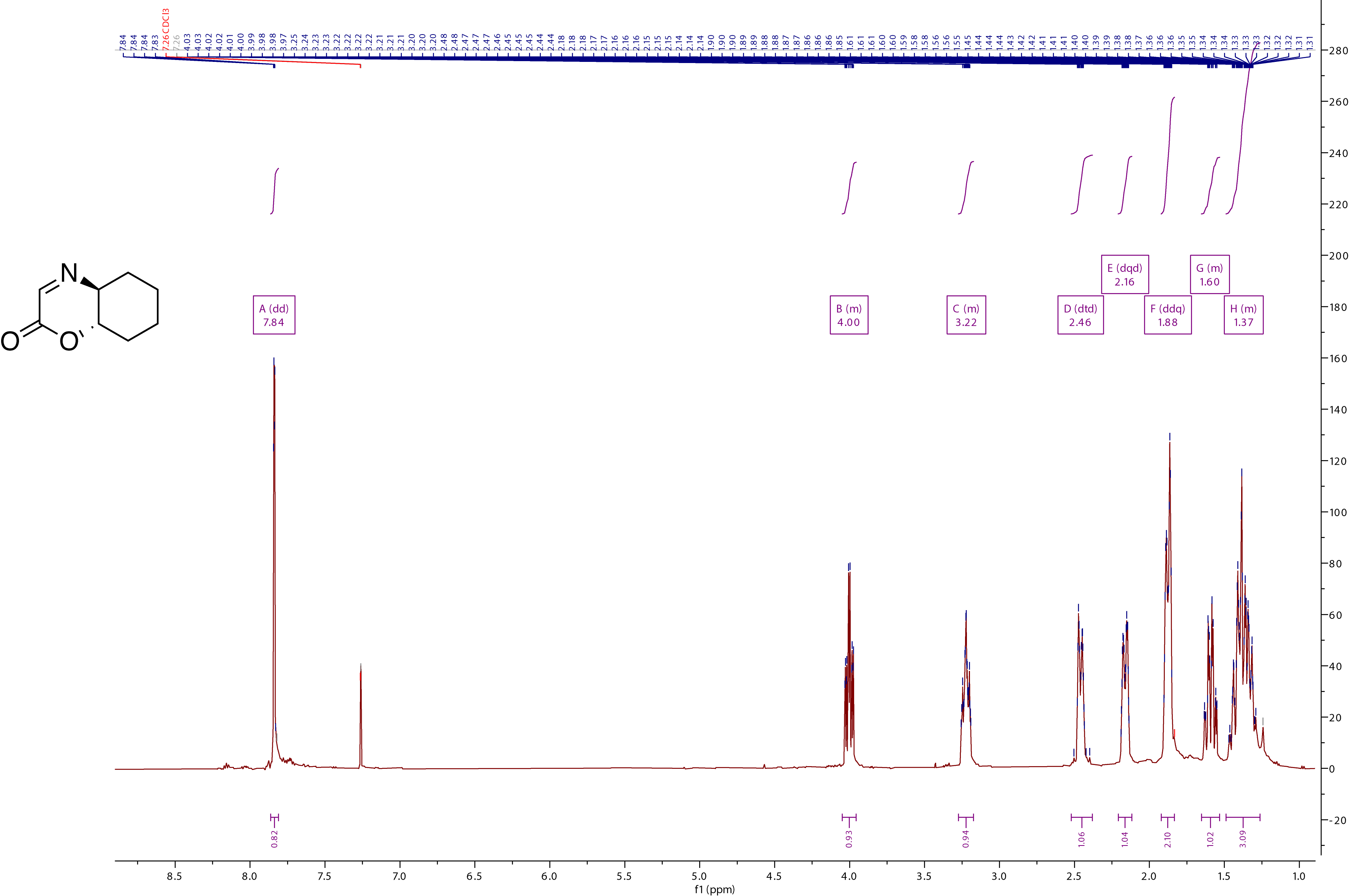


^13^C-NMR of compound 2 in CDCl3 (126 MHz)


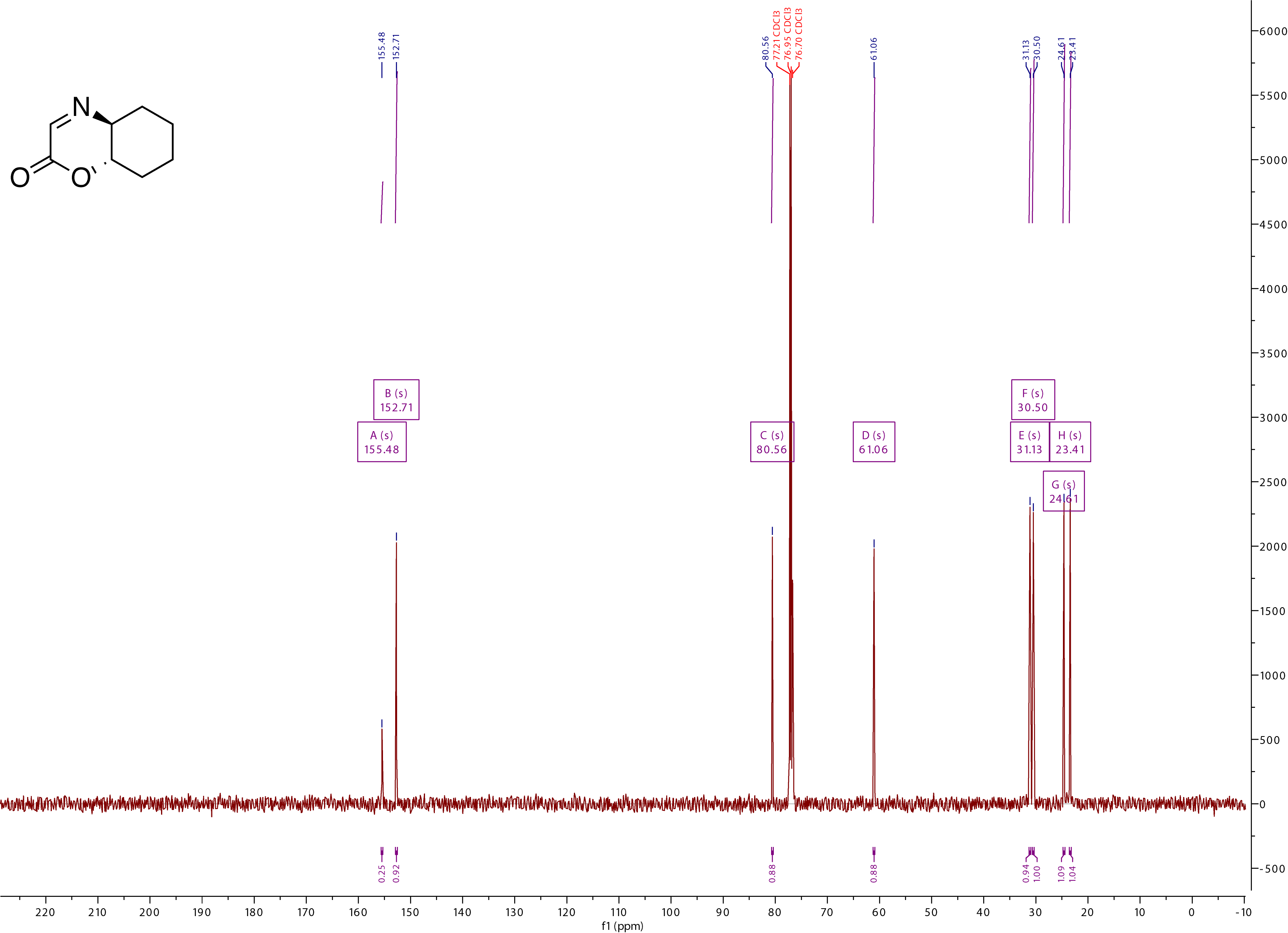


^1^H-NMR of compound 3 in CDCl3 (500 MHz)


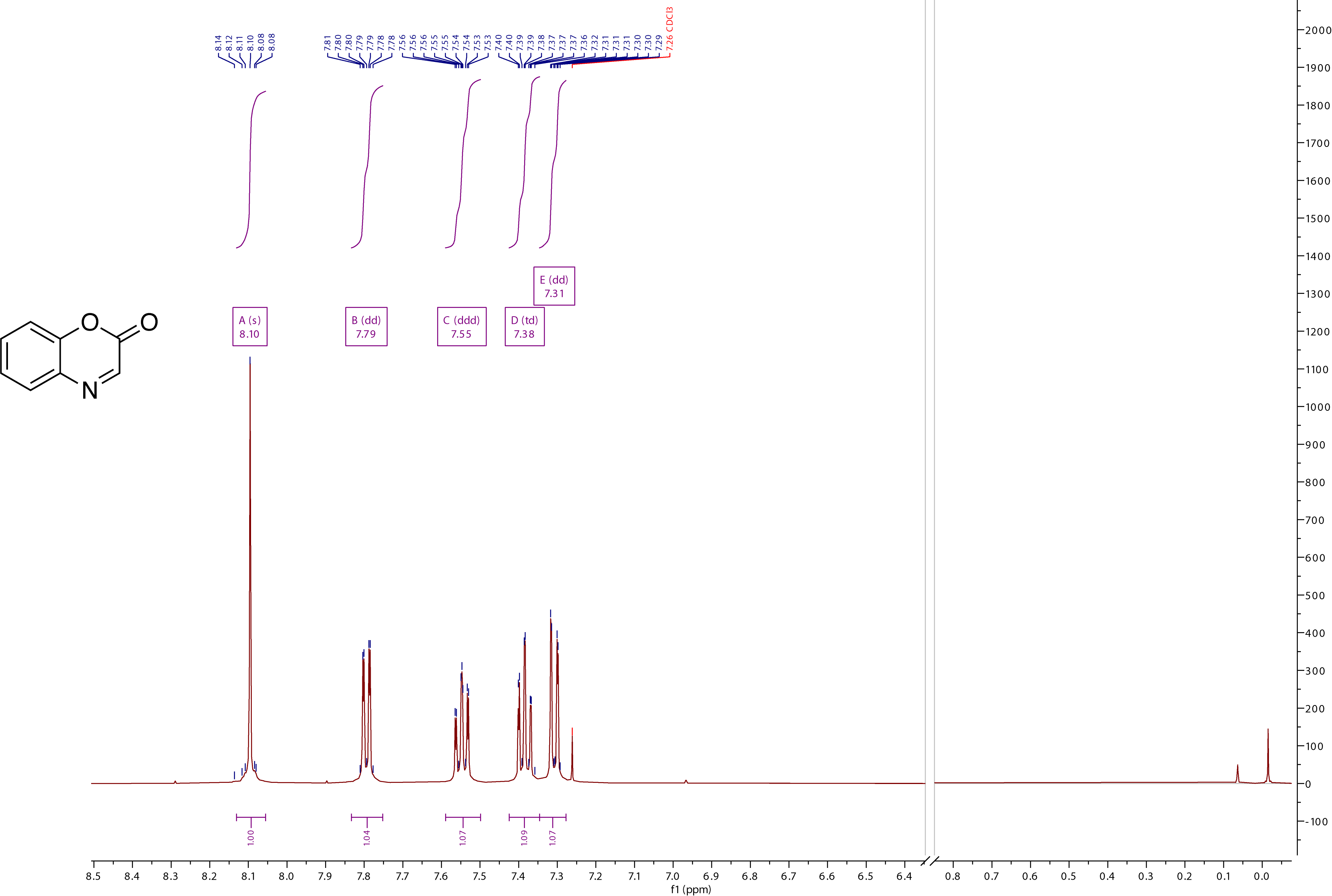


^13^C-NMR of compound 3 in CDCl3 (126 MHz)


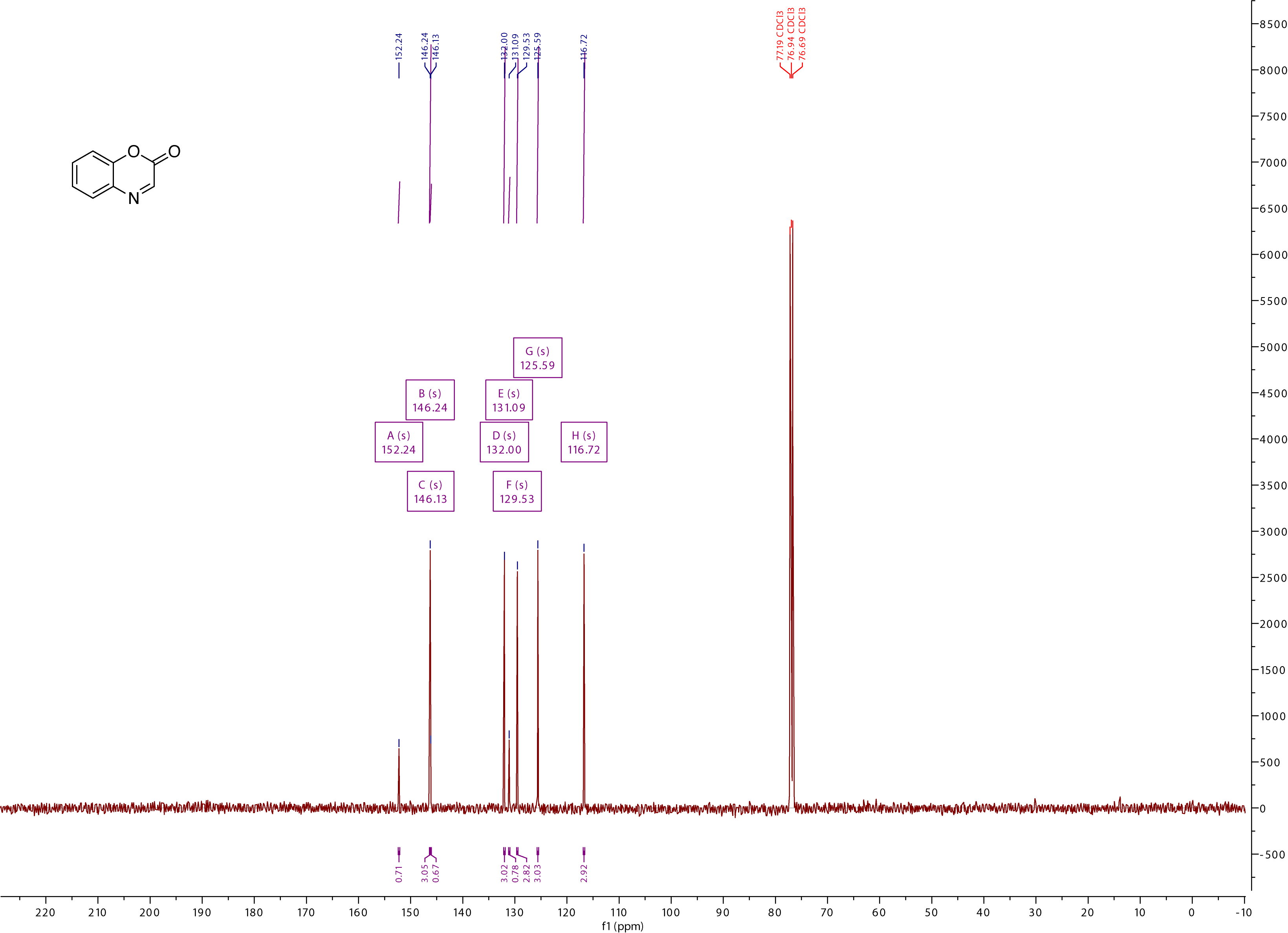


^1^H-NMR of compound 4 in CDCl3 (500 MHz)

^13^C-NMR of compound 4 in CDCl3 (500 MHz)

^1^H-NMR of compound 5 in CDCl3 (500 MHz)


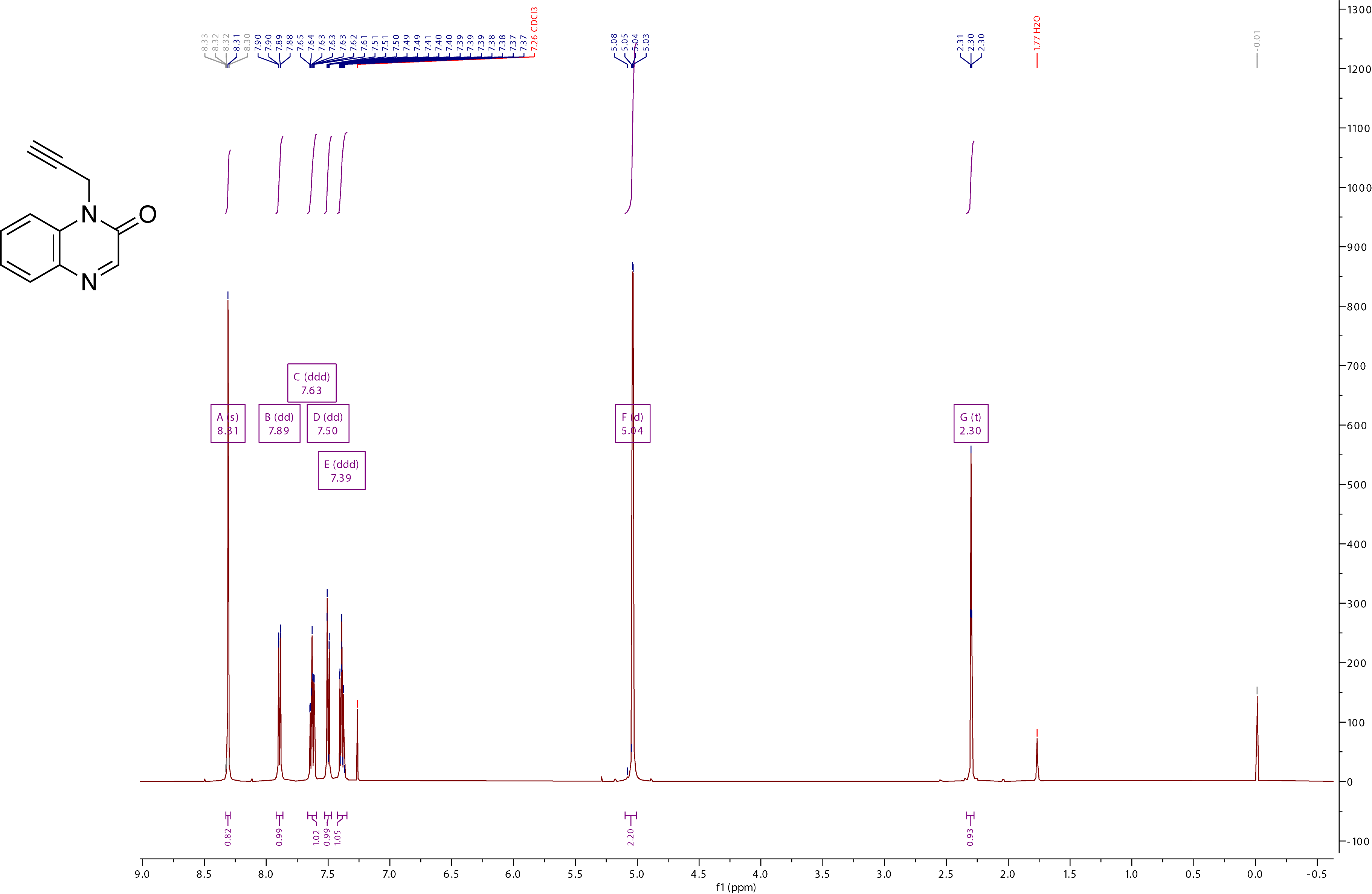


^13^C-NMR of compound 5 in CDCl3 (126 MHz)


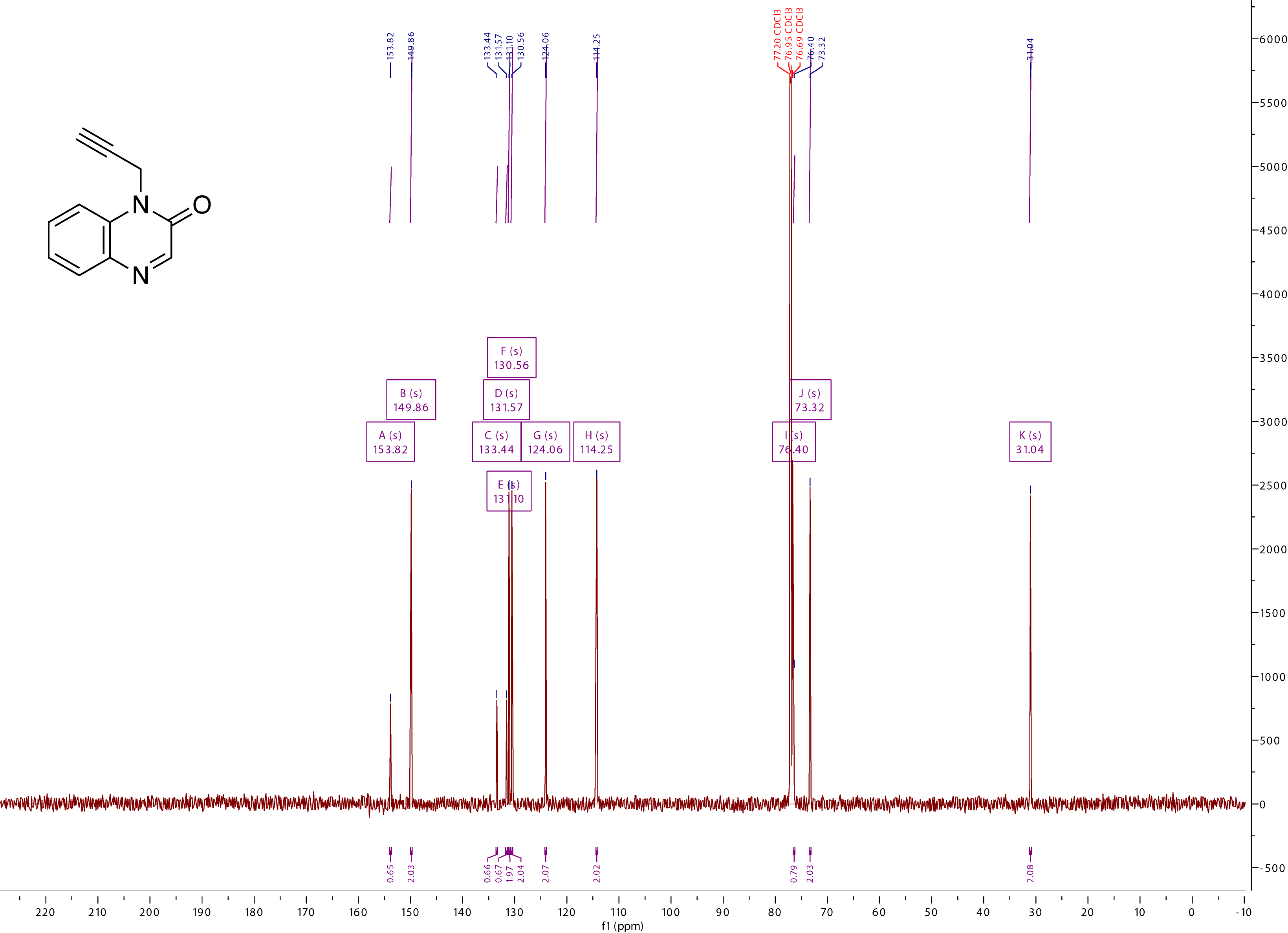


^1^H-NMR of compound 5 in Acetic acid (500 MHz)

^13^C-NMR of compound 5 in acetic acid (126 MHz)


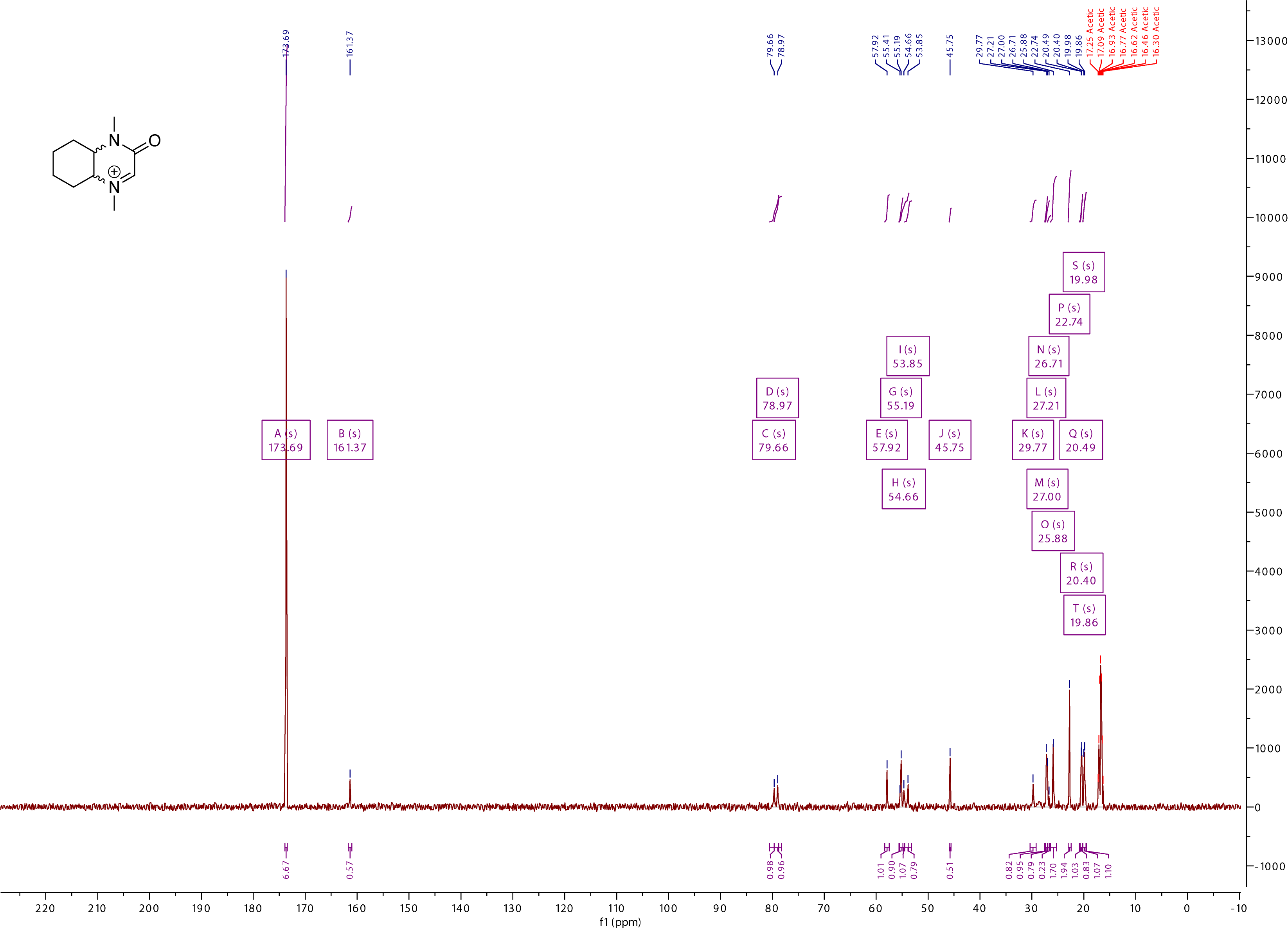


^1^H-NMR of compound 8 in CDCl3 (500 MHz)


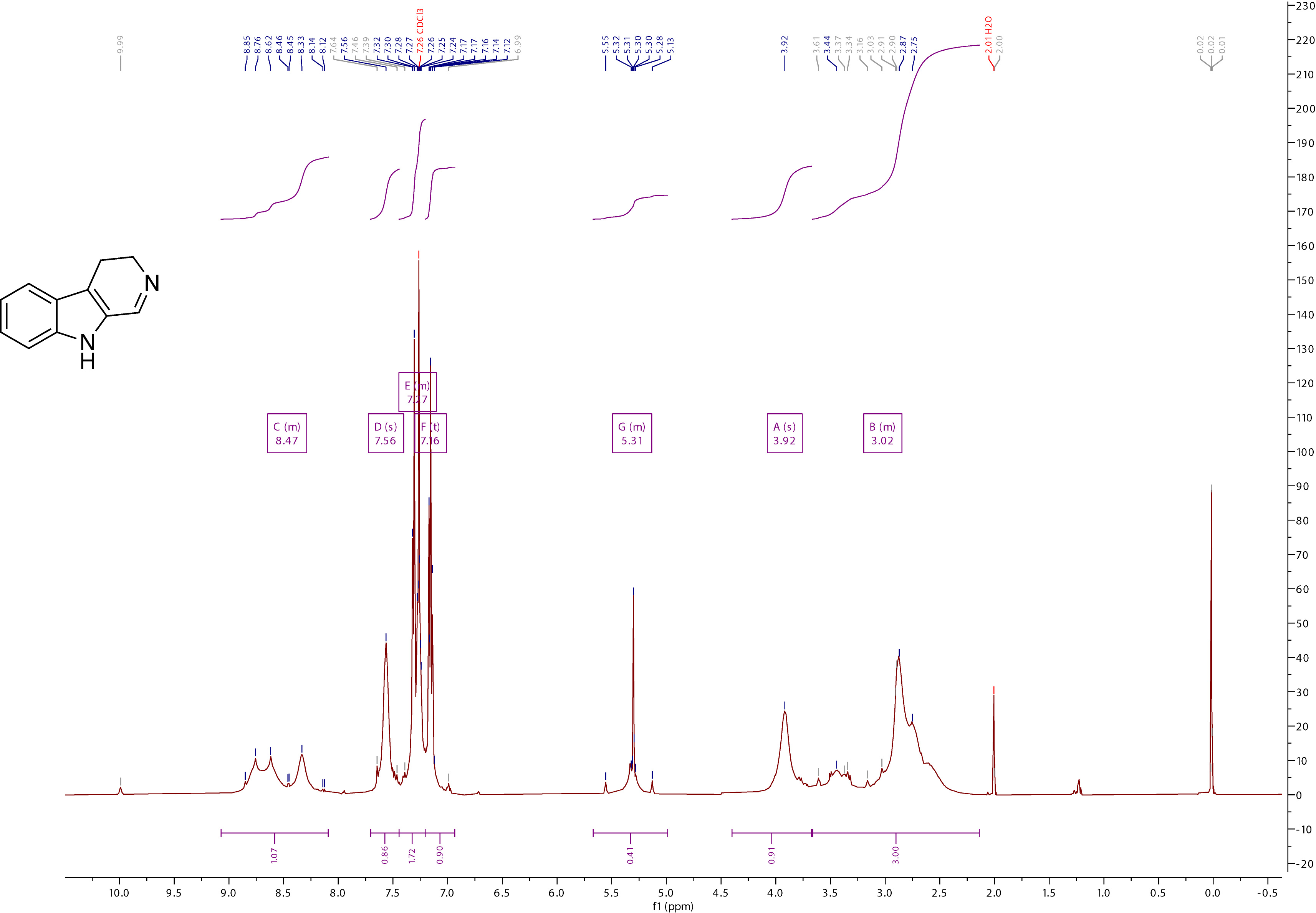


^1^H-NMR of compound 10 in DMSO (500 MHz)

^13^C-NMR of compound 10 in DMSO (500 MHz)

^1^H-NMR of compound 15 in CDCl3 (500 MHz)

^13^C-NMR of compound 15 in CDCl3 (500 MHz)

^1^H-NMR of compound 16 in DMSO (500 MHz)

^1^H-NMR of compound 17 in CDCl3 (500 MHz)

^^

^1^H-NMR of compound 1c (6-carbon linker) in CDCl3 (500 MHz)


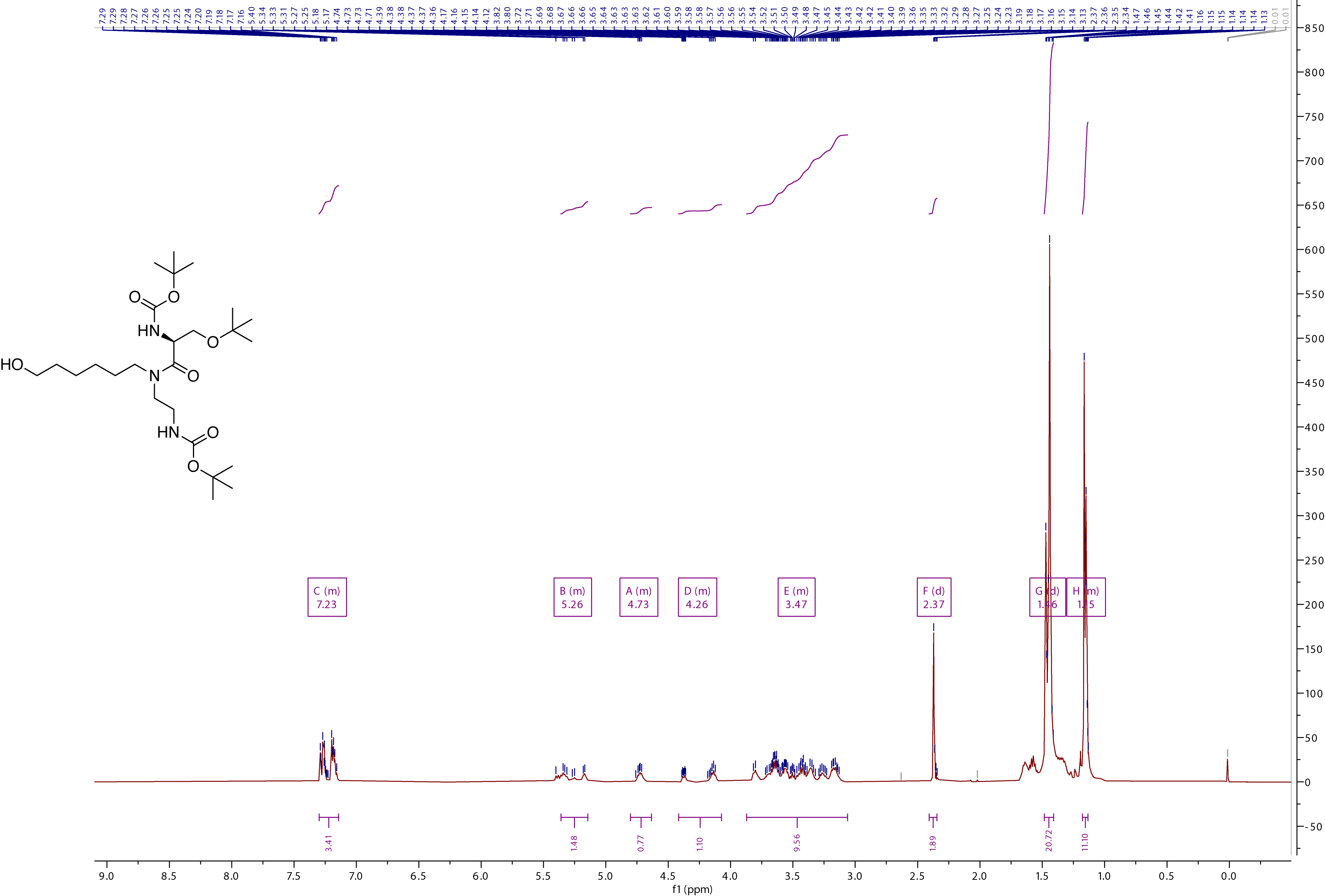


^13^C-NMR of compound 1c (6-carbon linker) in CDCl3 (126 MHz)


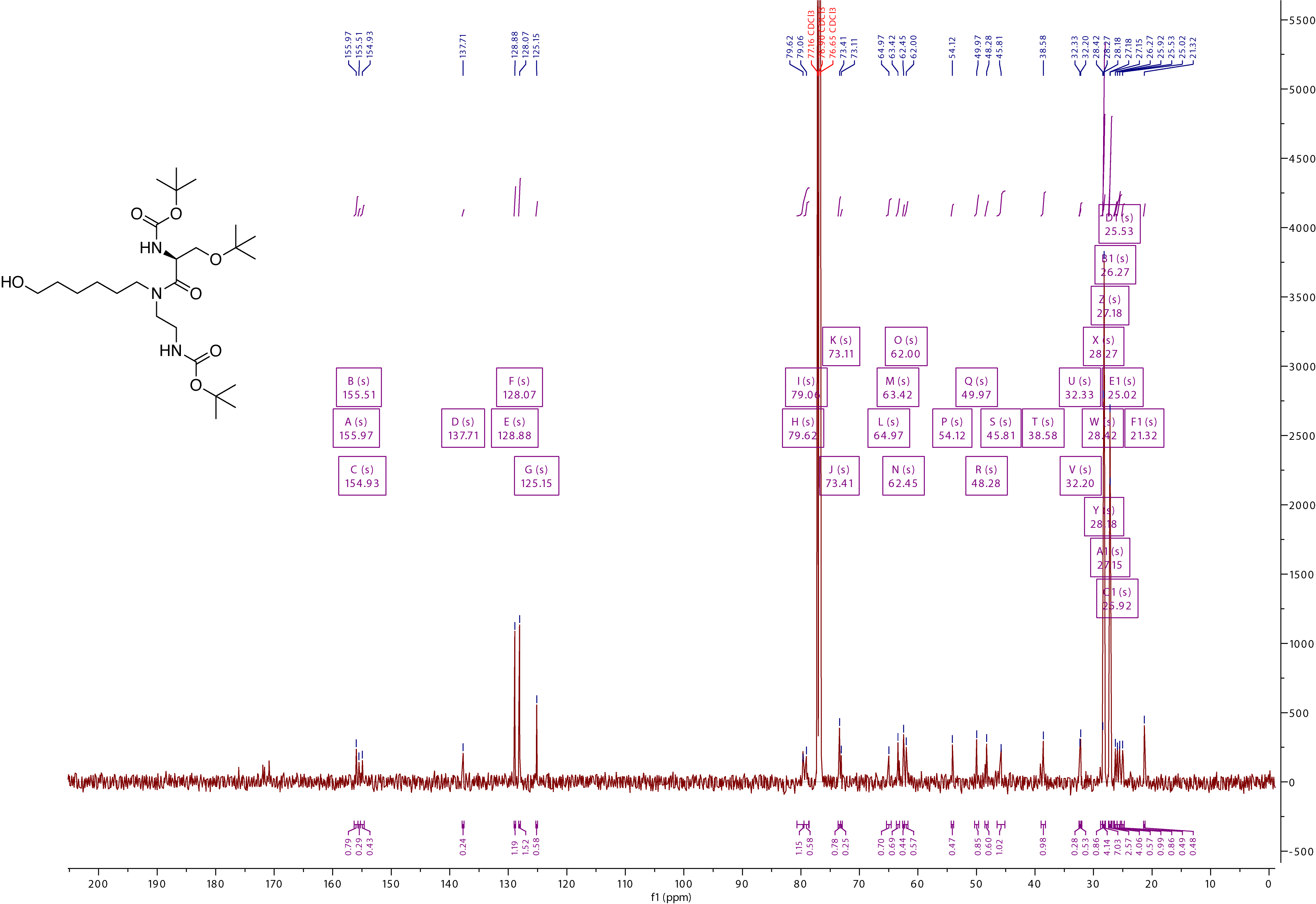


^1^H-NMR of compound 1f (4-carbon linker) in CDCl3 (500 MHz)


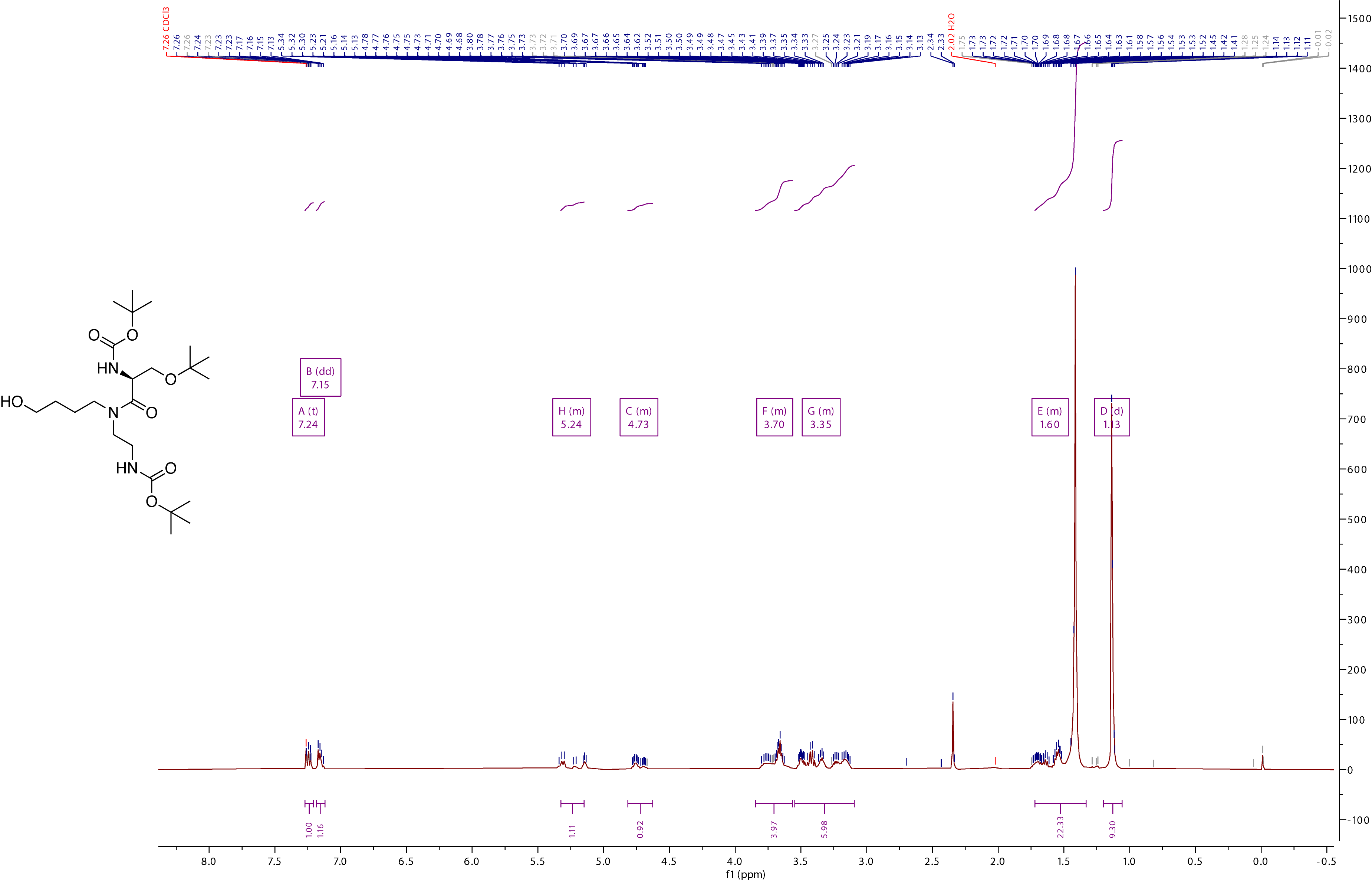


^13^C-NMR of compound 1f (4-carbon linker) in CDCl3 (126 MHz)


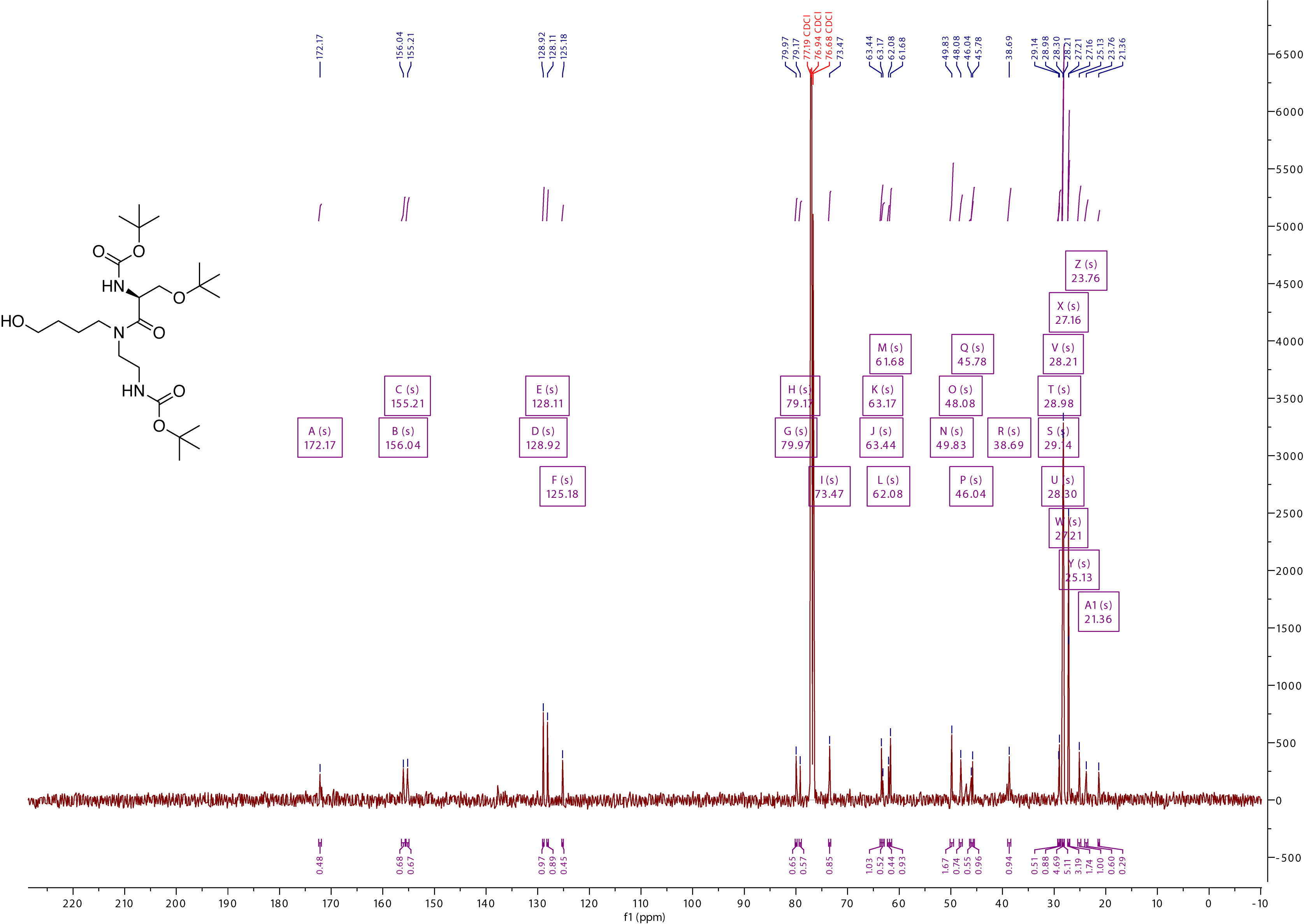


^1^H-NMR of compound TMP-lm in DMSO (500 MHz)


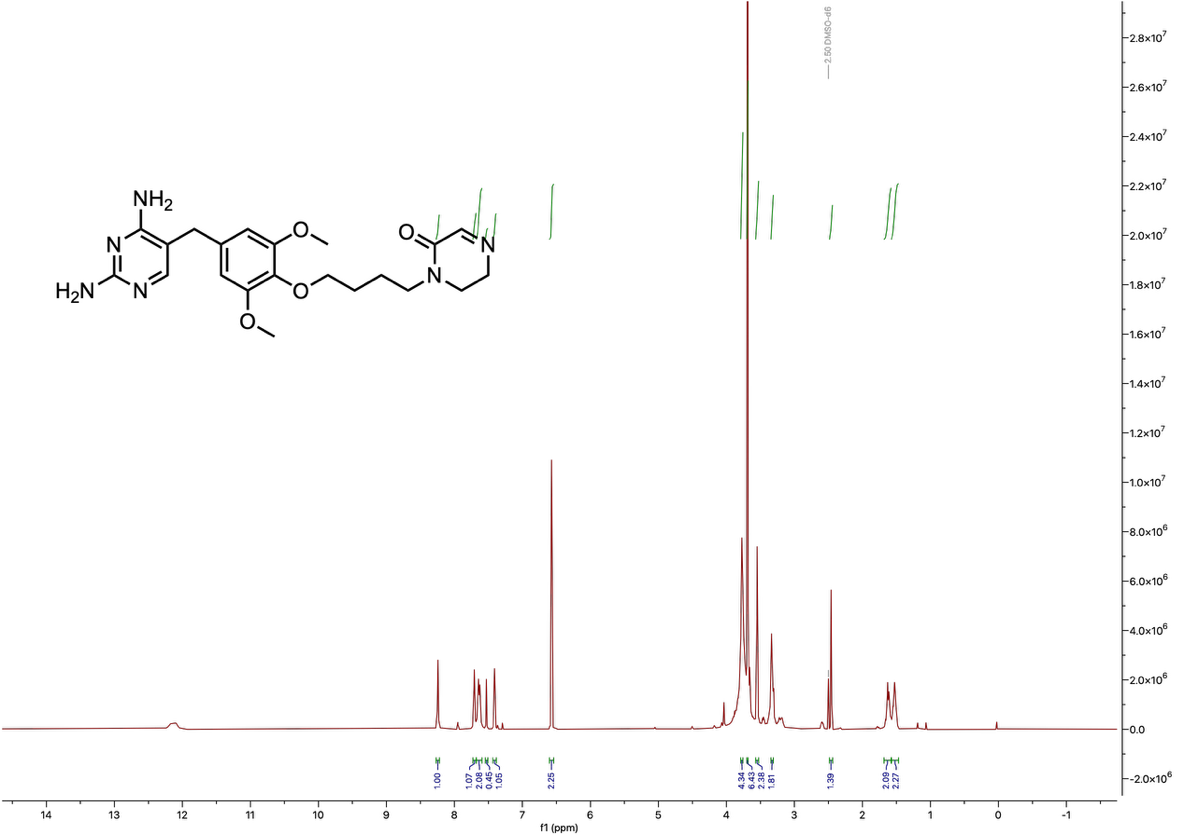


^13^C-NMR of compound TMP-lm in DMSO (500 MHz)


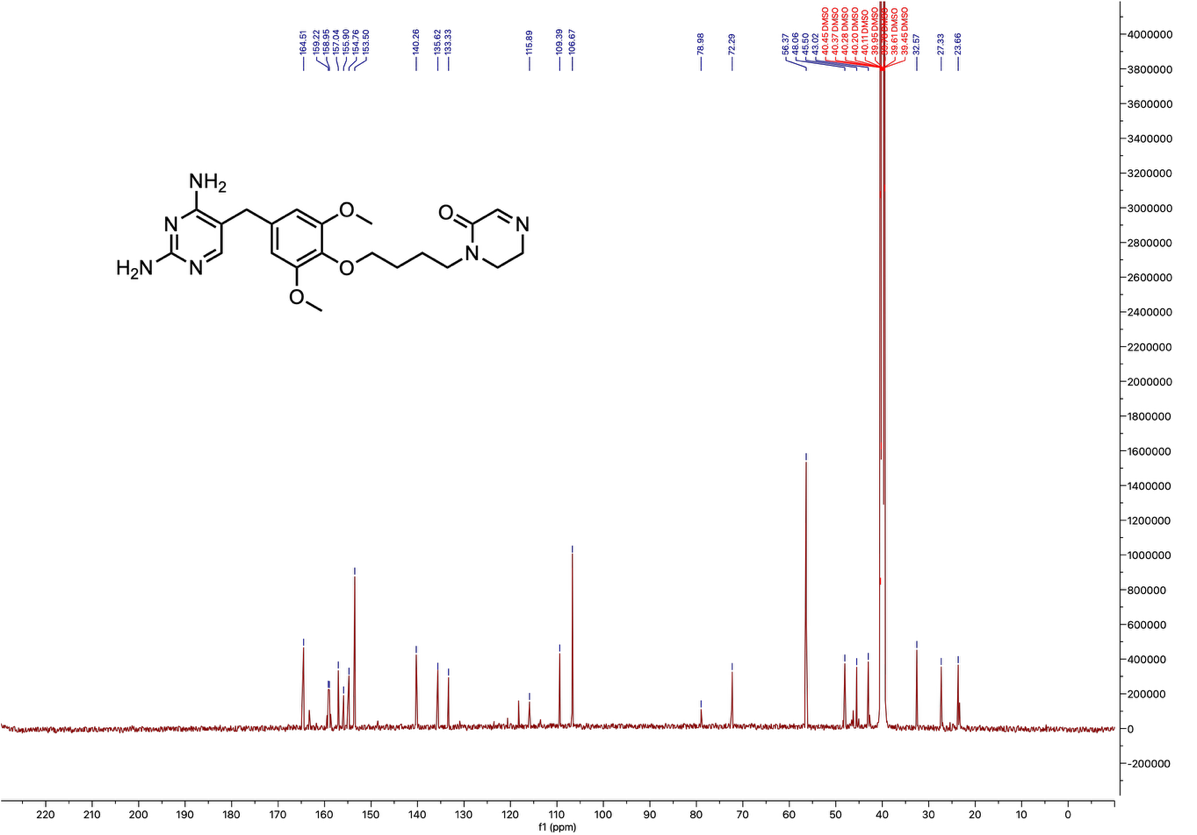


**9. Appendix 2: LC-MS**

LC-MS Spectrum of compound **P1aL**

**


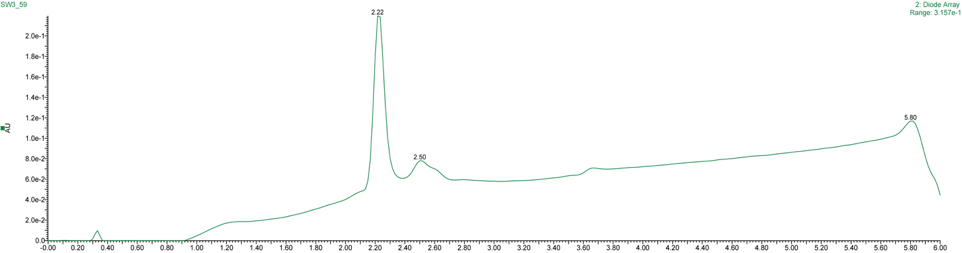


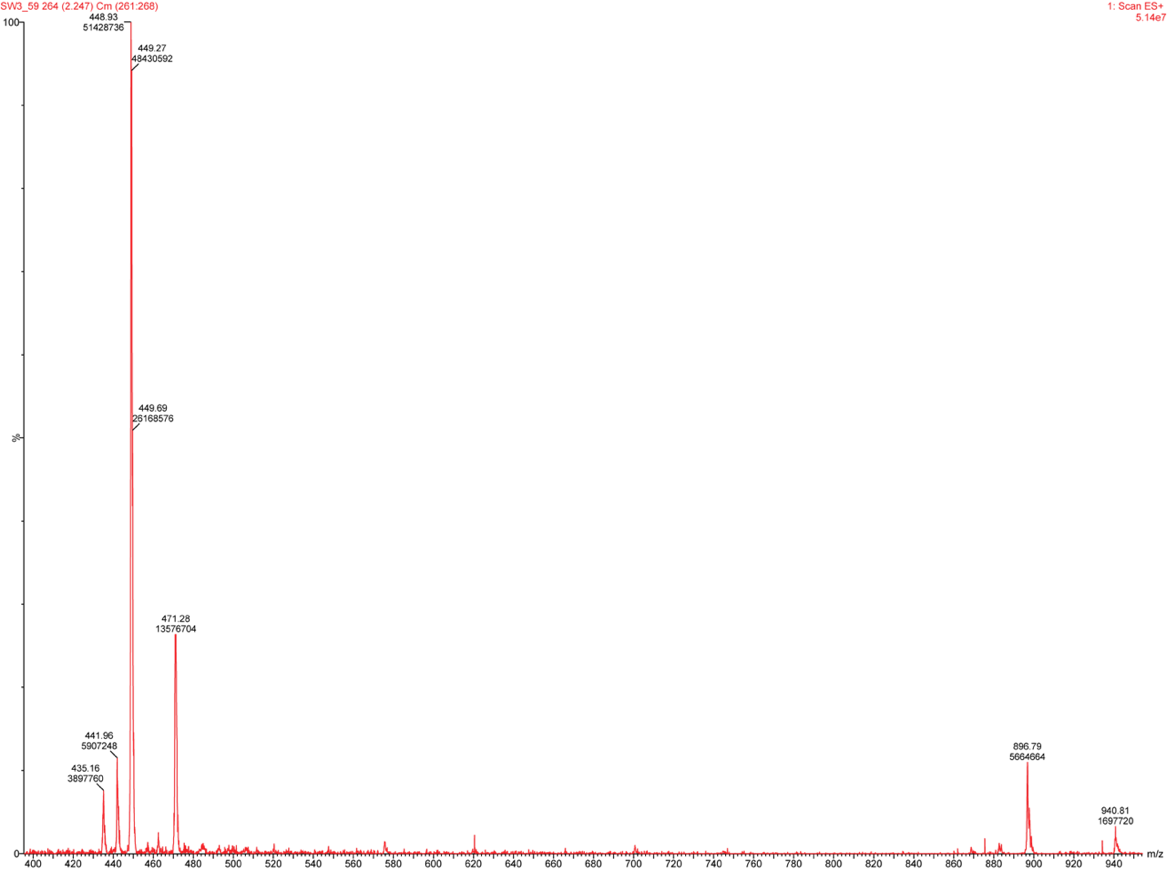


Characterization Data for compound **P1aL**. LC/MS top: UV traces bottom: low resolution mass spectrum. LR-ESI-MS: m/z calc. for [M+H]^+^: 896.5; found: 896.8, [M+2H]^2+^: 448.9.

LC-MS Spectrum of compound **P1a**

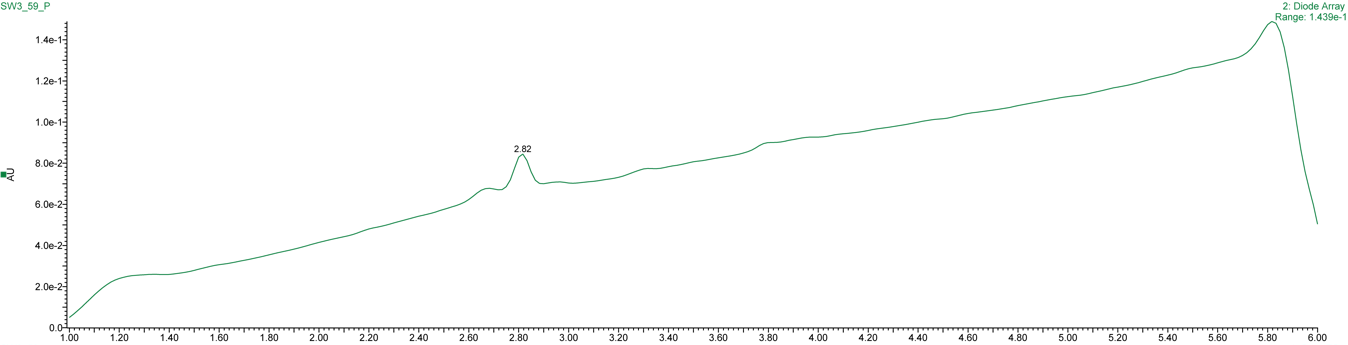

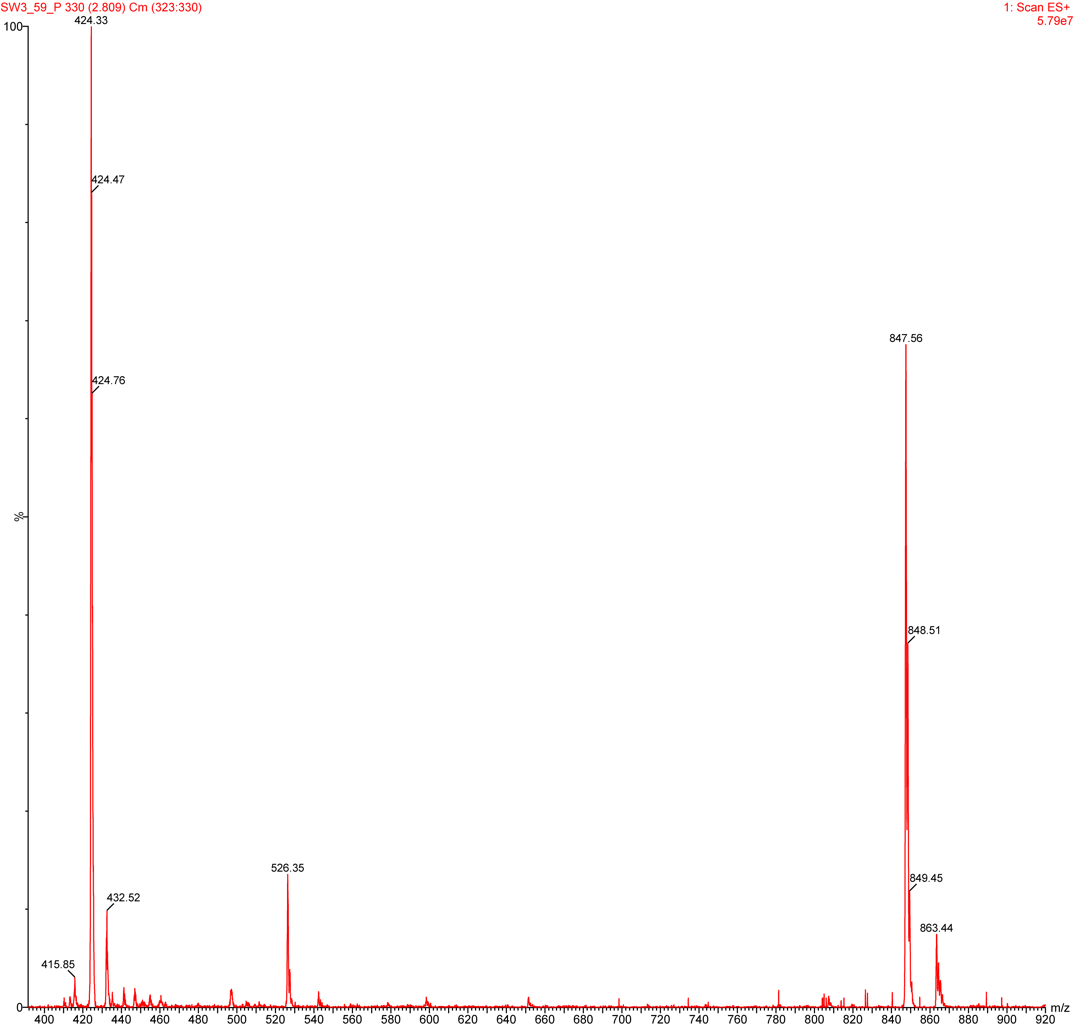


Characterization Data for **P1a** LC/MS top: UV traces bottom: low resolution mass spectrum. LR-ESI-MS: m/z calc. for [M+H]^+^: 847.5; found: 847.6, [M+2H]^2+^: 424.3.

LC-MS Spectrum of compound **P1bL**

**
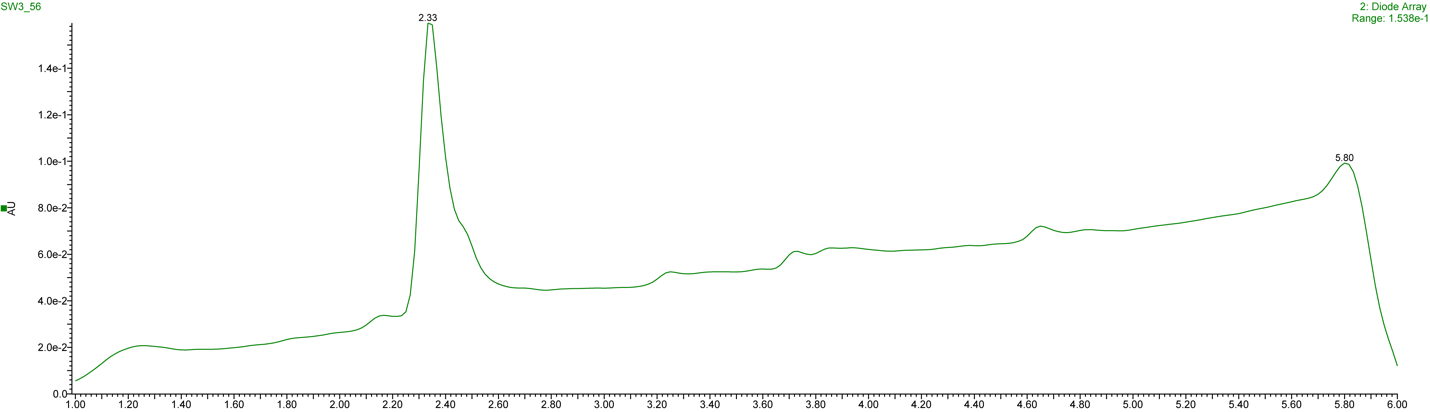

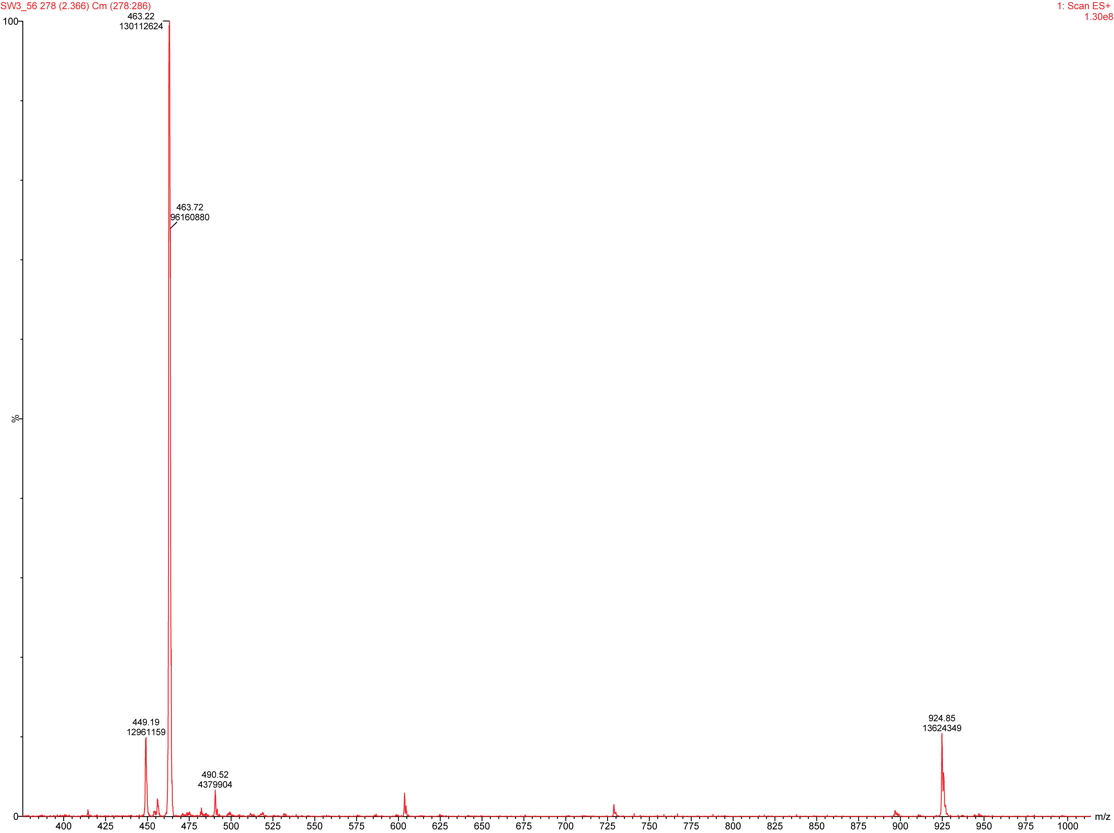


Characterization Data for compound **P1bL.** LC/MS top: UV traces bottom: low resolution mass spectrum. LR-ESI-MS: m/z calc. for [M+H]^+^: 924.6; found: 924.9, [M+2H]^2+^:463.2.

LC-MS Spectrum of compound **P1b**

**


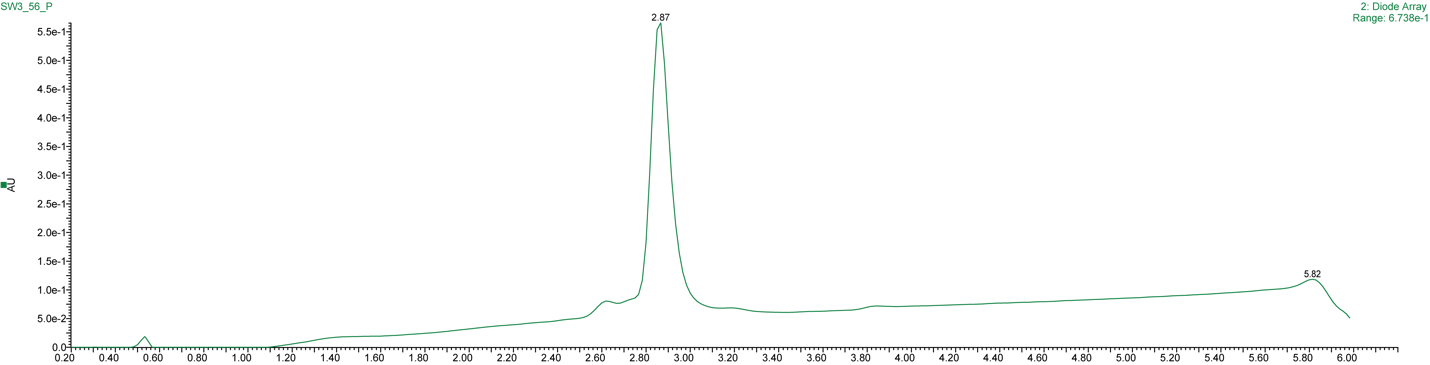

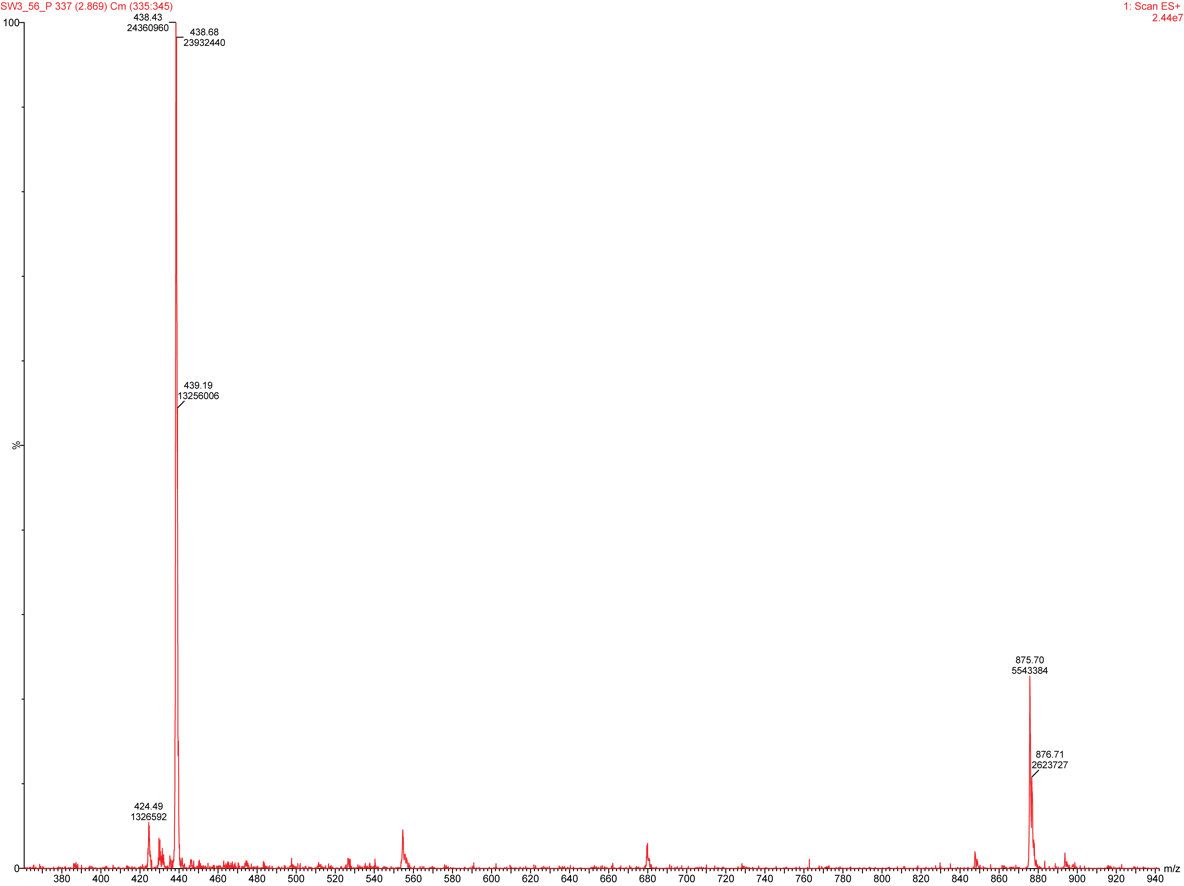


Characterization Data for **P1b**. LC/MS top: UV traces bottom: low resolution mass spectrum. LR-ESI-MS: m/z calc. for [M+H]^+^: 875.5; found [M+H]^+^: 875.7, [M+2H]^2+^: 438.4.

LC-MS Spectrum of compound **P1cL**

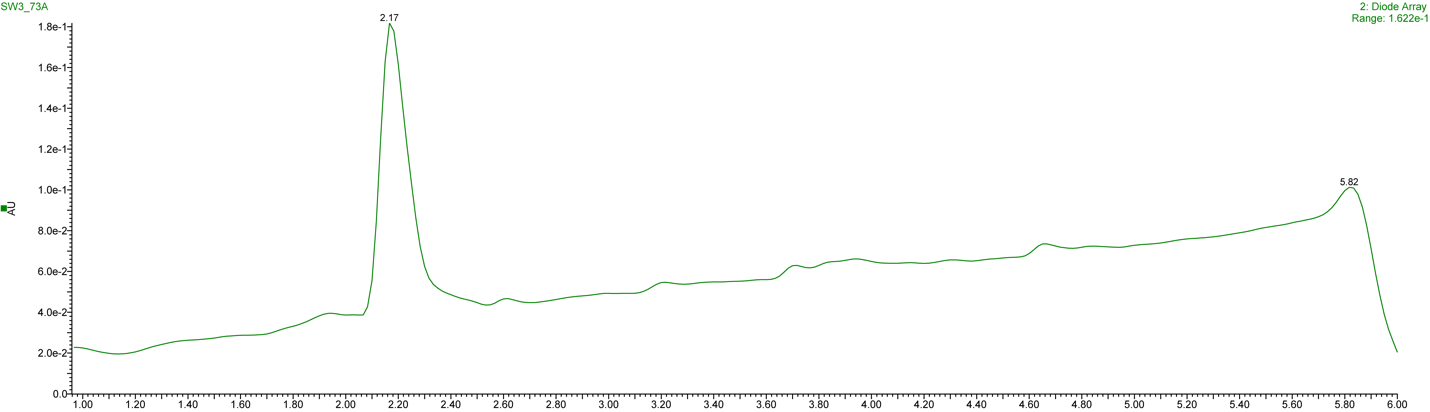


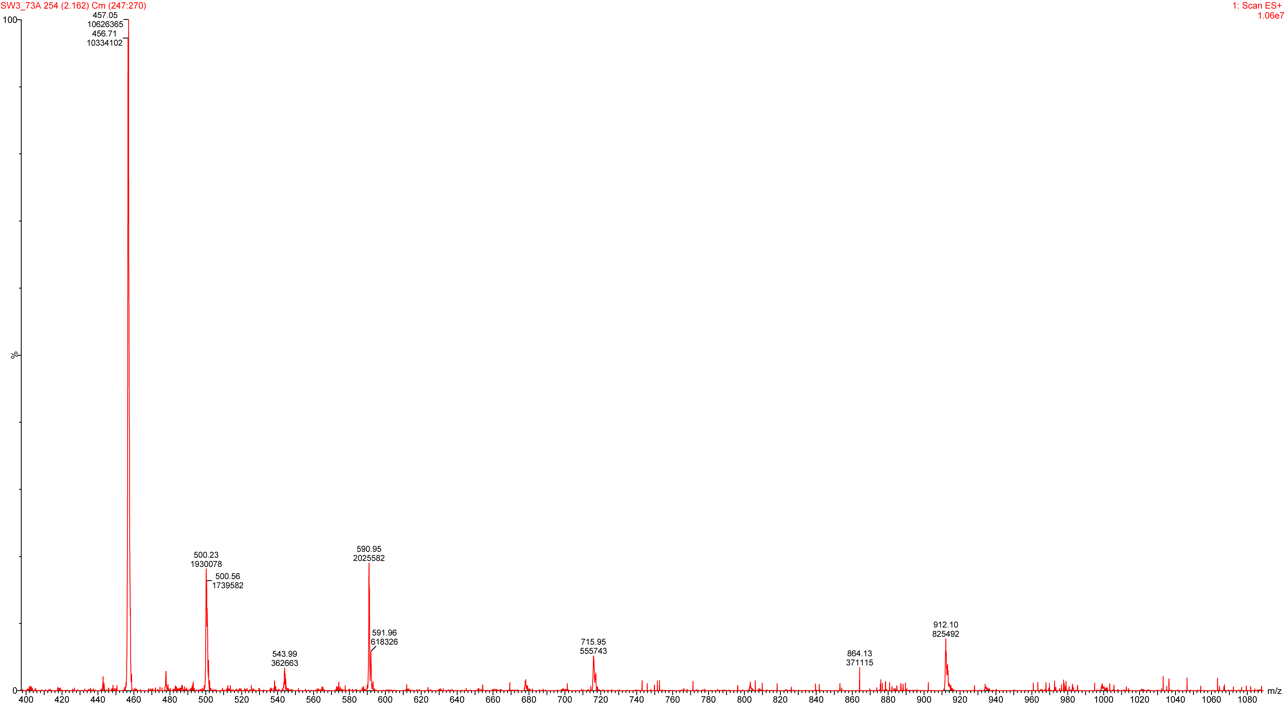


Characterization Data for compound **P1cL.** LC/MS top: UV traces bottom: low resolution mass spectrum. LR-ESI-MS: m/z calc. for [M+H]^+^: 911.5; found: 912., [M+2H]^2+^: 456.7.

LC-MS Spectrum of compound **P1c**

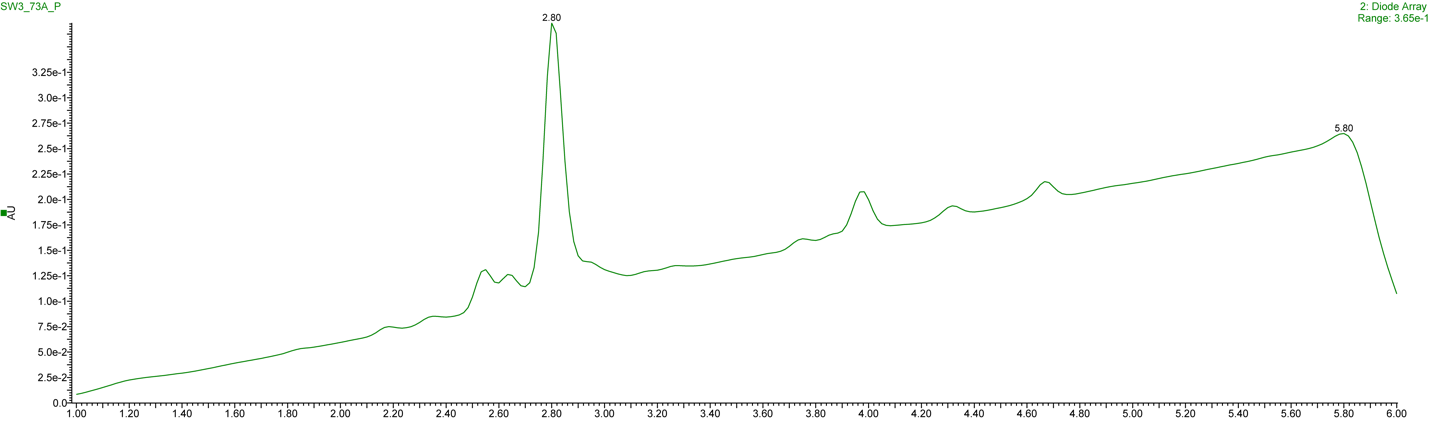


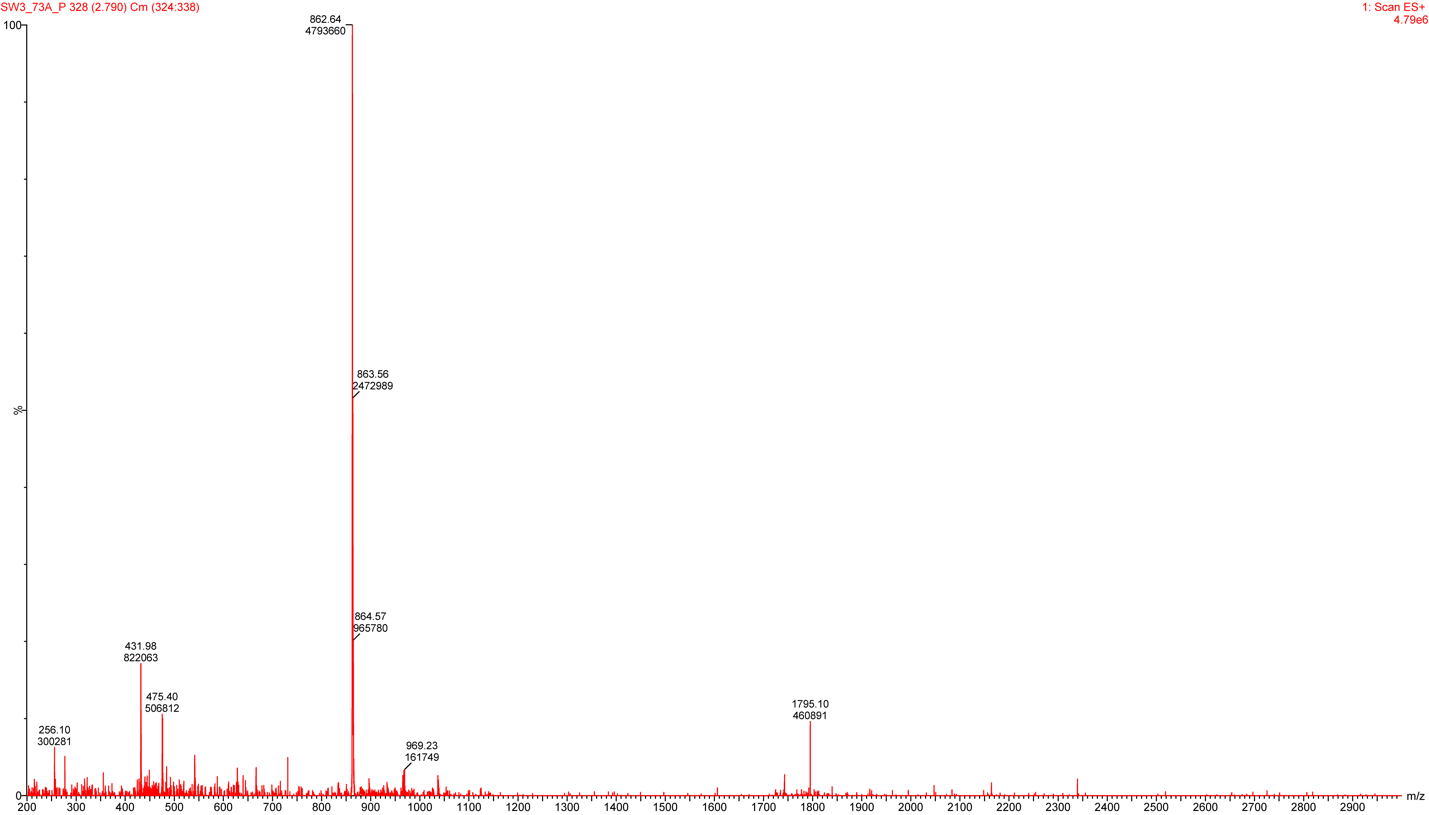


Characterization Data for compound **P1c.** LC/MS top: UV traces bottom: low resolution mass spectrum. LR-ESI-MS: m/z calc. for [M+H]^+^: 862.5; found: 862.6, [M+2H]^2+^: 432.0.

LC-MS Spectrum of compound **P1dL**

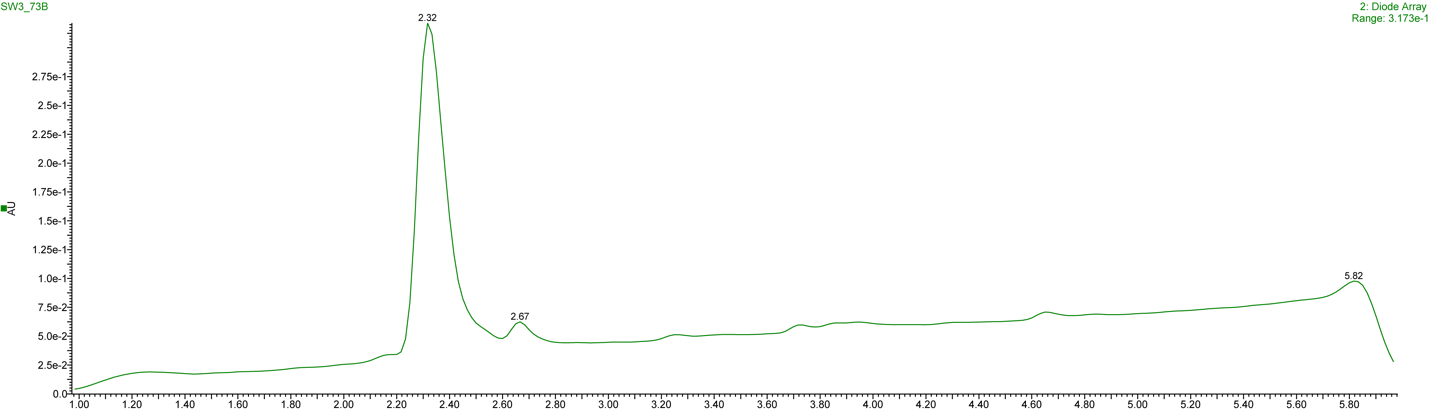


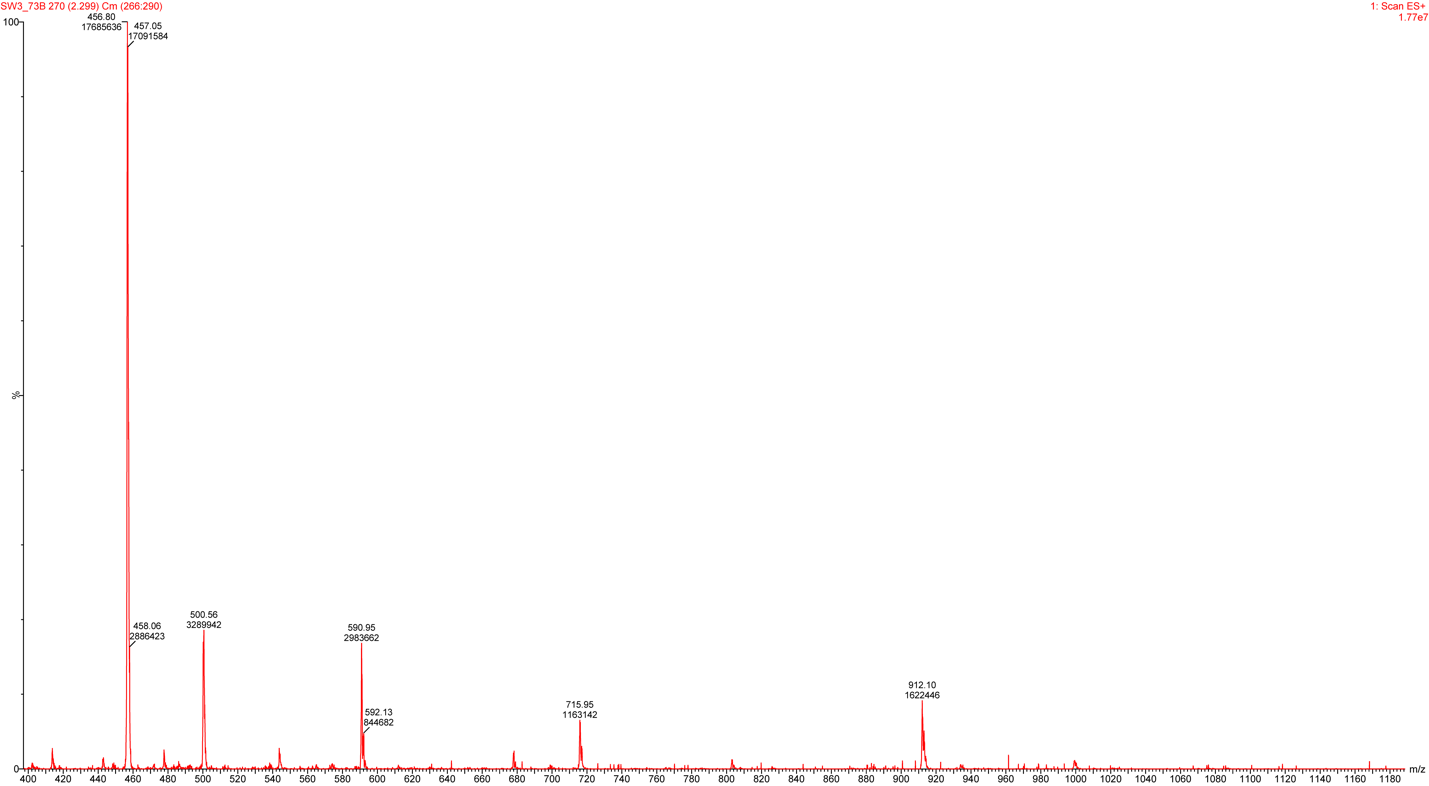


Characterization Data for compound **P1dL.** LC/MS top: UV traces bottom: low resolution mass spectrum. LR-ESI-MS: m/z calc. for [M+H]^+^: 911.5; found: 912.1, [M+2H]^2+^: 456.8.

LC-MS Spectrum of compound **P1d**

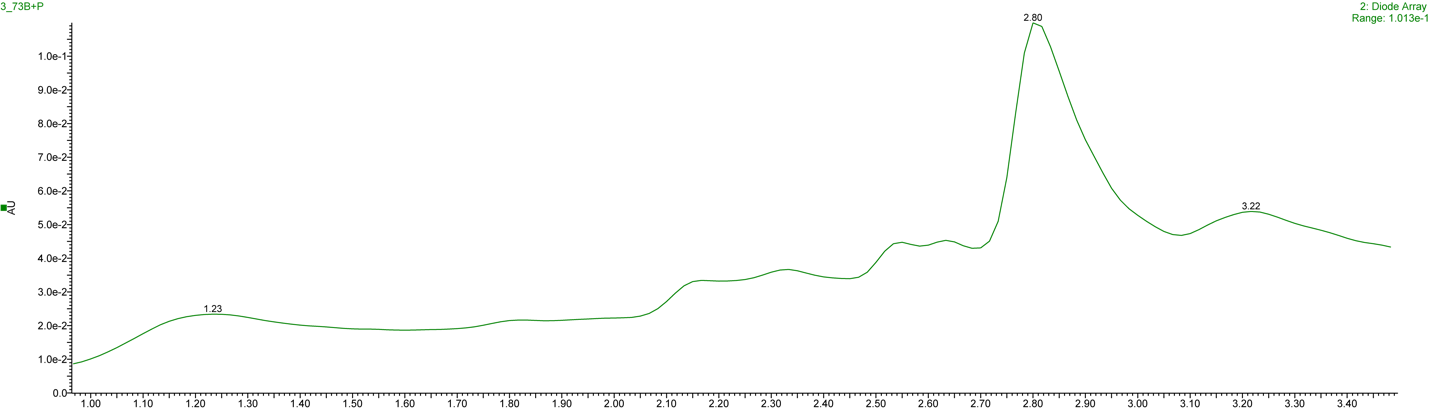


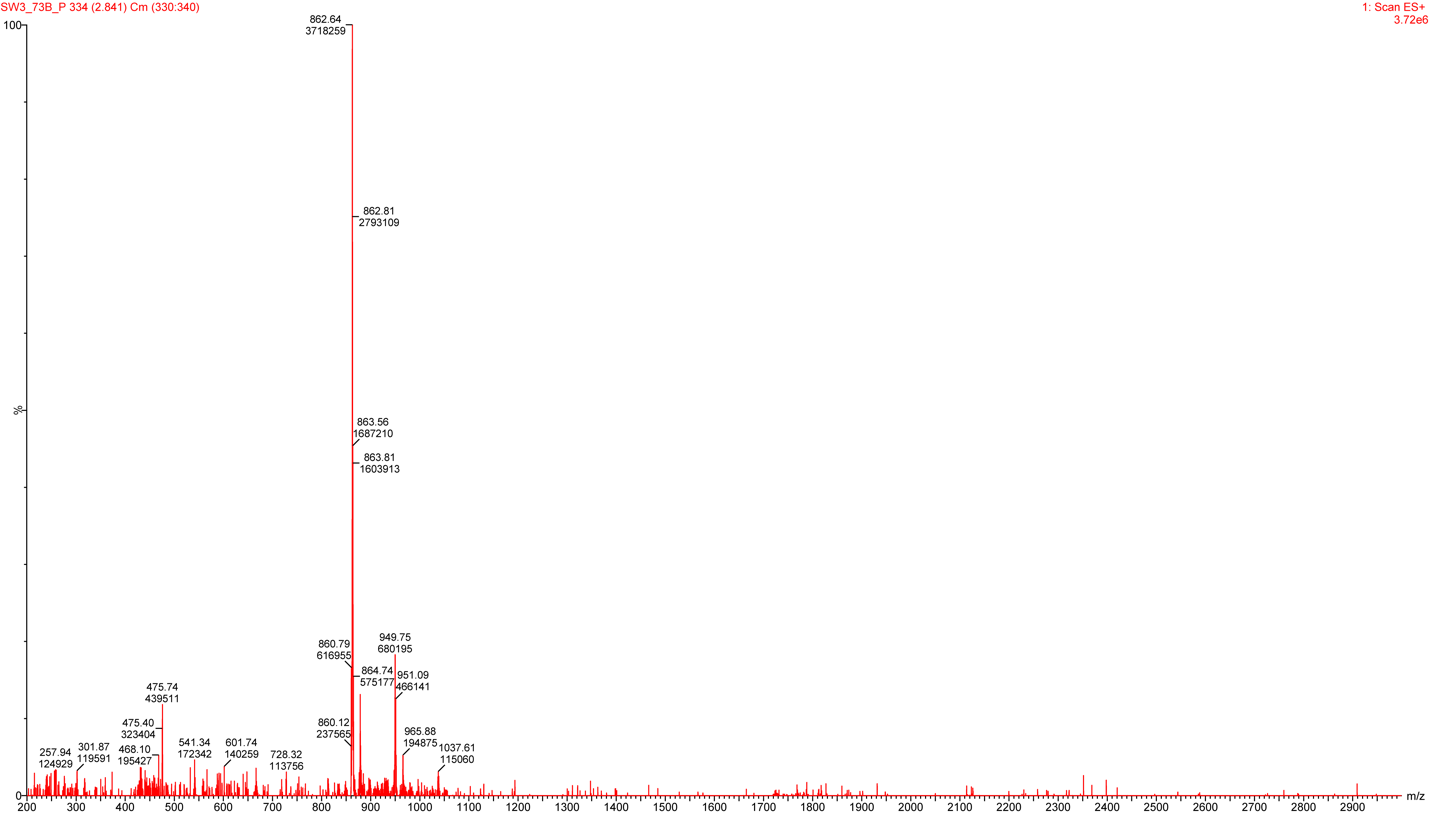


Characterization Data for compound **P1d**. LC/MS top: UV traces bottom: low resolution mass spectrum. LR-ESI-MS: m/z calc. for [M+H]^+^: 862.5; found: 862.6.

LC-MS Spectrum of compound **P1eL**

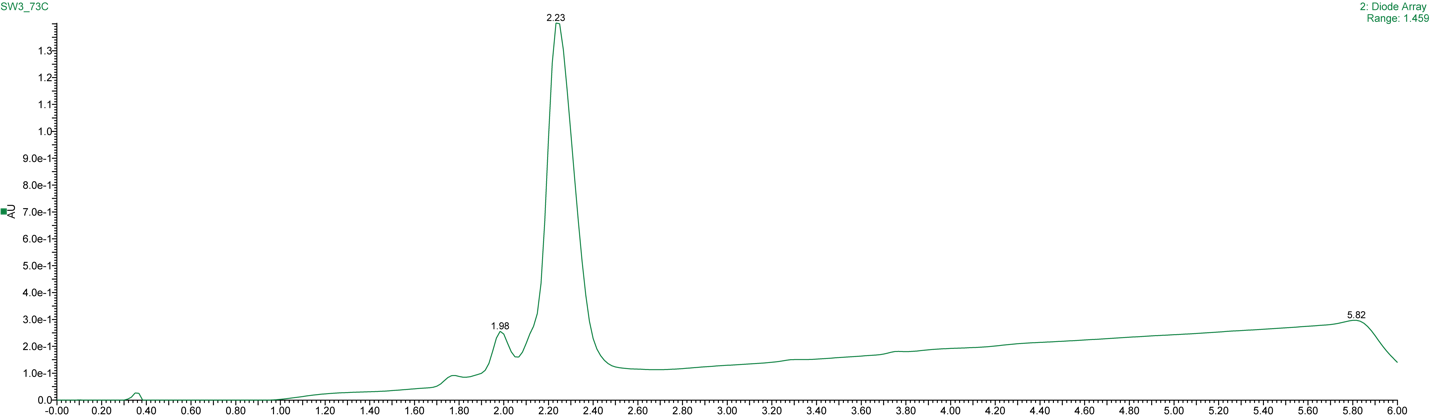


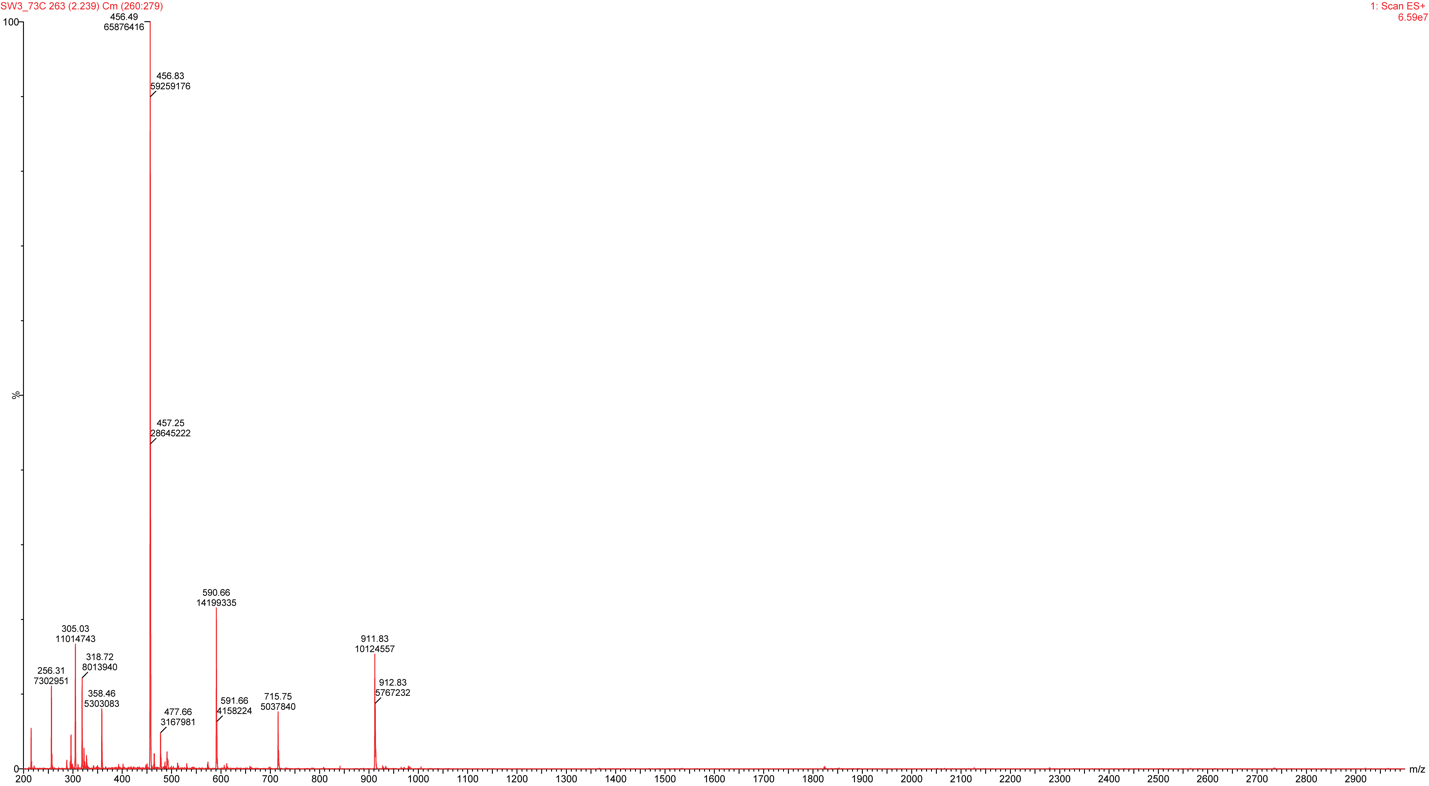


Characterization Data for compound **P1eL**. LC/MS top: UV traces bottom: low resolution mass spectrum. LR-ESI-MS: m/z calc. for [M+H]^+^: 911.5; found: 911., [M+2H]^2+^: 456.5.

LC-MS Spectrum of compound **P1e**

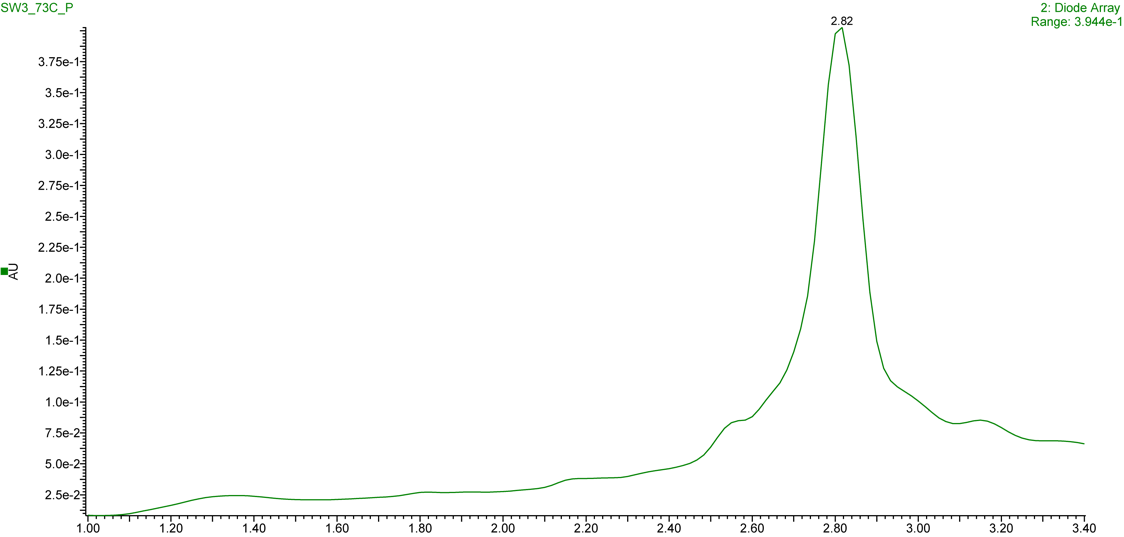


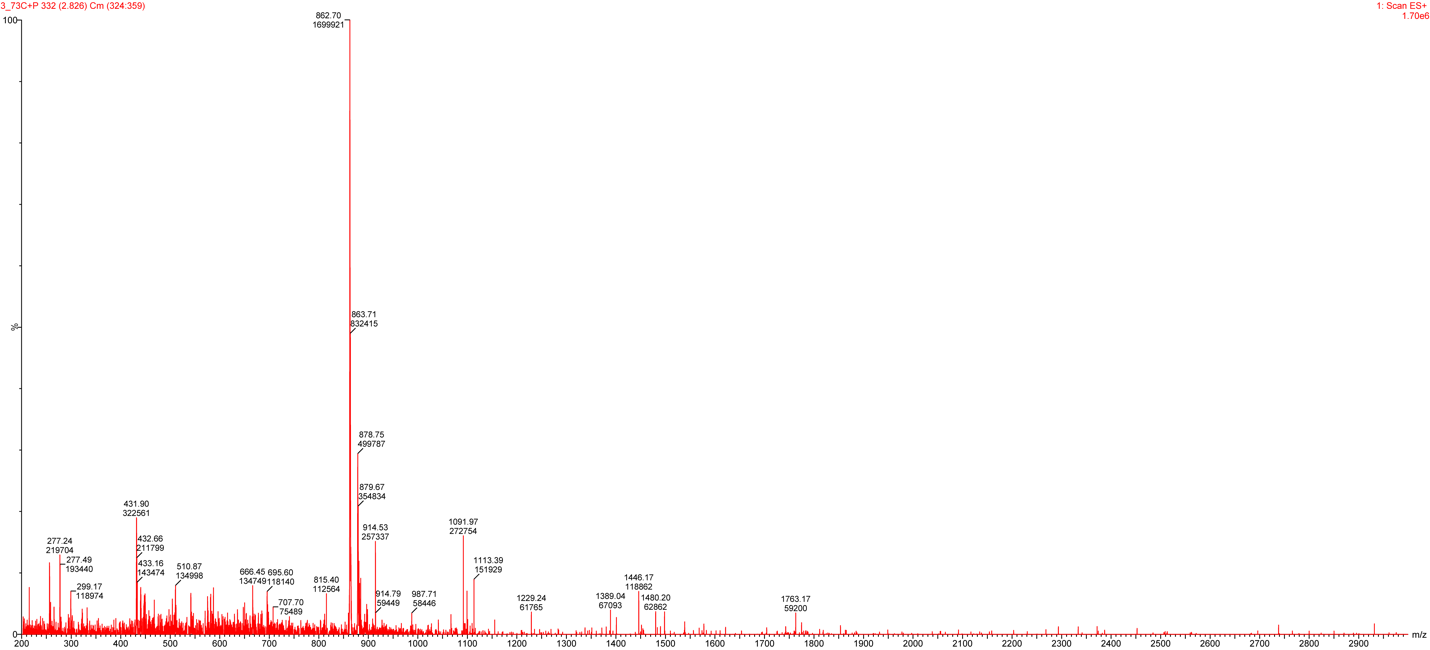


Characterization Data for compound **P1e**. LC/MS top: UV traces bottom: low resolution mass spectrum. LR-ESI-MS: m/z calc. for [M+H]^+^: 862.5; found: 862.7 g/mol, [M+2H]^2+^: 431.9.

LC-MS Spectrum of compound **P1fL**

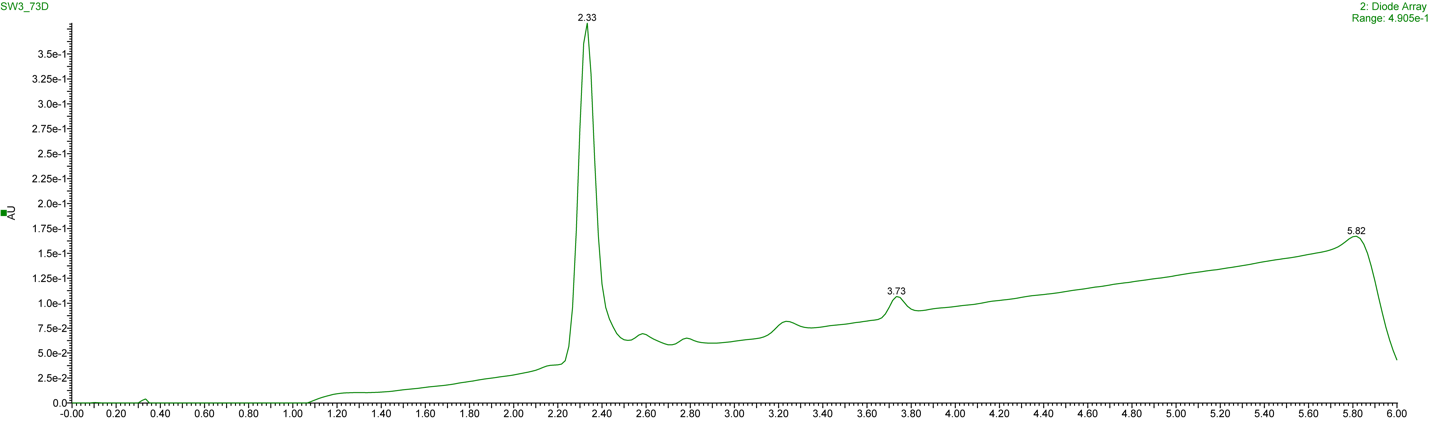


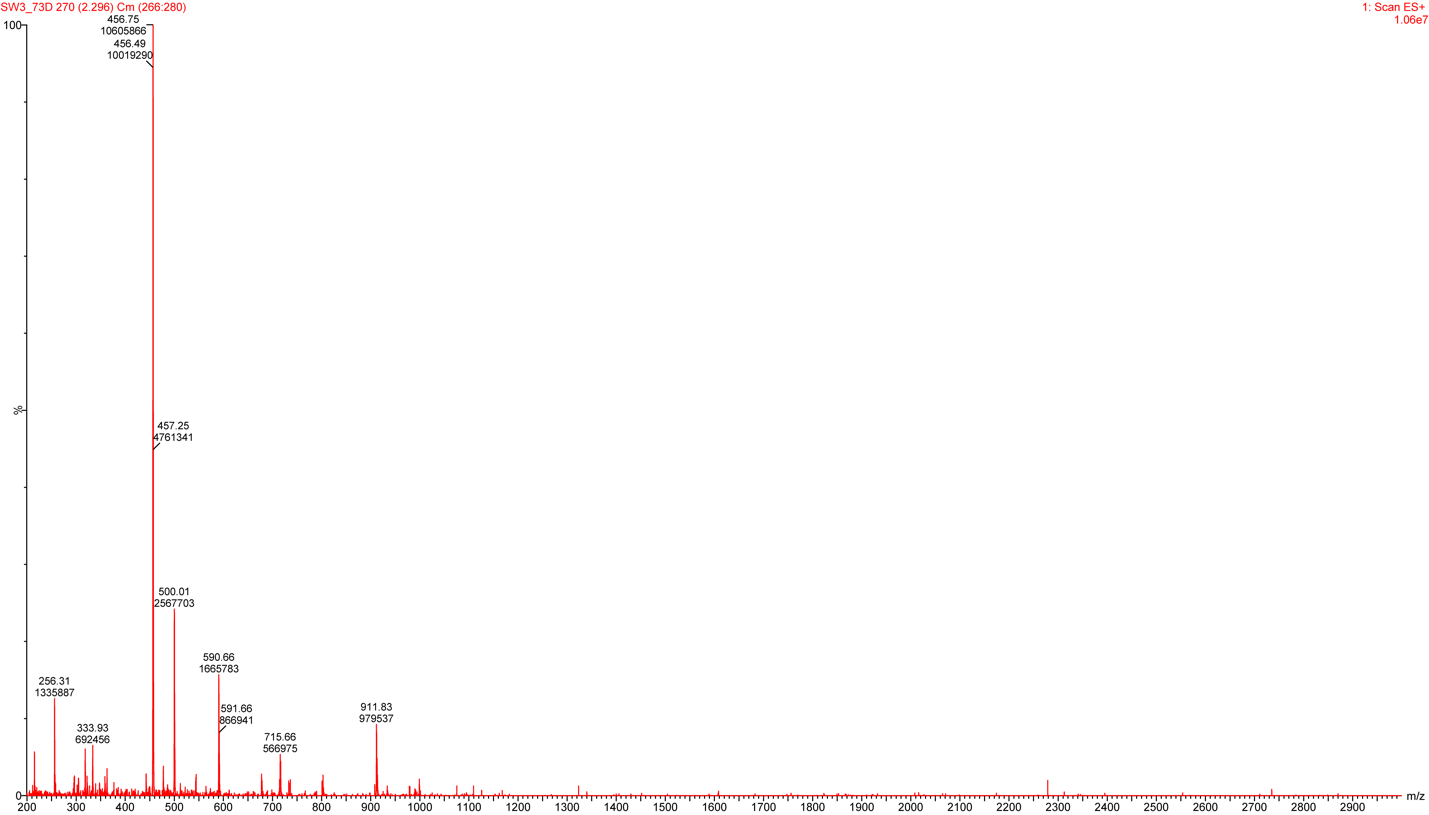


Characterization Data for compound **P1fL**. LC/MS top: UV trace bottom: low resolution mass spectrum. LR-ESI-MS: m/z calc. for [M+H]^+^: 911.5; found : 911.8, [M+2H]^2+^: 456.8.

LC-MS Spectrum of compound **P1f**

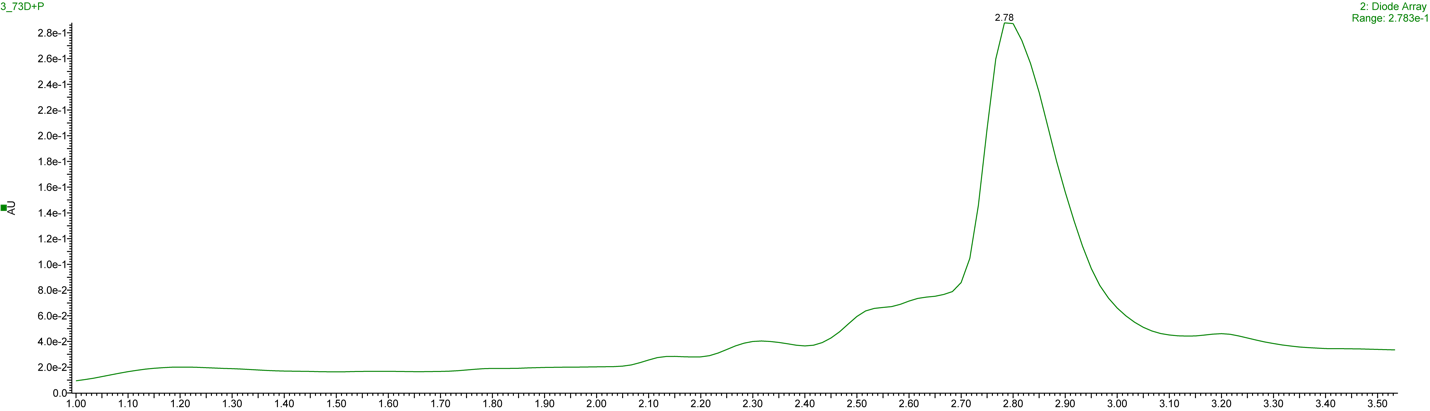


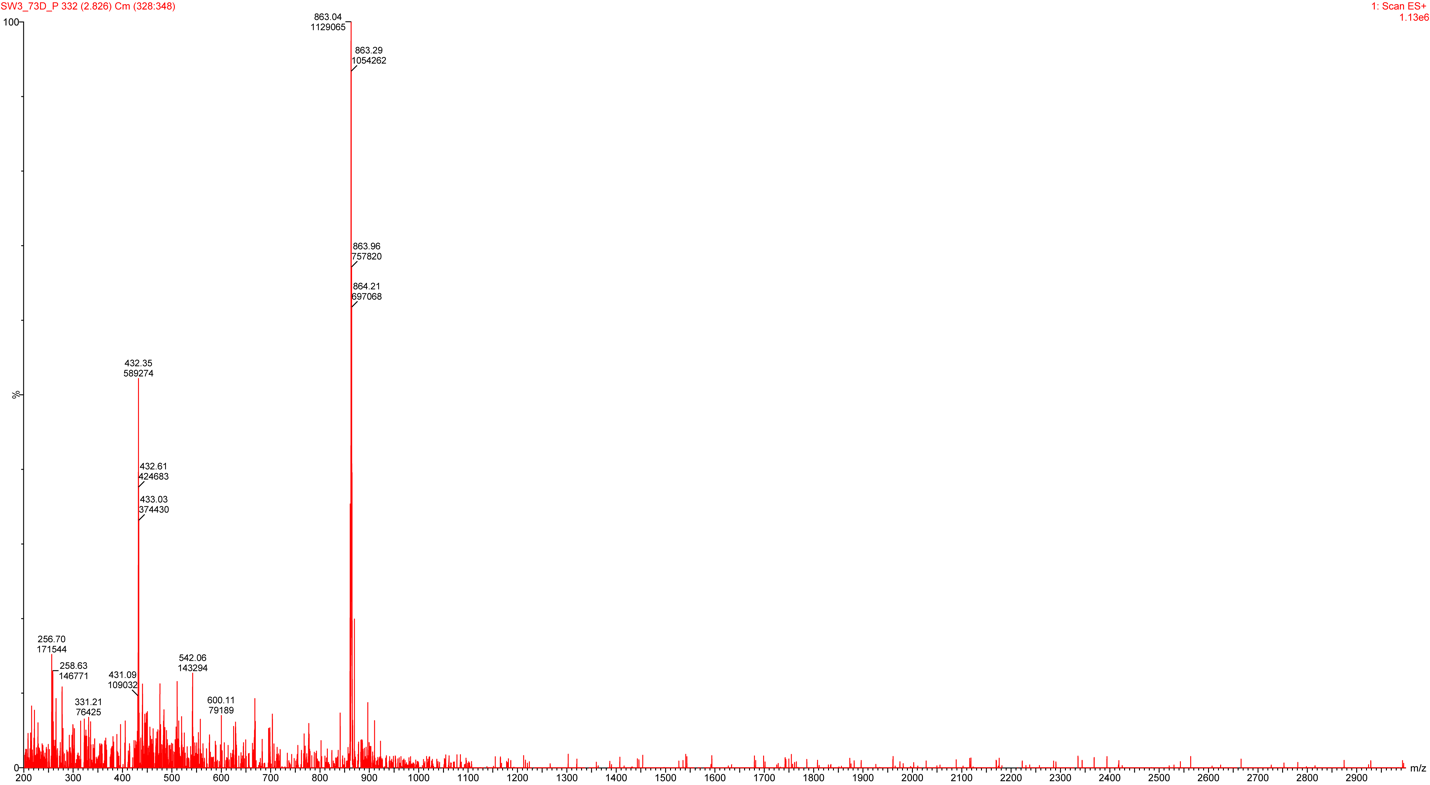


Characterization Data for compound **P1f.** LC/MS top: UV traces bottom: low resolution mass spectrum. LR-ESI-MS: m/z calc. for [M+H]^+^: 862.5; found: 863.0, [M+2H]^2+^: 432.4.

LC-MS Spectrum of compound **P3aL**

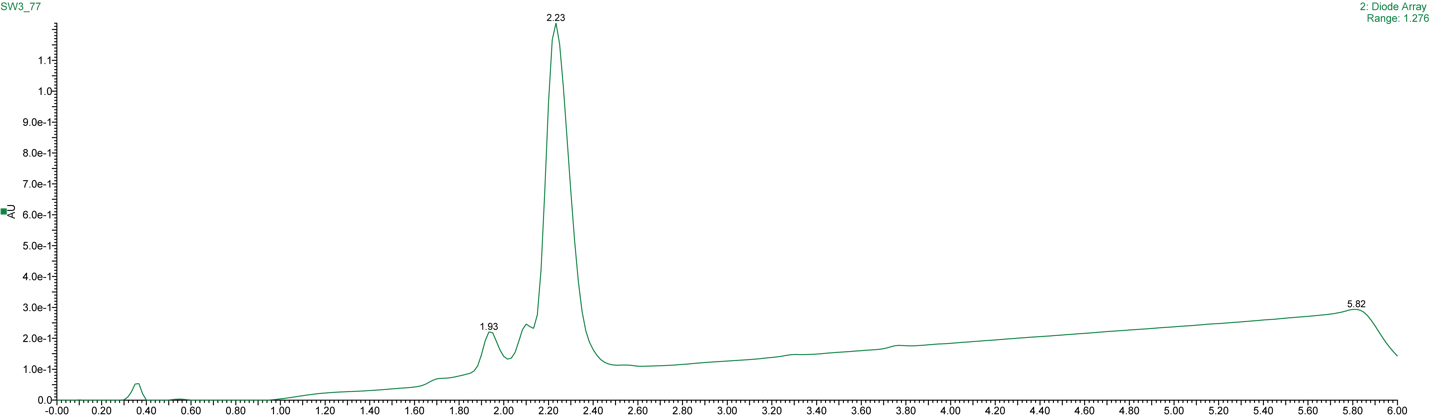


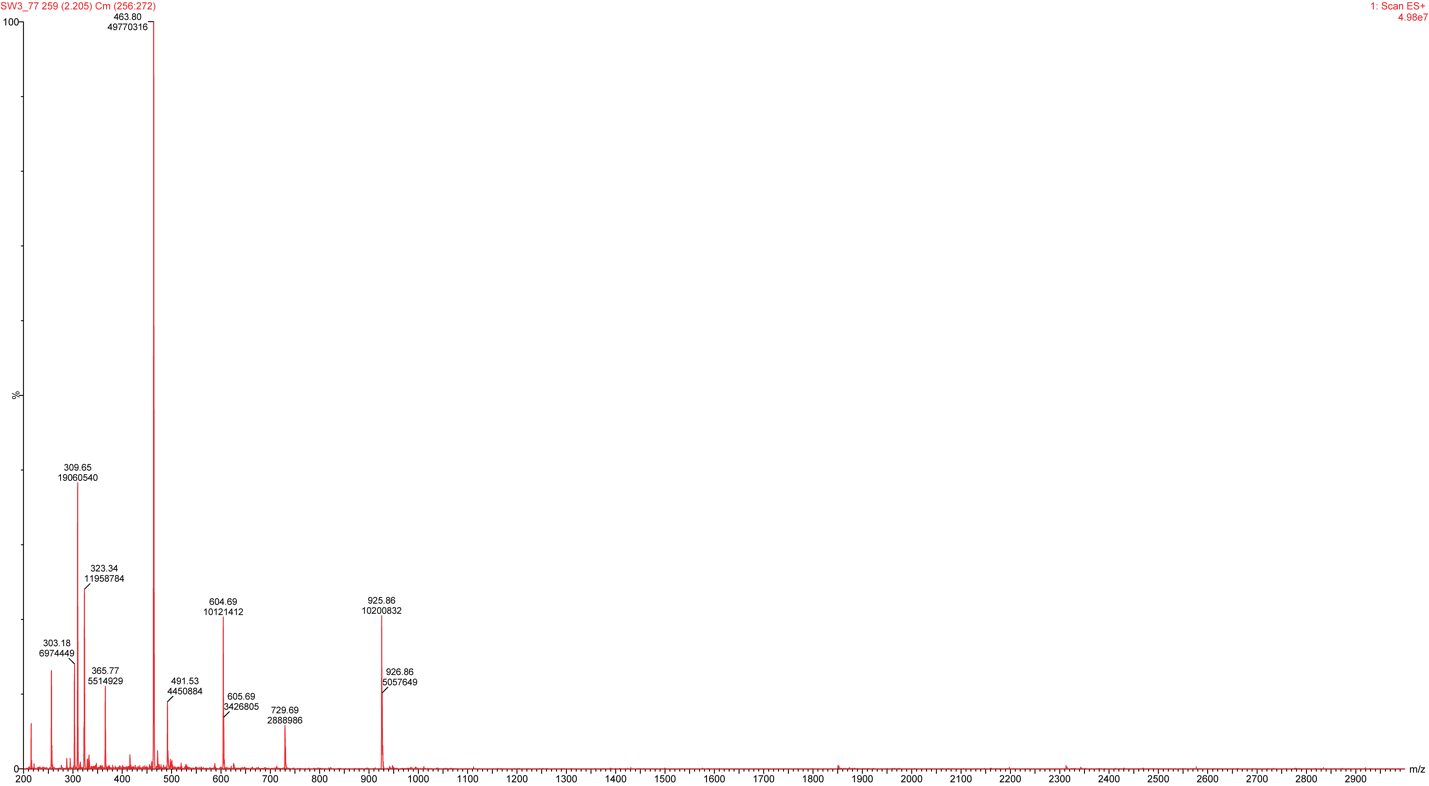


Characterization Data for compound **P3aL**. LC/MS top: UV traces bottom: low resolution mass spectrum. LR-ESI-MS: m/z calc. for [M+H]^+^: 925.5; found: 925.9, [M+2H]^2+^: 463.8, [M+3H]^3+^: 309.7.

LC-MS Spectrum of compound **P3a**

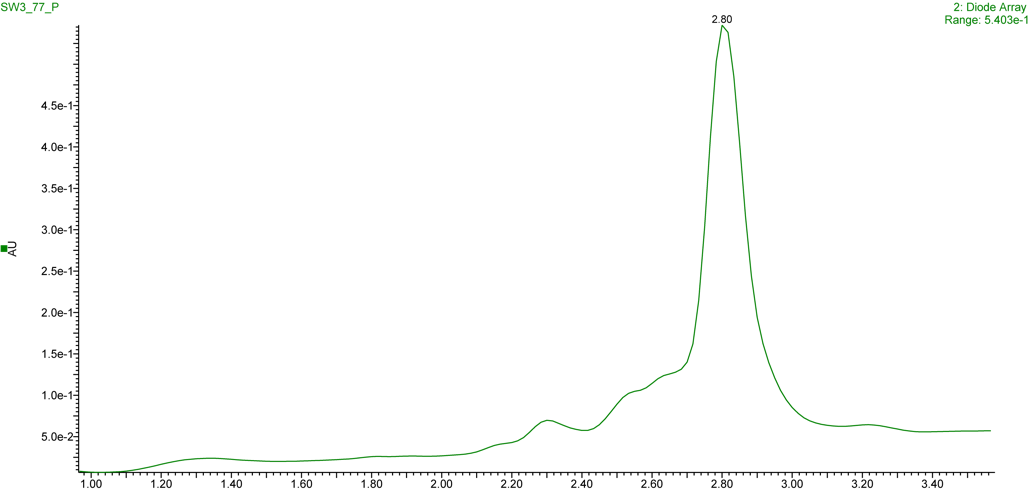


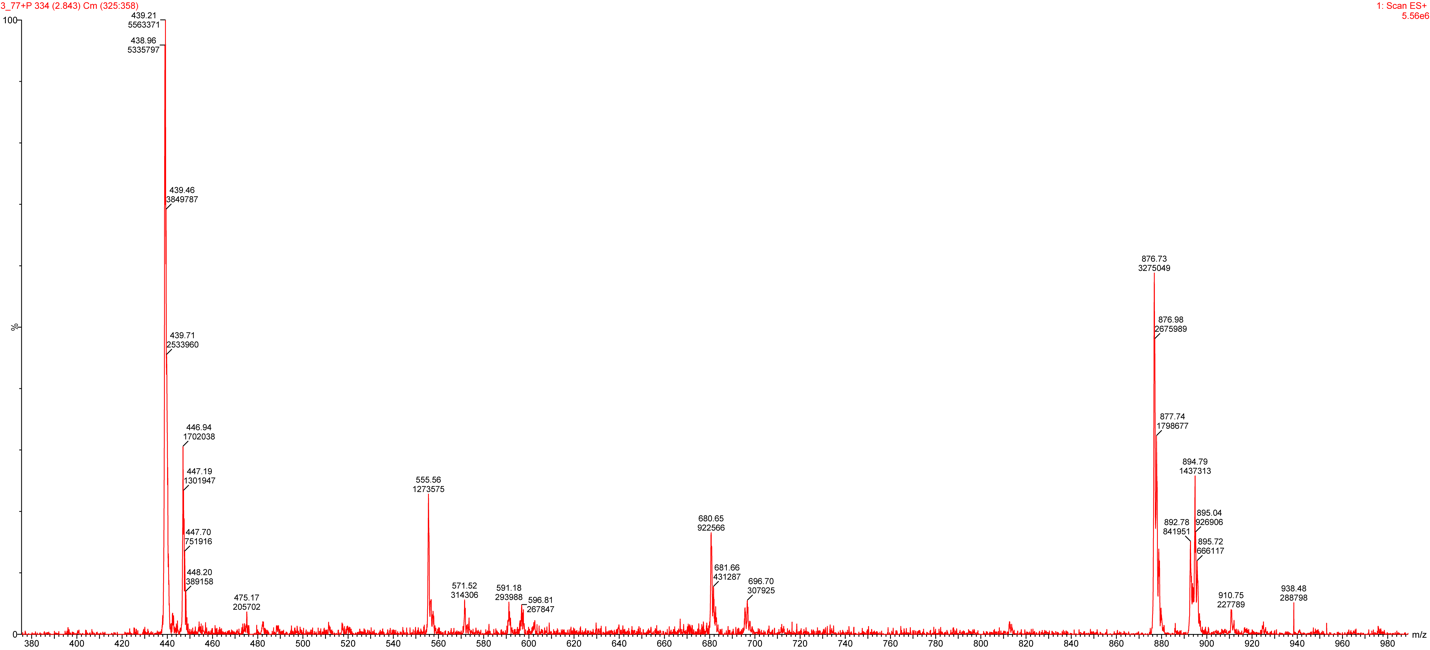


Characterization Data for compound **P3a**. LC/MS top: UV traces bottom: low resolution mass spectrum. LR-ESI-MS: m/z calc. for [M]^+^: 876.5; found: 876.7, [M+H_2_O]^+^ 894.8, [M+H]^2+^: 439.2, [M+H_2_O+H]^2+^ 446.9.

LC-MS Spectrum of compound **P3bL**

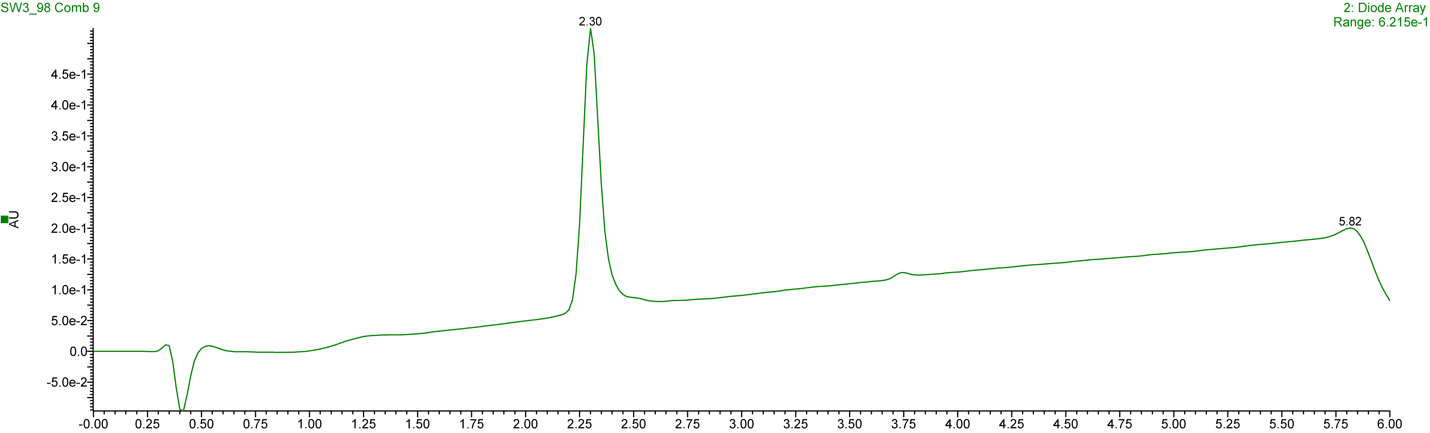

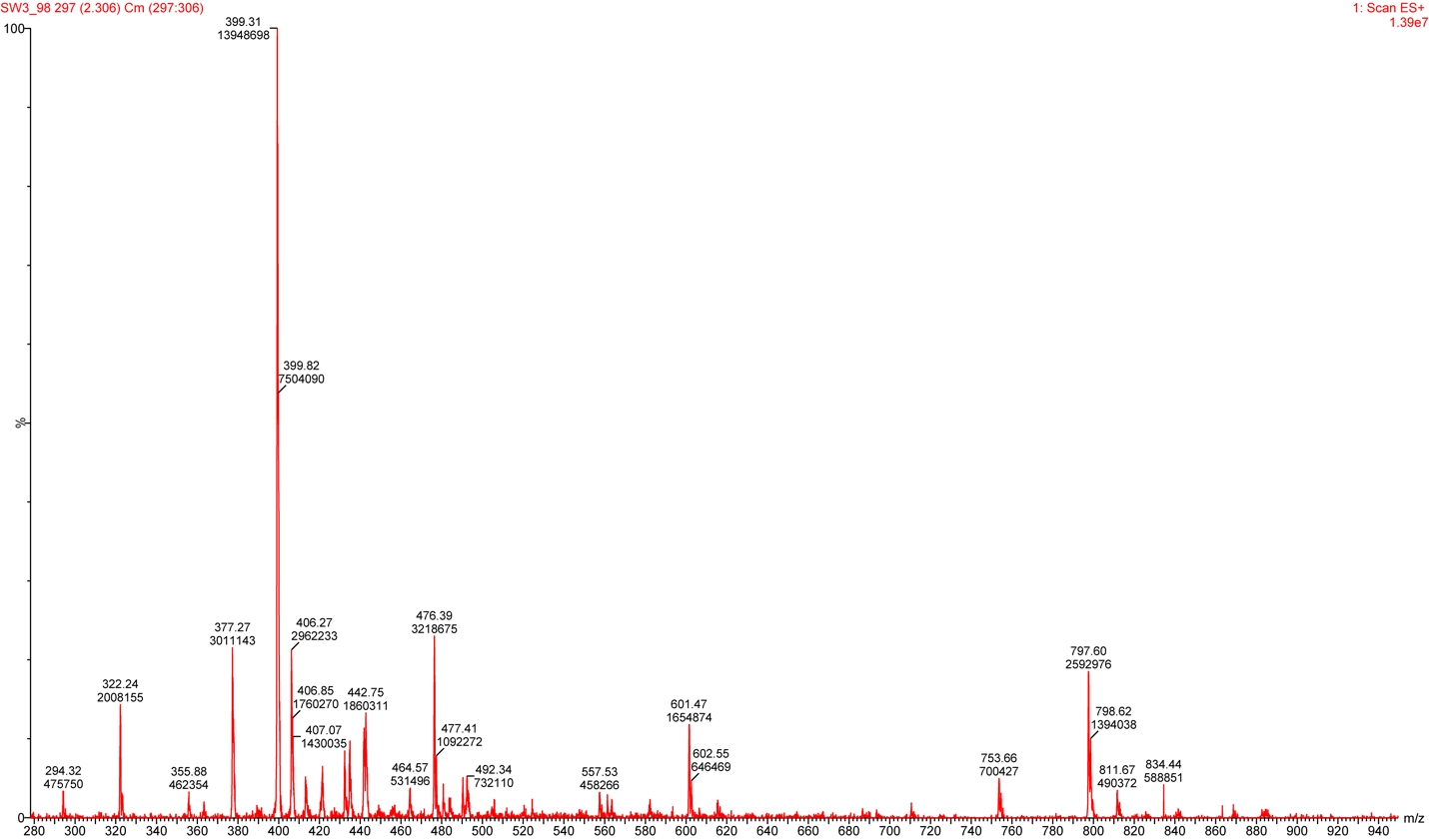


Characterization Data for compound **P3bL**. LC/MS top: UV traces bottom: low resolution mass spectrum. LR-ESI-MS: m/z calc. for [M+H]^+^.: 797.5; found : 797.6, [M+2H]^2+^: 399.3.

LC-MS Spectrum of compound **P1bPL**

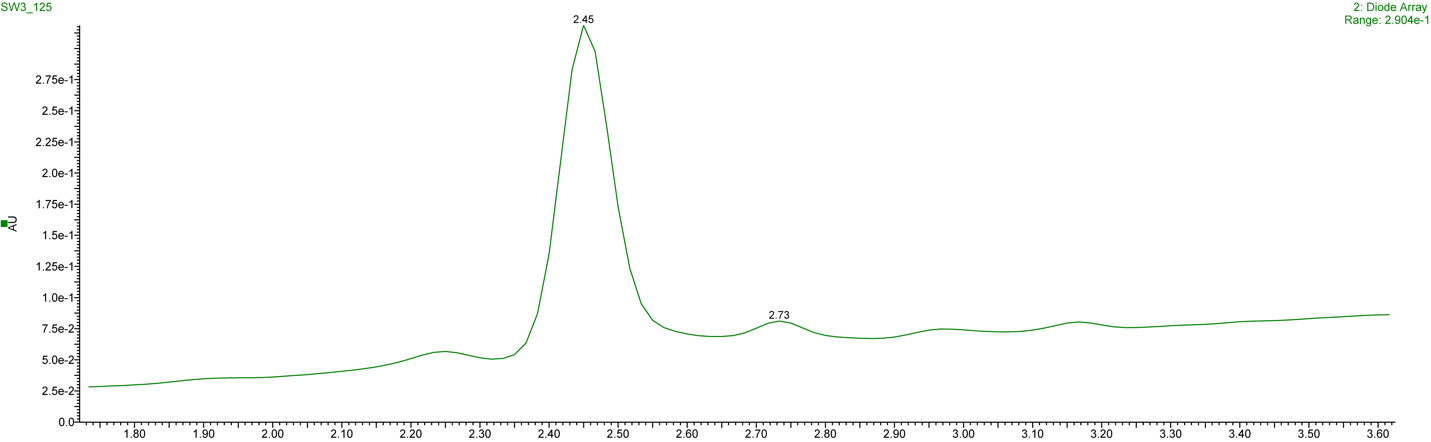


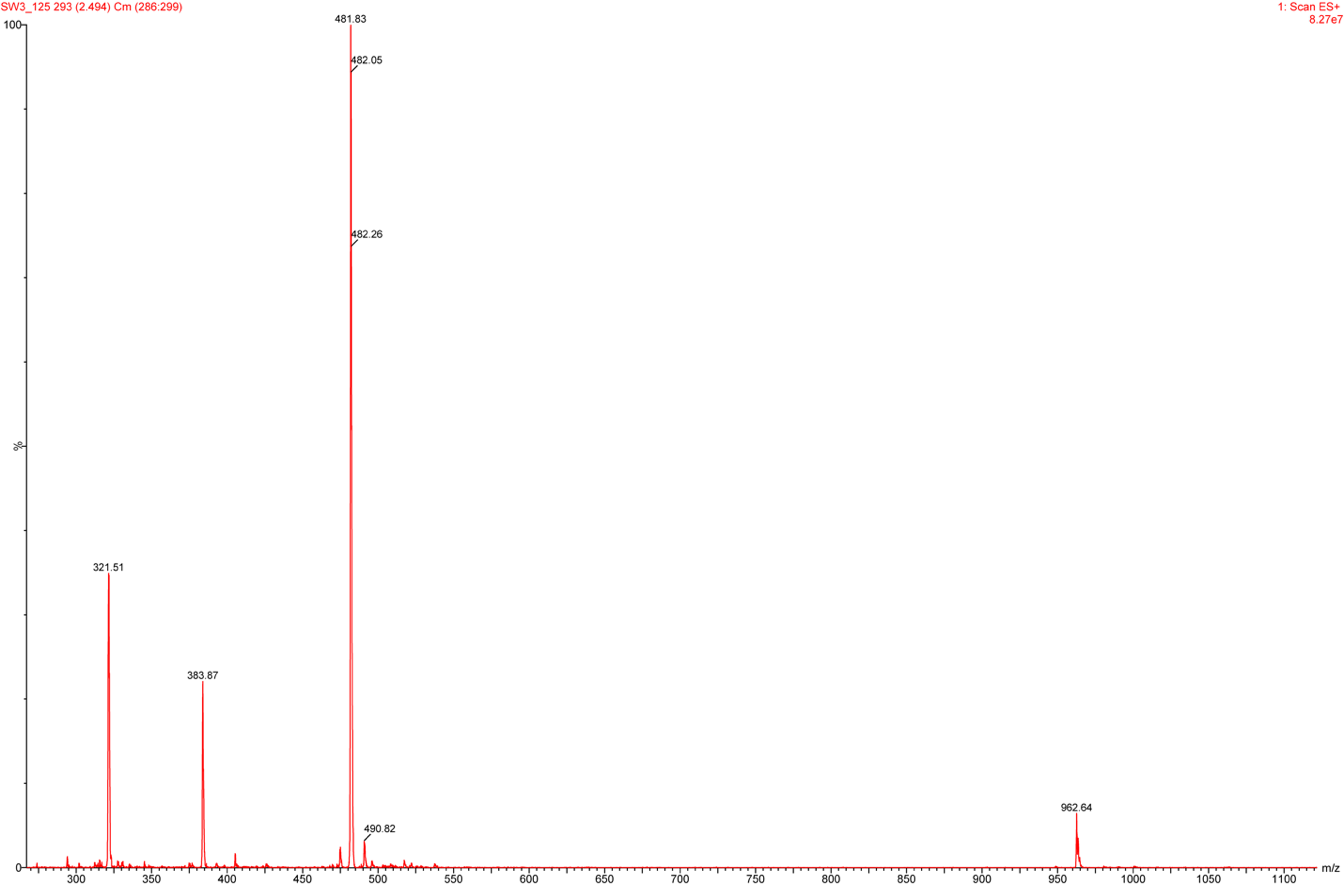


Characterization Data for compound **P1bPL**. LC/MS top: UV traces bottom: low resolution mass spectrum. LR-ESI-MS: m/z calc. for [M+H]^+^.: 962.6; found: 962.6, [M+2H]^2+^: 481.8

LC-MS Spectrum of compound **P1bP**

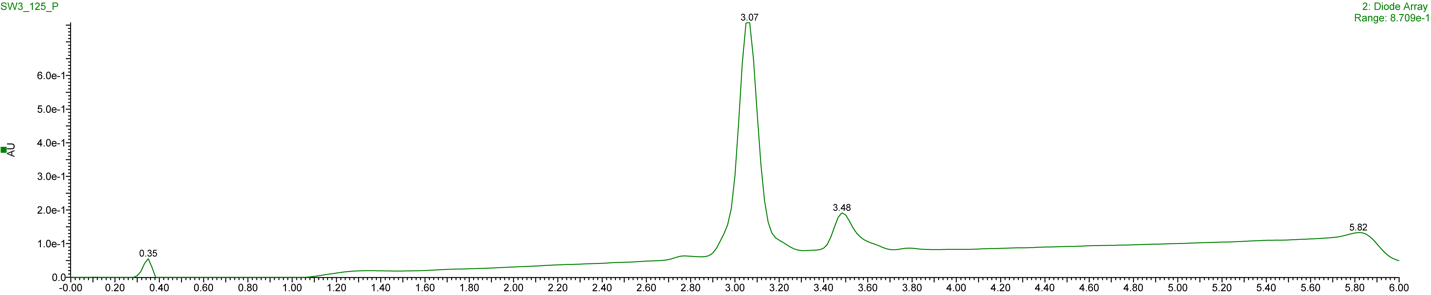


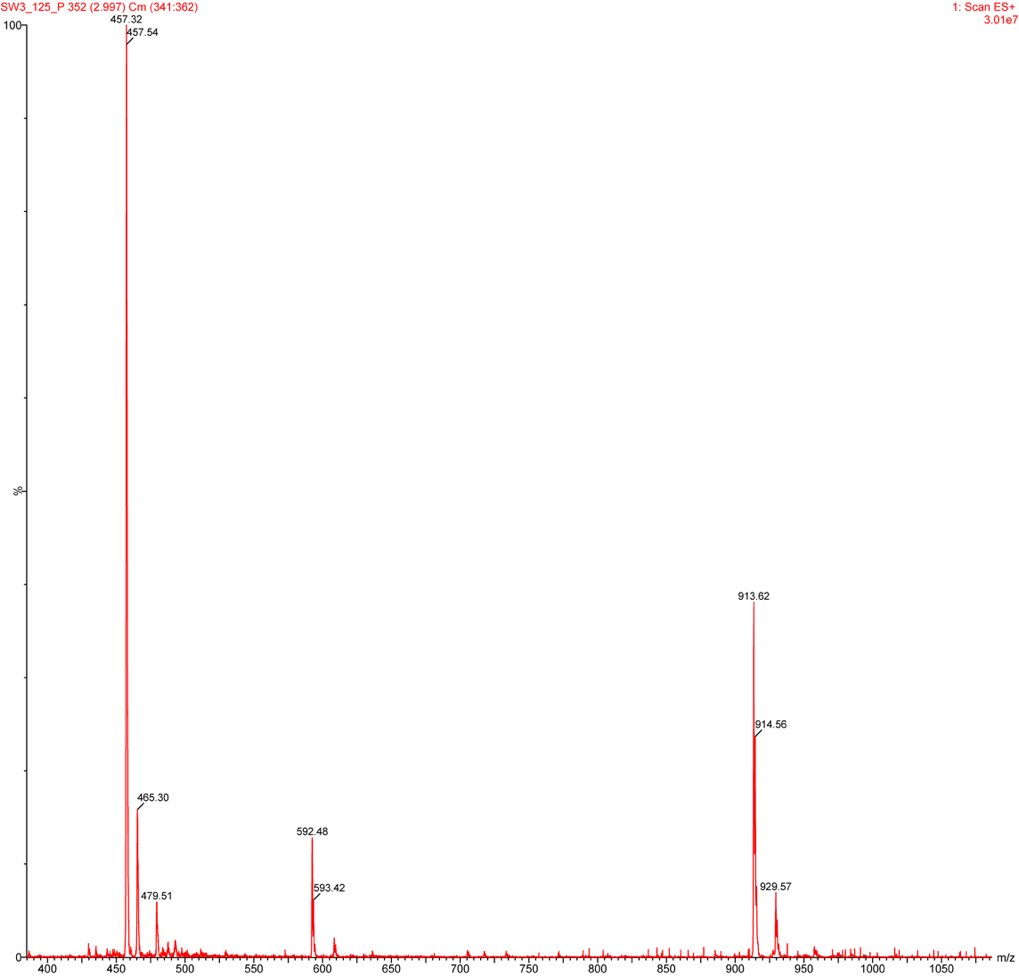


Characterization Data for compound **P1bP**. LC/MS top: UV traces bottom: low resolution mass spectrum. LR-ESI-MS: m/z calc. for [M+H]^+^.: 913.6; found: 913.6, [M+2H]^2+^: 457.3.

LC-MS Spectrum of compound **P1bP_B**

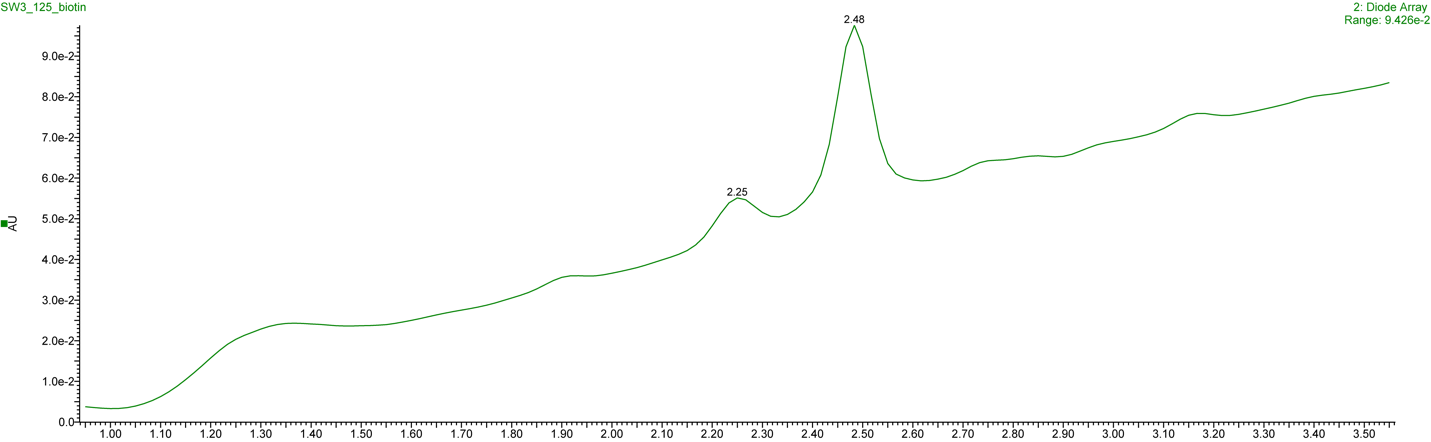


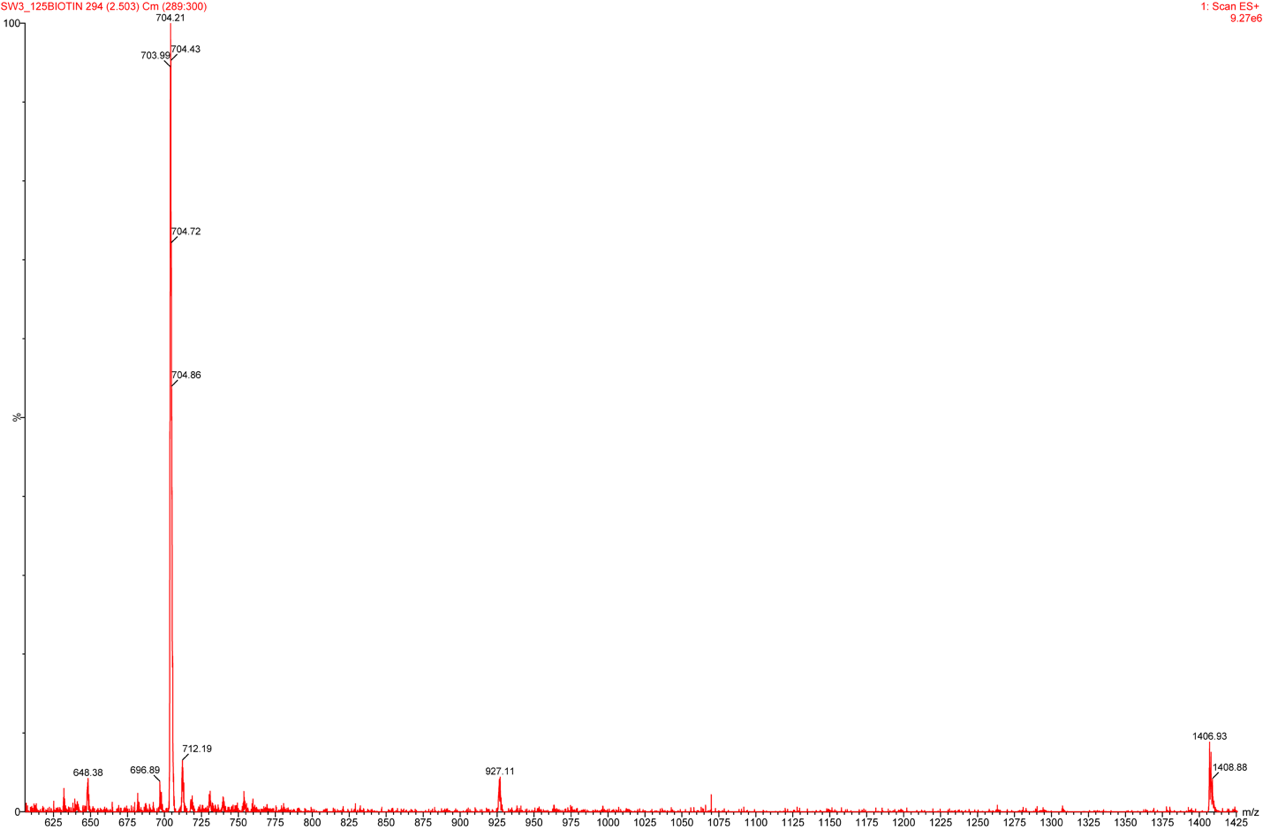


Characterization Data for compound **P1bP_B**. LC/MS top: UV traces bottom: low resolution mass spectrum. LR-ESI-MS: m/z calc. for [M+H]^+^.: 1406.8, found [: 1406.9, [M+2H]^2+^: 704.2.

LC-MS Spectrum of compound **SW2_110A_P**

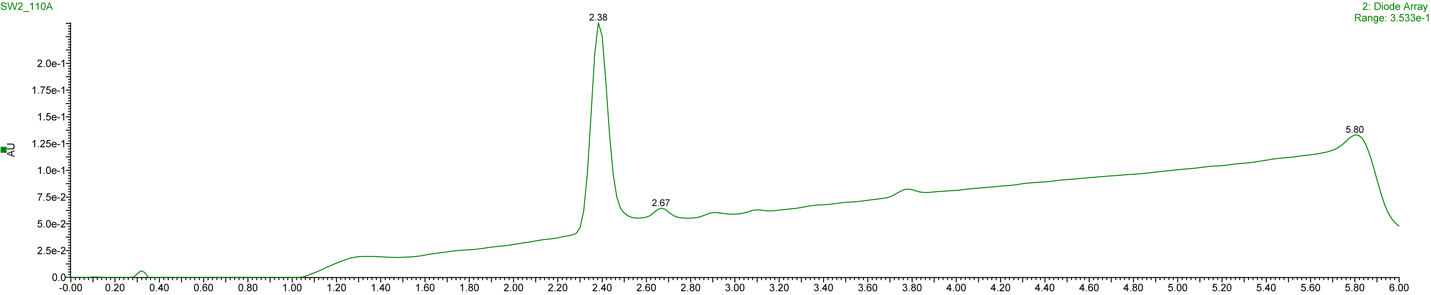


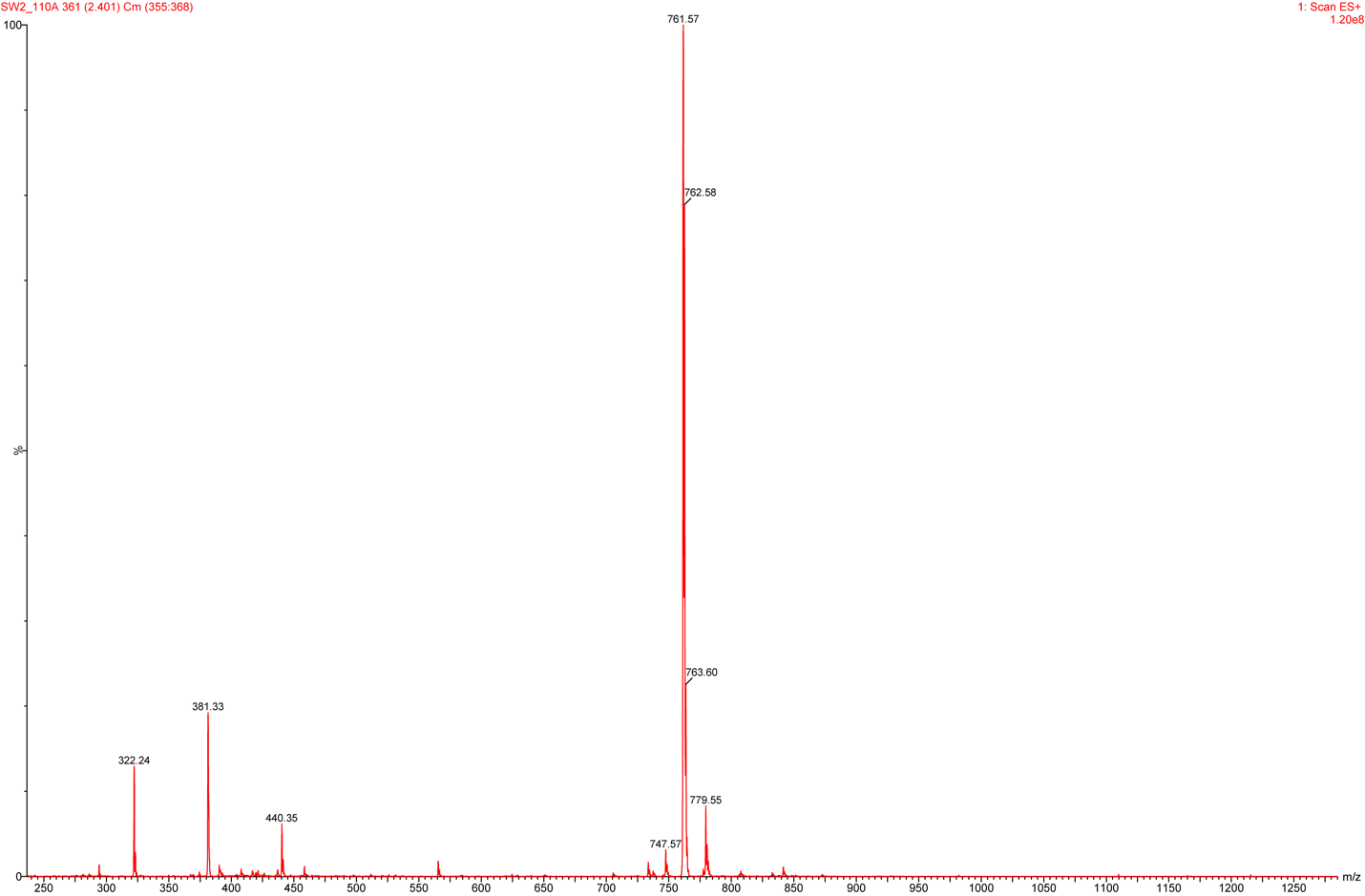


Characterization Data for compound **SW2_110A_P**. LC/MS top: UV traces bottom: low resolution mass spectrum. LR-ESI-MS: m/z calc. for [M+H]^+^: 761.5; found: 761., [M+2H]^2+^: 381.3.

LC-MS Spectrum of compound **SW2_110A_B**

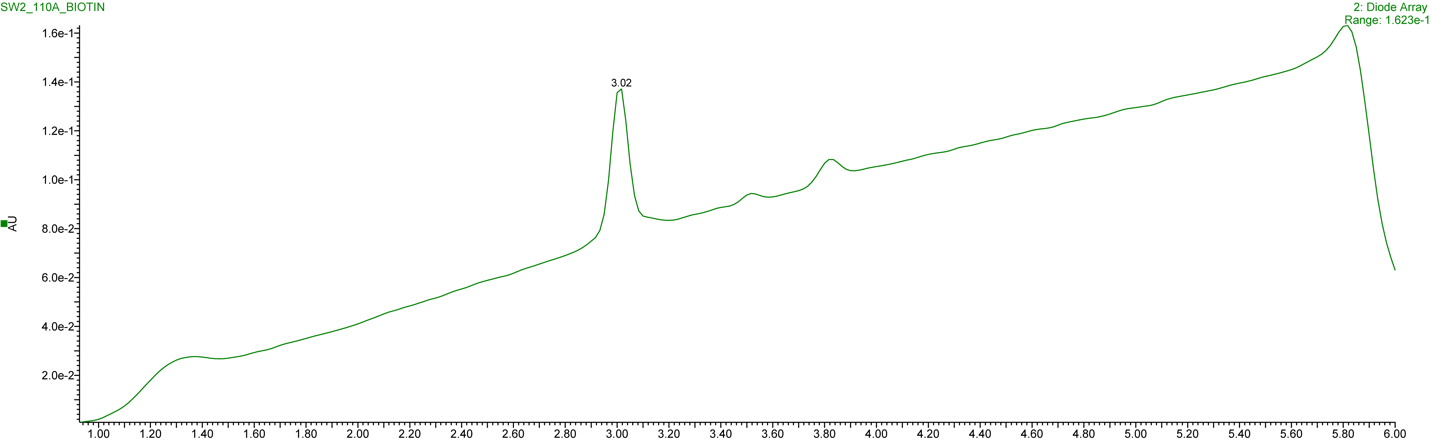


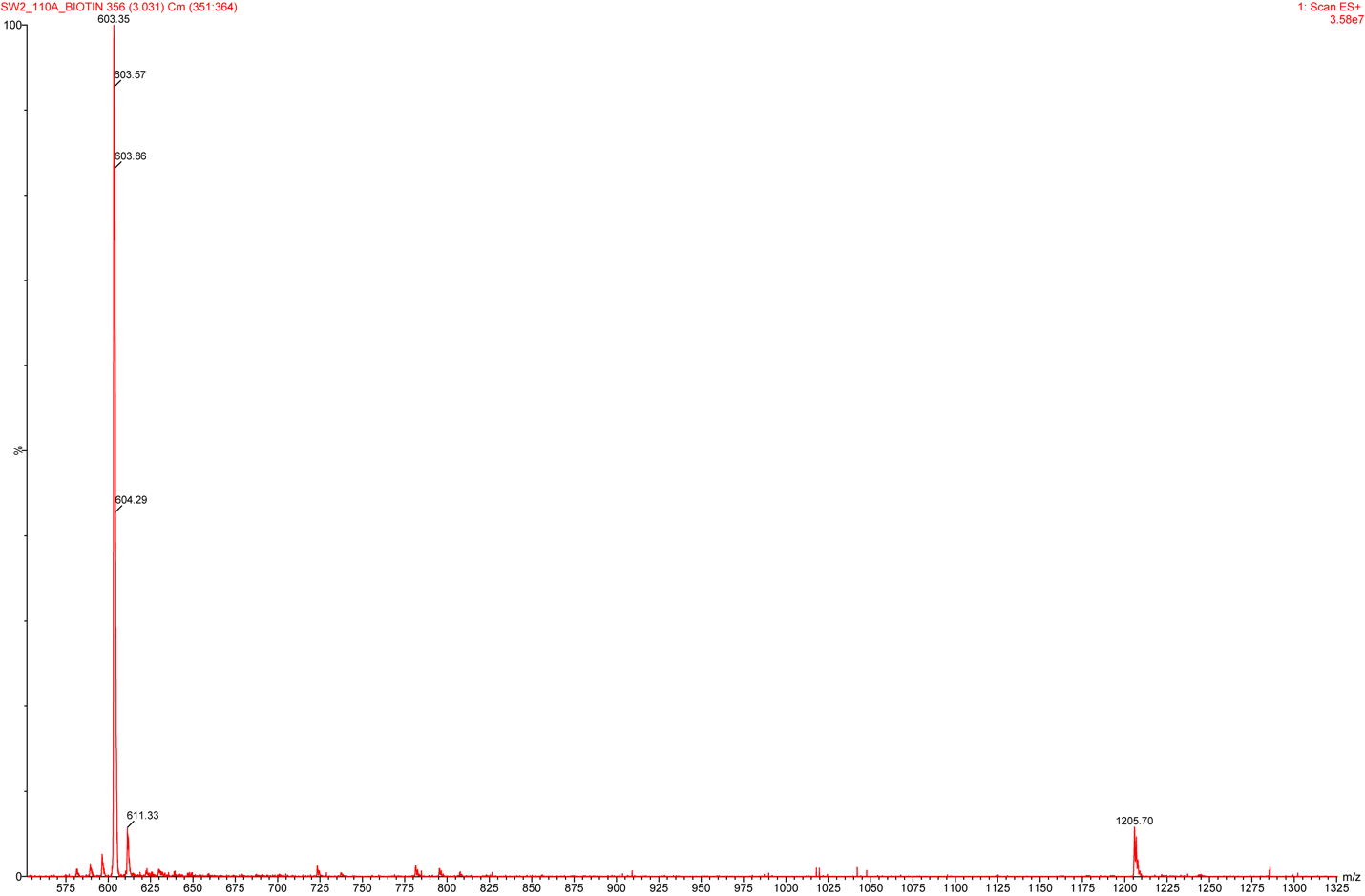


Characterization Data for compound **SW2_110A_B**. LC/MS top: UV traces bottom: low resolution mass spectrum. LR-ESI-MS: m/z calc. for [M+H]^+^.: 1205.7; found: 1205.7, [M+2H]^2+^: 603.4.

LC-MS Spectrum of Compound **P2LP**


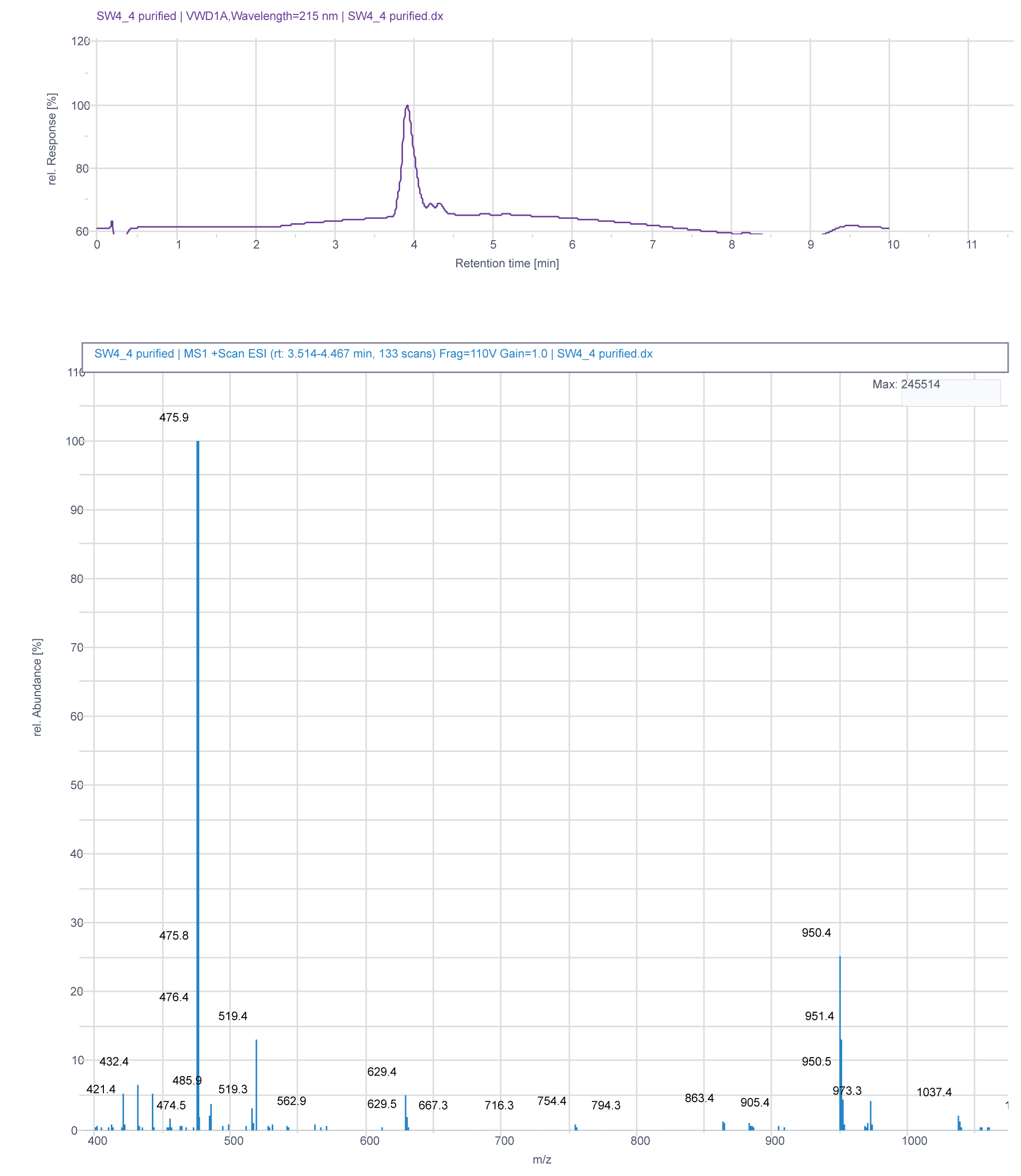


Characterization Data for compound **P2LP**. LC/MS top: UV traces bottom: low resolution mass spectrum. LR-ESI-MS: m/z calc. for [M+H]^+^.: 950.5; found: 950.4, [M+H2]^2+^: 475.9.

LC-MS Spectrum of Compound **P2P**

*
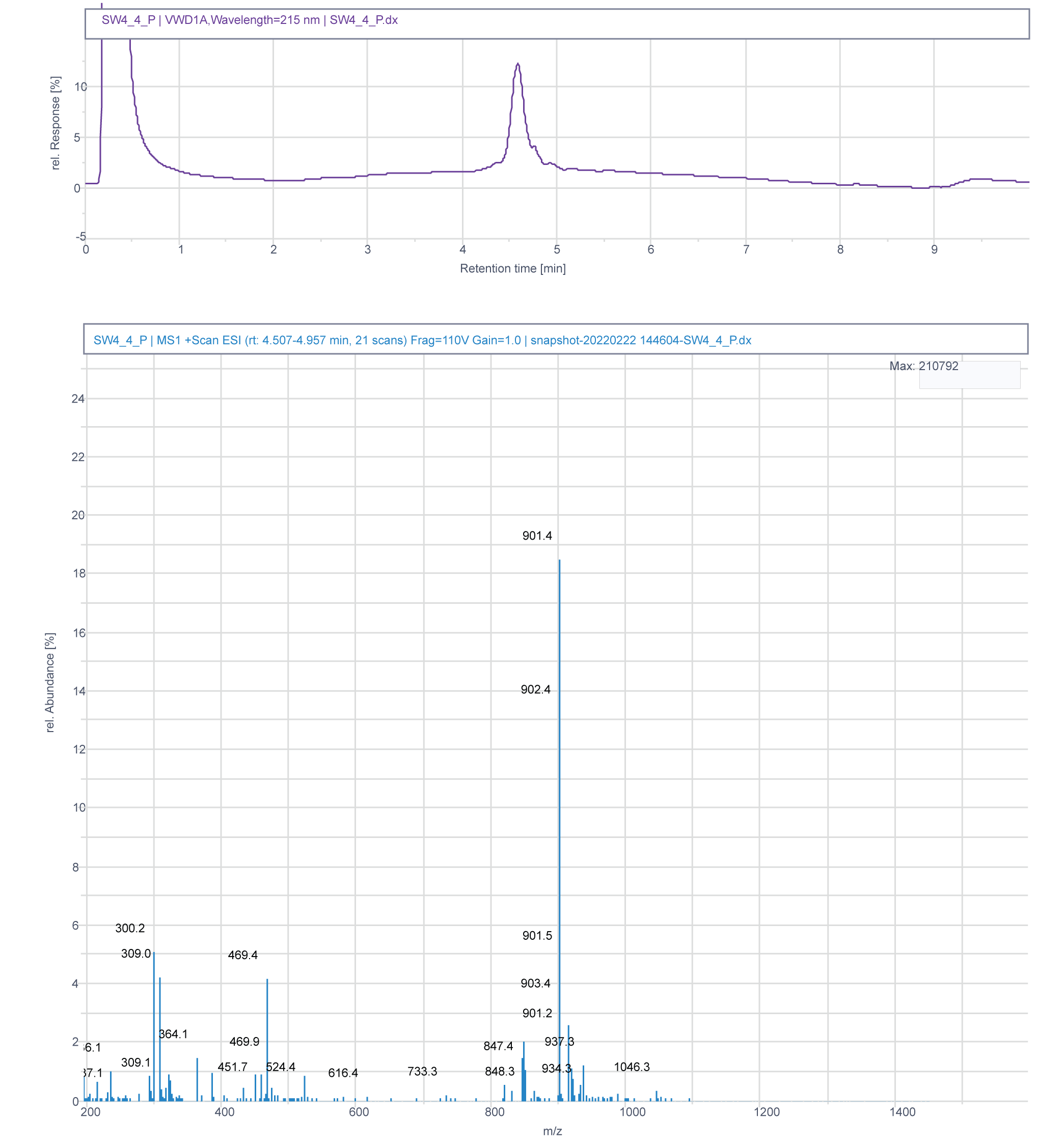
*

Characterization Data for compound **P2**. LC/MS top: UV traces bottom: low resolution mass spectrum. LR-ESI-MS: m/z calc. for [M+H]^+^.: 901.5; found: 901.4.

LC/MS of product of imine 1 with 3-methylindole **16**


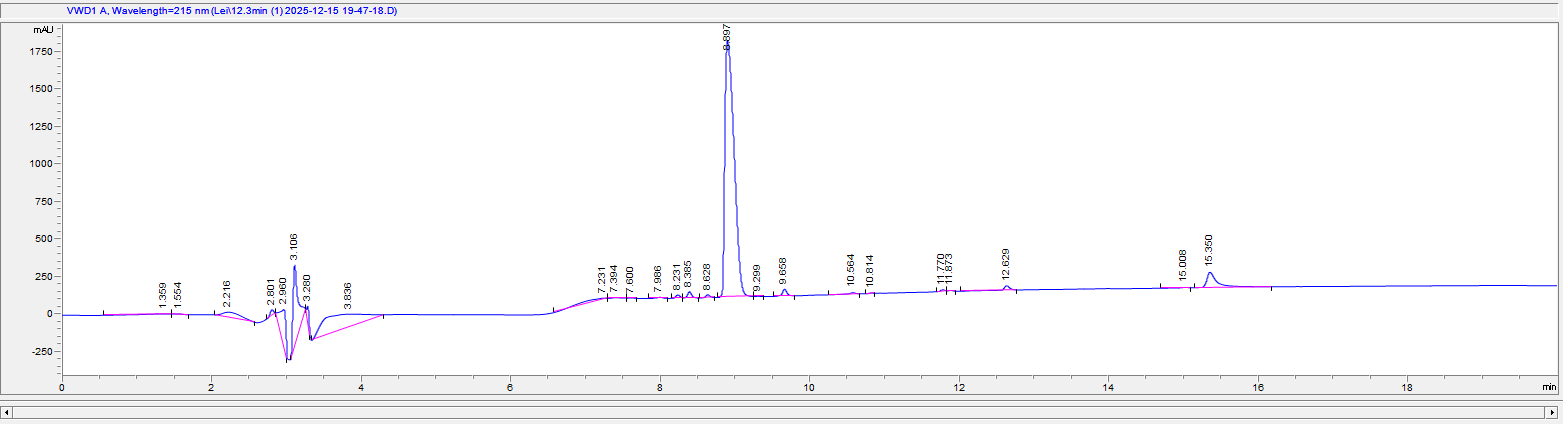

Characterization Data for compound **16**. LC/MS top: UV trace at 215 nm. bottom: low resolution mass spectrum. LR-ESI-MS: m/z calc. for [M+H]^+^.: 284.2; found: 284.2.

LC/MS of sulfonamide imine-N-acetyl-l-tyrosine-methyl amide **17**


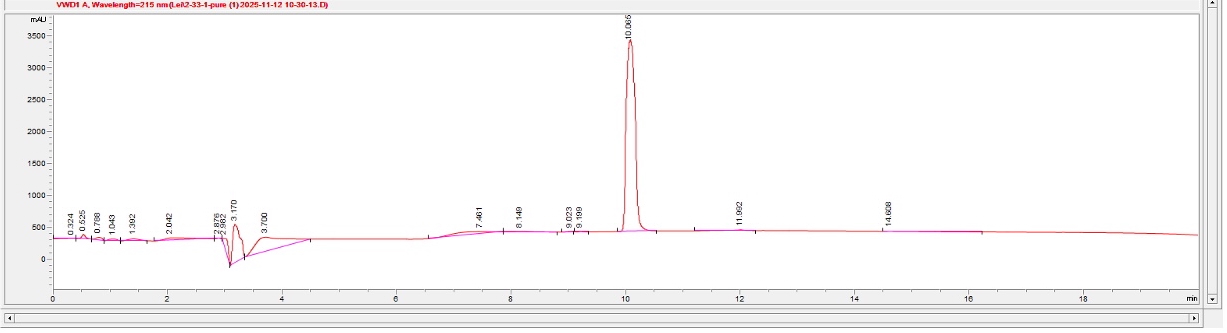


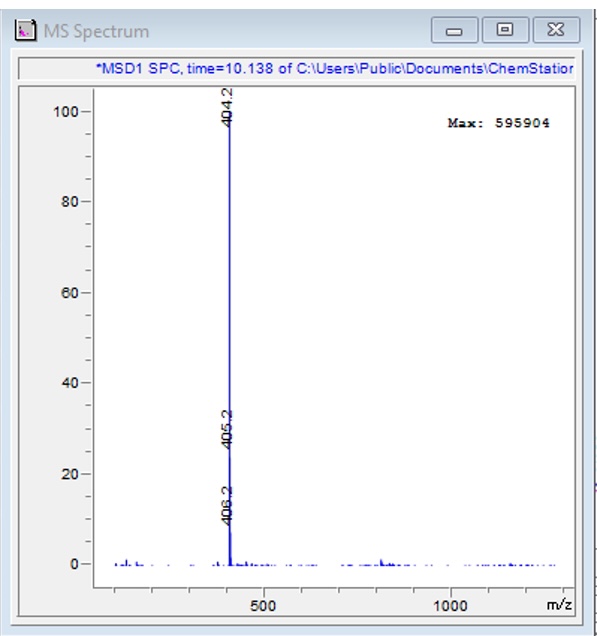


Characterization Data for compound **17**. LC/MS top: UV trace at 215 nm. bottom: low resolution mass spectrum. LR-ESI-MS: m/z calc. for [M+H]^+^.: 404.1; found: 404.2.

LC/MS of 20mer ssDNA-P1BL Conjugate

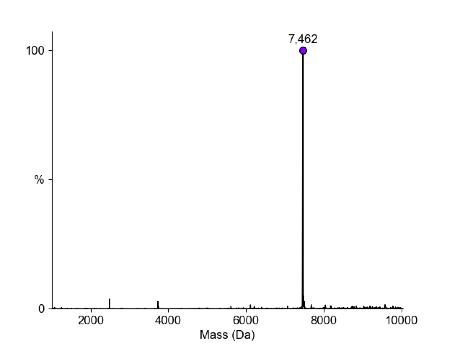


Characterization Data for 20mer ssDNA-P1BL Conjugate. LC/MS top: UV traces bottom: deconvoluted mass spectrum. Expected mass: 7467; Mass found: 7462.

1. **Supplementary References**

(1) Wang, S.; Denton, K. E.; Hobbs, K. F.; Weaver, T.; McFarlane, J. M. B.; Connelly, K. E.; Gignac, M. C.; Milosevich, N.; Hof, F.; Paci, I.; Musselman, C. A.; Dykhuizen, E. C.; Krusemark, C. J. Optimization of Ligands Using Focused DNA-Encoded Libraries to Develop a Selective, Cell-Permeable CBX8 Chromodomain Inhibitor. *ACS Chem. Biol.* **2020**, *15* (1). https://doi.org/10.1021/acschembio.9b00654.

(2) Kaustov, L.; Ouyang, H.; Amaya, M.; Lemak, A.; Nady, N.; Duan, S.; Wasney, G. A.; Li, Z.; Vedadi, M.; Schapira, M.; Min, J.; Arrowsmith, C. H. Recognition and Specificity Determinants of the Human Cbx Chromodomains. *J. Biol. Chem.* **2011**. https://doi.org/10.1074/jbc.M110.191411.

(3) Tyanova, S.; Temu, T.; Sinitcyn, P.; Carlson, A.; Hein, M. Y.; Geiger, T.; Mann, M.; Cox, J. The Perseus Computational Platform for Comprehensive Analysis of (Prote)Omics Data. *Nat. Methods* **2016**, *13* (9), 731–740. https://doi.org/10.1038/nmeth.3901.

(4) Ten Brink, H. T.; Meijer, J. T.; Geel, R. V.; Damen, M.; Löwik, D. W. P. M.; Van Hest, J. C. M. Solid-Phase Synthesis of C-Terminally Modified Peptides. *J. Pept. Sci.* **2006**. https://doi.org/10.1002/psc.780.

(5) Minakawa, M.; Guo, H. M.; Tanaka, F. Imines That React with Phenols in Water over a Wide PH Range. *J. Org. Chem.* **2008**, *73* (21), 8669–8672. https://doi.org/10.1021/JO8017389/ASSET/IMAGES/LARGE/JO-2008-017389_0001.JPEG.

(6) Nakata, H.; Imai, T.; Yokoshima, S.; Fukuyama, T. A SIMPLE CHIRAL TEMPLATE FOR THE SYNTHESIS OF FUNCTIONALIZED α-ARYLGLYCINE DERIVATIVES. *Heterocycles* **2008**, *76* (1), 747–757.

(7) Denton, K. E.; Wang, S.; Gignac, M. C.; Milosevich, N.; Hof, F.; Dykhuizen, E. C.; Krusemark, C. J. Robustness of In Vitro Selection Assays of DNA-Encoded Peptidomimetic Ligands to CBX7 and CBX8. *SLAS Discov.* **2018**. https://doi.org/10.1177/2472555217750871.

(8) Wang, S.; Denton, K. E.; Hobbs, K. F.; Weaver, T.; McFarlane, J. M. B.; Connelly, K. E.; Gignac, M. C.; Milosevich, N.; Hof, F.; Paci, I.; Musselman, C. A.; Dykhuizen, E. C.; Krusemark, C. J. Optimization of Ligands Using Focused DNA-Encoded Libraries to Develop a Selective, Cell-Permeable CBX8 Chromodomain Inhibitor. *ACS Chem. Biol.* **2020**, *15* (1), 112–131. https://doi.org/10.1021/acschembio.9b00654.

(9) Wang, S.; Alpsoy, A.; Sood, S.; Ordonez-Rubiano, S. C.; Dhiman, A.; Sun, Y.; Jiao, G.; Krusemark, C. J.; Dykhuizen, E. C. A Potent, Selective CBX2 Chromodomain Ligand and Its Cellular Activity During Prostate Cancer Neuroendocrine Differentiation. *ChemBioChem* **2021**, *22*, 2335–2344.

(10) Rew, Y.; Goodman, M. Solid-Phase Synthesis of Amine-Bridged Cyclic Enkephalin Analogues via on-Resin Cyclization Utilizing the Fukuyama-Mitsunobu Reaction. *J. Org. Chem.* **2002**, *67* (25), 8820–8826. https://doi.org/10.1021/jo020447l.

(11) Sable, G. A.; Lee, K. J.; Shin, M. K.; Lim, H. S. Submonomer Strategy toward Divergent Solid-Phase Synthesis of α-ABpeptoids. *Org. Lett.* **2018**, *20* (9), 2526–2529. https://doi.org/10.1021/acs.orglett.8b00661.

(12) Fletcher, S. The Mitsunobu Reaction in the 21st Century. *Org. Chem. Front.* **2015**, *2* (6), 739–752. https://doi.org/10.1039/c5qo00016e.
